# Proteogenomics refines the molecular classification of chronic lymphocytic leukemia

**DOI:** 10.1038/s41467-022-33385-8

**Published:** 2022-10-20

**Authors:** Sophie A. Herbst, Mattias Vesterlund, Alexander J. Helmboldt, Rozbeh Jafari, Ioannis Siavelis, Matthias Stahl, Eva C. Schitter, Nora Liebers, Berit J. Brinkmann, Felix Czernilofsky, Tobias Roider, Peter-Martin Bruch, Murat Iskar, Adam Kittai, Ying Huang, Junyan Lu, Sarah Richter, Georgios Mermelekas, Husen Muhammad Umer, Mareike Knoll, Carolin Kolb, Angela Lenze, Xiaofang Cao, Cecilia Österholm, Linus Wahnschaffe, Carmen Herling, Sebastian Scheinost, Matthias Ganzinger, Larry Mansouri, Katharina Kriegsmann, Mark Kriegsmann, Simon Anders, Marc Zapatka, Giovanni Del Poeta, Antonella Zucchetto, Riccardo Bomben, Valter Gattei, Peter Dreger, Jennifer Woyach, Marco Herling, Carsten Müller-Tidow, Richard Rosenquist, Stephan Stilgenbauer, Thorsten Zenz, Wolfgang Huber, Eugen Tausch, Janne Lehtiö, Sascha Dietrich

**Affiliations:** 1 Department of Medicine V, Hematology, Oncology and Rheumatology, University of Heidelberg, Heidelberg, Germany; 2 European Molecular Biology Laboratory (EMBL), Heidelberg, Germany; 3 Molecular Medicine Partnership Unit (MMPU), Heidelberg, Germany; 4 Department of Translational Medical Oncology, National Center for Tumor Diseases (NCT) Heidelberg and German Cancer Research Center (DKFZ), Heidelberg, Germany; 5 Faculty of Biosciences, University of Heidelberg, Heidelberg, Germany; 6 Department of Oncology-Pathology, Karolinska Institute and Science for Life Laboratory, Stockholm, Sweden; 7 Clinical Cooperation Unit Molecular Hematology/Oncology, German Cancer Research Center (DKFZ), Heidelberg, Germany; 8 Division of Molecular Genetics, German Cancer Research Center (DKFZ), Heidelberg, Germany; 9 Department of Internal Medicine, Division of Hematology, The Ohio State University, Columbus, OH USA; 10 Department of Molecular Medicine and Surgery, Karolinska Institutet, Stockholm, Sweden; 11 Department I of Internal Medicine, Center for Integrated Oncology Aachen-Bonn-Cologne-Duesseldorf (CIO ABCD), Excellence Cluster for Cellular Stress Response and Aging-Associated Diseases (CECAD), Center for Molecular Medicine Cologne (CMMC), University of Cologne, Cologne, Germany; 12 Institute of Medical Biometry and Informatics, Heidelberg University, Heidelberg, Germany; 13 Institute of Pathology, University of Heidelberg, Heidelberg, Germany; 14 Center for Molecular Biology of the University of Heidelberg (ZMBH), Heidelberg, Germany; 15 Division of Hematology, University of Tor Vergata, Rome, Italy; 16 Clinical and Experimental Onco-Hematology Unit, Centro di Riferimento Oncologico di Aviano (CRO) IRCCS, Aviano, Italy; 17 Clinical Genetics, Karolinska University Laboratory, Karolinska University Hospital, Stockholm, Sweden; 18 Department of Internal Medicine III, University of Ulm, Ulm, Germany; 19 Department of Medical Oncology and Hematology, University Hospital Zürich, Zürich, Switzerland; 20 Department of Hematolgy, Oncology and Immunolgy, University Hospital of Düsseldorf, Düsseldorf, Germany

**Keywords:** Chronic lymphocytic leukaemia, Computational models, Proteomic analysis

## Abstract

Cancer heterogeneity at the proteome level may explain differences in therapy response and prognosis beyond the currently established genomic and transcriptomic-based diagnostics. The relevance of proteomics for disease classifications remains to be established in clinically heterogeneous cancer entities such as chronic lymphocytic leukemia (CLL). Here, we characterize the proteome and transcriptome alongside genetic and ex-vivo drug response profiling in a clinically annotated CLL discovery cohort (n = 68). Unsupervised clustering of the proteome data reveals six subgroups. Five of these proteomic groups are associated with genetic features, while one group is only detectable at the proteome level. This new group is characterized by accelerated disease progression, high spliceosomal protein abundances associated with aberrant splicing, and low B cell receptor signaling protein abundances (ASB-CLL). Classifiers developed to identify ASB-CLL based on its characteristic proteome or splicing signature in two independent cohorts (n = 165, n = 169) confirm that ASB-CLL comprises about 20% of CLL patients. The inferior overall survival in ASB-CLL is also independent of both *TP53-* and IGHV mutation status. Our multi-omics analysis refines the classification of CLL and highlights the potential of proteomics to improve cancer patient stratification beyond genetic and transcriptomic profiling.

## Abstract (web summary)

Proteomics can be used to refine cancer classification. Here, the authors characterise chronic lymphocytic leukaemia patients by proteogenomics, and identified a subtype of patients with poor prognosis associated with aberrant B cell receptor signalling.

## Introduction

Chronic lymphocytic leukemia (CLL) is the most common adult leukemia in Western countries. It is characterized by the accumulation of mature B lymphocytes in the peripheral blood, the bone marrow, and lymph nodes. This incurable malignancy has a very heterogeneous clinical course. Some patients can be followed with a “watch and wait” strategy for many years, while others need frequent treatments and have a shorter overall survival^1^.

The cell of origin of CLL represents an important source of heterogeneity, which is marked by the mutation status of the immunoglobulin heavy variable (IGHV) genes and characteristic methylation profiles^2–4^. CLL with unmutated IGHV genes (U-CLL) cases progress faster and have worse outcomes than IGHV-mutated CLL (M-CLL) cases^5^. These differences in clinical behavior between M-CLL and U-CLL are partially determined by differences in responsiveness to B-cell receptor (BcR) stimulation^6^. Inhibition of the BcR pathway has revolutionized the treatment landscape of CLL^7^ and the prognostic difference between U-CLL and M-CLL is diminished in patients treated with BcR inhibitors^8^.

Recently, the genetic landscape of CLL has been well characterized^9^, which partially explains the heterogeneous disease courses. The most frequent recurrent somatic mutations in CLL alter genes encoding for components of a small set of oncogenic pathways^10^. These include the DNA damage response pathway, pathways that receive input from the microenvironment (NOTCH, Toll-like receptor, and CD40 signaling), and pathways affecting ribosomal processing^11^. Additionally, mutations in central splicing components, most frequently in *SF3B1*, are drivers of CLL^12^. Recurrent structural chromosomal aberrations, including deletions of 13(q14), 11(q22-23), 17(p13), and trisomy 12, are routinely measured before the initiation of treatment and are detected in approximately 80% of CLL patients. They are associated with diverse biological phenotypes and contrasting clinical behavior^13^, but the pathophysiology of some of these variants such as trisomy 12 is still insufficiently understood.

A multi-omics analysis integrating the genome, transcriptome, and clinical outcomes could offer a valuable tool to fill this gap and improve the understanding of genetic variants and outcomes in CLL. Recent developments in mass spectrometry have facilitated deep proteomic profiling of multiple tumor specimens in parallel. The integration of proteomics with genomics and other data layers has started to enhance our understanding of selected cancer entities^14–21^. However, the proteome-centric data integration to clinically relevant endpoints and functional phenotypes has been mostly lacking.

Although the proteome of CLL B cells has been compared to non-malignant normal B cells^22–24^, and proteomic insights from reverse-phase protein arrays have been linked to clinical data^25–27^, a systematic description of the proteomic landscape of CLL integrating genome, transcriptome, and clinical datasets is currently limited^25,27^. Here, we employed in-depth high-resolution isoelectric focusing liquid chromatography–mass spectrometry (HiRIEF-LC–MS)^28^ based proteomics to connect the proteomic landscape of a clinically well-characterized and representative cohort of 68 CLL patients with genome, transcriptome, and drug perturbation profiling. Among the six identified proteome-based subgroups, we discovered a previously unknown subset of CLL patients with poor outcome. This subgroup was characterized by a high abundance of spliceosomal proteins. We could link spliceosomal protein abundances to aberrant splicing and poor outcome in CLL. We validated the subgroups in further independent cohorts using multiple validation strategies. Most importantly, we took advantage of the higher throughput of data-independent acquisition (DIA) proteomics to confirm our findings from the in-depth HiRIEF dataset. Including all patients used for the different validation strategies, our study contains data from 1503 CLL patients (233 for which proteomics was performed) and directly links the observed molecular phenotypes to clinical outcome.

## Results

### Study outline

**Fig. 1:**
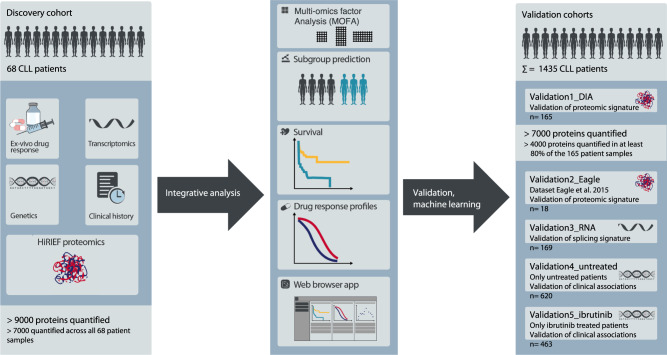
Overview of the study design. In-depth proteomics, transcriptomics, genetic and drug perturbation profiling were performed for a discovery cohort of 68 clinically well-characterized CLL patients. An integrative analysis was performed to describe associations between the different molecular layers and to uncover patient subgroups. These were validated in multiple independent cohorts using different validation strategies. To validate proteomic signatures, we performed data-independent acquisition (DIA) proteomics on 165 CLL patient samples (Validation1_DIA) and took advantage of a published cohort of 18 CLL samples (Validation2_Eagle). For the validation of splicing signatures, 169 additional patients were characterized by RNA-sequencing (Validation3_RNA). We linked proteomic signatures with genetic profiles, which allowed us to validate associations of major biological axes in CLL with clinical outcome in 620 untreated (Validation4_untreated) and 463 ibrutinib-treated CLL patients (Validation5_ibrutinib). In total, this study analyzed data from 1503 CLL patients.

We assembled a discovery cohort of 68 well-characterized CLL patients with a representative distribution of CLL subgroups^9,13^ (Fig. 1, Supplementary Fig. 1a, Supplementary Tables 1 and 2). Viably frozen CLL samples were CD19 enriched and split into three aliquots: one for proteomics (Supplementary Data 1), one for RNA sequencing, and one for DNA panel sequencing^29^. We further characterized functional phenotypes of the same CLL samples by ex-vivo drug response profiling using a high-throughput microscopy-based drug screening platform with a panel of 43 drugs, most of them FDA approved or currently in clinical trials (e.g. ibrutinib, venetoclax)^10^.

We used multiple validation strategies to confirm associations between genetic features, the proteome, splicing signatures, and clinical outcome in independent CLL cohorts (Fig. 1, Supplementary Tables 1 and 2). This included data-independent acquisition (DIA) based mass spectrometry profiles of 165 well-characterized CLL patient samples (Fig. S1b, Validation1_DIA cohort), 18 CLL proteomes previously published (Validation2_Eagle,^24^), 169 CLL transcriptomes (Validation3_RNA cohort), and two clinical validation cohorts of 620 untreated CLL patients (Validation4_untreated cohort) and 463 ibrutinib-treated CLL patients (Validation5_ibrutinib).

To facilitate further analyses, we set up an open-access, user-friendly web application for others to explore and mine this comprehensive characterization of the relationship between the proteome, recurrent genetic aberrations, and the transcriptome of CLL, and consequences for drug response and clinical outcome (https://www.dietrichlab.de/CLL_Proteomics/).

### Genotype-molecular phenotype connections with functional consequences

**Fig. 2:**
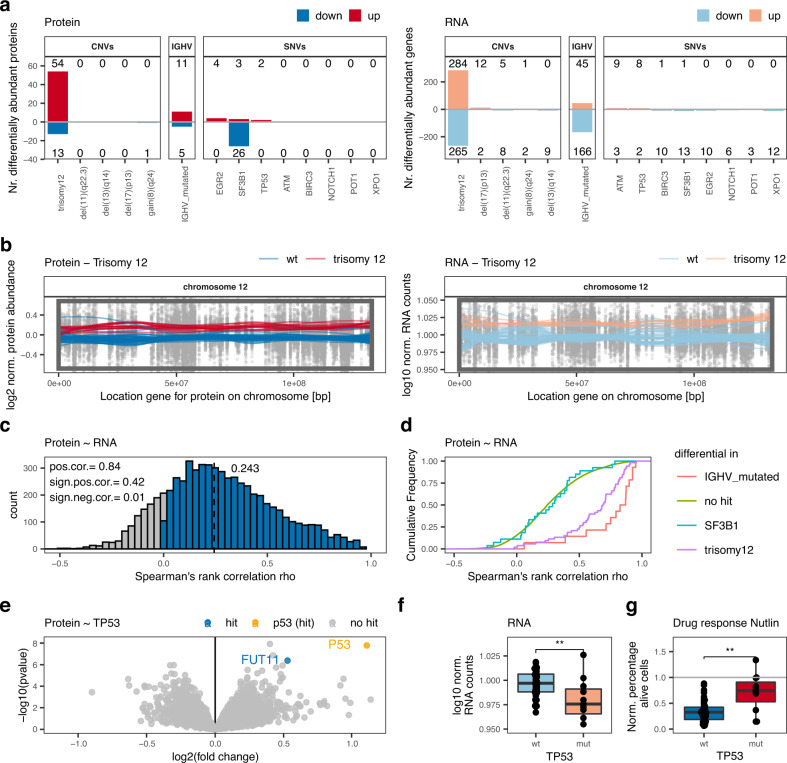
Interplay between genetic alterations, proteomics, and transcriptomics. **a** Number of significantly differentially abundant proteins (left; FDR < 0.1%; |log2FC| > 0.5) and differentially expressed genes (right; FDR < 0.1%; |log2FC| > 1.5) in relation to recurrent genetic alterations. **b** Levels of both proteins and transcripts from chromosome 12 and the impact of trisomy 12. Normalized protein abundance (left panel) and gene expression levels (right panel) for chromosome 12 are shown. Points represent individual values for protein/gene–patient pairs. Lines are locally weighted scatterplot smoothed values for individual patients with (red) or without (blue) trisomy 12. The box is the region affected by trisomy 12. **c** Distribution of Spearman’s rank correlations for protein-mRNA pairs. **d** Cumulative density distribution of protein-mRNA Spearman’s rank correlations for the proteins significantly differentially abundant in IGHV mutated (red), trisomy 12 (pink) or *SF3B1*-mutated (blue) CLL in comparison to all other proteins without these associations (green). **e** Volcano plot indicating differential proteins in *TP53* mutated in comparison to *TP53* wild-type CLL; hit = adjusted *p* < 0.001, |log2FC|>0.5. *P* values calculated by limma. Multiple testing correction by FDR to identify hits. **f** *TP53* transcript levels in *TP53* mutated (mut, *n* = 11) and wild-type (wt, *n* = 47) biologically independent CLL samples; two-sided Wilcoxon signed-rank test *p* = 0.005. **g** Percentages, normalized to solvent control, of alive cells of *TP53* mutated (mut, *n* = 56) and wild-type (wt, *n* = 11) biologically independent CLL samples treated ex-vivo with 9 µM nutlin 3a; two-sided Wilcoxon signed-rank test, *p* = 0.0019. Boxplots are represented as first and third quartiles with a median in the center. Whiskers are defined as 1.5 times the interquartile range (**f** and **g**). Source data are provided as a Source Data file.

To explore genotype-phenotype relationships, we investigated associations between known recurrent genetic alterations of CLL^9,10^, mRNA expression, and protein abundance. We first analyzed protein abundance with respect to recurrent genetic aberrations of CLL and found that trisomy 12 had the strongest impact on differential protein abundance in comparison to other genetic lesions (Fig. 2a). First, we applied a very stringent FDR of 0.1% and found 54 proteins significantly up- and 13 proteins downregulated in trisomy 12 positive CLL. IGHV mutation status (19 proteins, among them ZAP70) and *SF3B1* mutations (29 proteins) were also associated with multiple differentially abundant proteins. With a less stringent FDR of 5% we found similar trends (Supplementary Fig. 1c, d). Next, we analyzed gene expression with respect to recurrent genetic alterations of CLL (FDR 0.1%, Fig. 2a; FDR 5% Fig. S1e). Again, trisomy 12 (549 transcripts at FDR 0.1%) and IGHV mutation status (205 transcripts at FDR 0.1%) were associated with many significant gene expression changes. In contrast, *SF3B1* mutations were associated with fewer differentially expressed genes than with differentially abundant proteins (14 transcripts at FDR 0.1%).

We further investigated if changes in protein abundance associated with trisomy 12 were related to gene dosage effects. While trisomy 12 increased the abundance of proteins located on chromosome 12 as expected (Fig. 2b), 63% of the proteins identified as differentially abundant were expressed by genes located on other chromosomes. Similar gene dosage effects affecting mRNA and protein levels were also observed for other important structural aberrations of CLL, e.g. deletion of 13(q14), 11(q22-23), 17(p13), and gain of 8(q24) (Supplementary Fig. 2a, b). As shown for other cancers, our data confirm in CLL that gene dosage effects translate into transcriptomic as well as proteomic changes^14,15,17,30^.

Even though alterations like trisomy 12 or the presence of somatic hypermutations translated into changes in levels of both mRNA and protein, overall correlation between protein abundance and corresponding mRNA levels was low (Fig. 2c; median correlation = 0.243). This is comparable to gene ~ protein correlations reported for other cancers, suggesting wide post-translational regulation of cancer proteomes^14,15,17^. Out of all quantified genes and corresponding proteins, 42% showed significant, positive correlations (BH adjusted *p* < 0.05). Differentially abundant proteins associated with trisomy 12 or IGHV mutation status exhibited higher mRNA ~ protein correlations than unassociated proteins (Fig. 2d; median correlation trisomy 12 associated proteins = 0.69; IGHV associated proteins = 0.86). This was not the case for differentially abundant proteins associated with *SF3B1* mutations (Fig. 2d; median correlation = 0.29). These findings further demonstrate a direct link between protein abundance changes and gene expression changes in trisomy 12 and IGHV-mutated CLL, while the tumorigenic effect of *SF3B1* mutations is caused by post-transcriptional mechanisms.

Although *TP53*, *ATM*, and *XPO1* mutations were associated with relatively few differentially abundant proteins, we detected specific and biologically relevant protein abundance changes. p53 was the most upregulated protein in *TP53*-mutated compared to wild-type samples (Fig. 2e). It is well-established that not only loss-of-function but also gain-of-function mutations in *TP53* can contribute to tumor progression^31^. Our results are consistent with the finding that these tumors accumulate high levels of mutant p53, contributing to gain-of-function properties^32^. In contrast, *TP53* transcripts were significantly downregulated in *TP53*-mutated CLL samples (Fig. 2f), indicating that post-transcriptional mechanisms are responsible for the accumulation of mutant p53 in CLL, as suggested for other blood cancers^32^. The ex-vivo drug response screen revealed that *TP53-*mutated CLL samples responded worse to chemotherapy and the MDM2 inhibitor nutlin 3a than *TP53* wild-type samples (Fig. 2g), as expected^32^. This example illustrates how multi-omics profiling can be used to trace the effect of a somatic mutation on the transcriptome, the proteome, and finally on the functional consequences for drug response.

We further found that protein levels of the tumor suppressor ATM and the nuclear transport protein XPO1 were lower in mutated than in wild-type samples (Supplementary Fig. 2c, d). Both associations were only observed on protein but not on transcript level (Supplementary Fig. 2c, d).

Together, our data show that the CLL proteome not only shows many changes that also are present on mRNA level, but in addition uncovers biological relationships not apparent from the transcriptome.

### Integrative analysis reveals proteomics factor associated with outcome

**Fig. 3:**
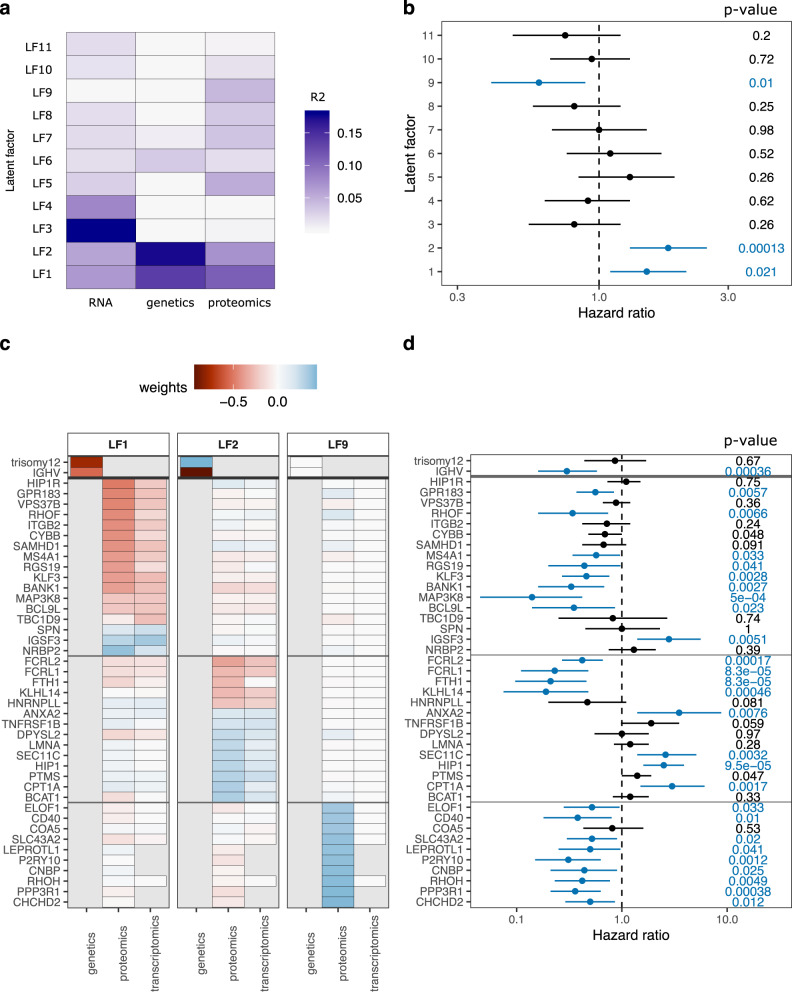
Multi-omics factor analysis (MOFA) of proteogenomics dataset. **a** MOFA of proteome, transcriptome, and genome dataset identified 11 latent factors (LF) each explaining at least 1.5% of variance. Explained variances per factor and dataset are color-coded. **b** Hazard ratios from Cox regression of LFs with time to next treatment (TTNT). LF1, LF2, and LF9 were significantly (FDR < 10%, blue) associated with TTNT. *P* values (Wald-test) are shown on the right. Mean and 95% confidence intervals are shown. *n* = 61 biologically independent patient samples. **c** Genes, transcripts, and proteins with the strongest weights loaded onto LF1, LF2, and LF9. Weights were scaled between genetics (divided by two), proteomics, and transcriptomics (times ten) to achieve similar ranges. **d** Hazard ratios from Cox regression for TTNT with genes and proteins with strong weights for LF1, LF2, and LF9. Significant associations (*p* < 0.05) are colored in blue. *P* values (Wald-test) are shown on the right. Mean and 95% confidence intervals are shown. *n* = 72 biologically independent patient samples. Source data are provided as a Source Data file.

We performed an unbiased, unsupervised multi-omics factor analysis (MOFA^33^) to obtain an integrative view of covariations across multiple datasets. MOFA revealed eleven latent factors (LF) explaining at least 1.5% of variance each (Fig. 3a). Only LF1, LF2, and LF9 were significantly associated with time to next treatment (TTNT, Fig. 3b). LF1 and LF2 were active in all data layers (proteome, RNA and genetics) and were mainly driven by trisomy 12 and IGHV mutation status in the genetics data (Fig. 3c). The proteins loaded onto LF1 and LF2 were not only significantly associated with TTNT in the discovery cohort (Fig. 3d), but also showed a significant association with overall survival in our DIA proteomics validation cohort (Validation1_DIA; Supplementary Fig. 3). Interestingly, LF9 was nearly exclusively active in the proteomics data and was apart from LF1 and LF2 the only factor significantly associated with clinical outcome. Hence, MOFA revealed that proteomics profiling uncovers clinically relevant biology that was not detected with the other data layers.

### Proteomics-based stratification of CLL identifies six distinct subgroups

**Fig. 4:**
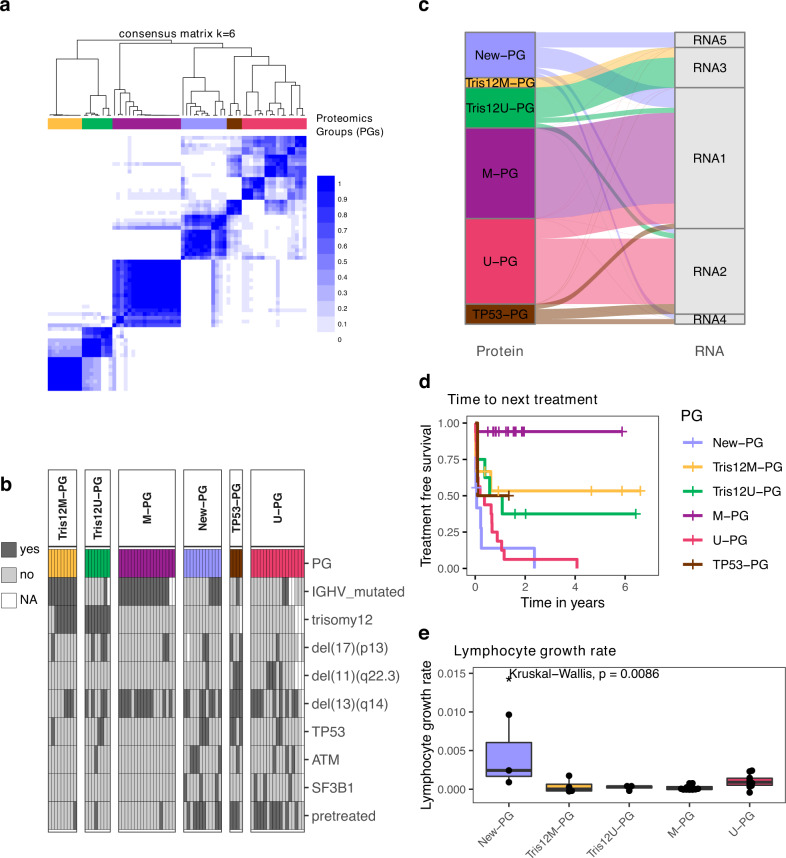
Consensus clustering of proteomics profiles partitioned CLL into six subgroups. **a** Consensus clustering partitioned CLL samples into six proteomics groups (PGs). The color scale indicates which proportion of iterations patients clustered together. **b** Annotation of PGs with genetic alterations and pretreatment status. **c** Comparison of proteomic-based with transcriptomic-based consensus clustering. *n* = 58 biologically independent samples. **d** The PGs had significantly different times to next treatment (TTNT; log-rank test, *p* < 0.0001). Only patients for whom TTNT was available are shown. **e** Lymphocyte growth rates for Tris12M-PG (*n* = 4), Tris12U-PG (*n* = 4), M-PG (*n* = 14), U-PG (*n* = 10), and New-PG (*n* = 3) biologically independent samples. Kruskal–Wallis test *p* = 0.0086. No data for TP53-PG was available. Boxplots are represented as first and third quartiles with a median in the center. Whiskers are defined as 1.5 times the interquartile range. Source data are provided as a Source Data file.

We discovered associations of the proteomics layer and clinical outcome (TTNT), which could not be found on the transcript or on the genetic level. Therefore, we decided to explore the proteomics layer in depth and in an unbiased manner. To describe similarities and differences between protein profiles of patients, we performed consensus clustering of the protein dataset. This revealed six proteomics groups (PG; Fig. 4a, Supplementary Fig. 4a, b). T-distributed stochastic neighbor embedding (t-SNE) and principal component analysis supported the partition into these subgroups (Supplementary Fig. 4c, d).

Next, we analyzed if any genetic alterations were associated with the different PGs. We found that four PGs represented trisomy 12/M-CLL (Tris12M-PG, *n* = 9), trisomy 12/U-CLL (Tris12U-PG, *n* = 8), M-CLL (M-PG, *n* = 18), and U-CLL (U-PG, *n* = 17, Fig. 4b; Fisher’s exact test, FDR < 10%). Of note, one Tris12M-PG patient annotated as trisomy 12 negative, harbored a subclonal trisomy 12. Tris12M-PG, Tris12U-PG, and M-PG were enriched for untreated patients. A fifth subgroup, TP53-PG, comprised only 4 patient samples, but three of the four samples harbored a *TP53* mutation (Fig. 4b; Fisher’s exact test, FDR < 10%). In accordance with the discovery of LF9, which was exclusively active in the proteomic layer, we additionally found a new proteomics-based subgroup, which in contrast to all other subgroups did not show any association with known recurrent genetic alterations (New-PG). Only trisomy 12 was depleted in the new PG.

To relate these proteomics-based groups to the transcriptome, we performed consensus clustering of corresponding transcriptome data. The transcriptomic-based subgroups only partially overlapped with the proteomic groups (Fig. 4c): There was correspondence between RNA groups 1–3 and the proteomics groups Tris12U-PG, Tris12M-PG, M-PG, and U-PG, but the new subgroup without a genetic annotation (New-PG) and TP53-PG (*TP53*) were split across multiple transcriptomic subgroups.

We further investigated drug response profiles of all PGs. Although effect sizes differed, the groups responded to the majority of CLL-relevant drugs ex-vivo. Only the *TP53*-mutated group TP53-PG exerted poor overall response to many drugs including chemotherapeutic agents (Supplementary Fig. 4e, f).

The proteomic-based grouping separated patients with different TTNT, demonstrating the clinical relevance of the subgroups (Fig. 4d). Although the new PG was not enriched for high-risk factors such as mutated *TP53* and unmutated IGHV genes, it had the shortest TTNT (Fig. 4d) and the fastest in vivo lymphocyte doubling time of all PGs, which indicated an increased proliferative capacity of these tumors (Kruskal–Wallis test, *p* = 0.009, Fig. 4e). In contrast, M-PG (M-CLL, no trisomy 12) had a significantly longer TTNT than all other subgroups as expected.

Taken together, unsupervised clustering of relative protein abundances partitioned CLL patients into six biologically and clinically relevant groups, of which five could be explained by known genetic characteristics while a new subgroup with poor outcome was only identifiable based on proteomics.

### Dysregulated cellular processes of the new poor outcome proteomics group ASB-CLL

**Fig. 5:**
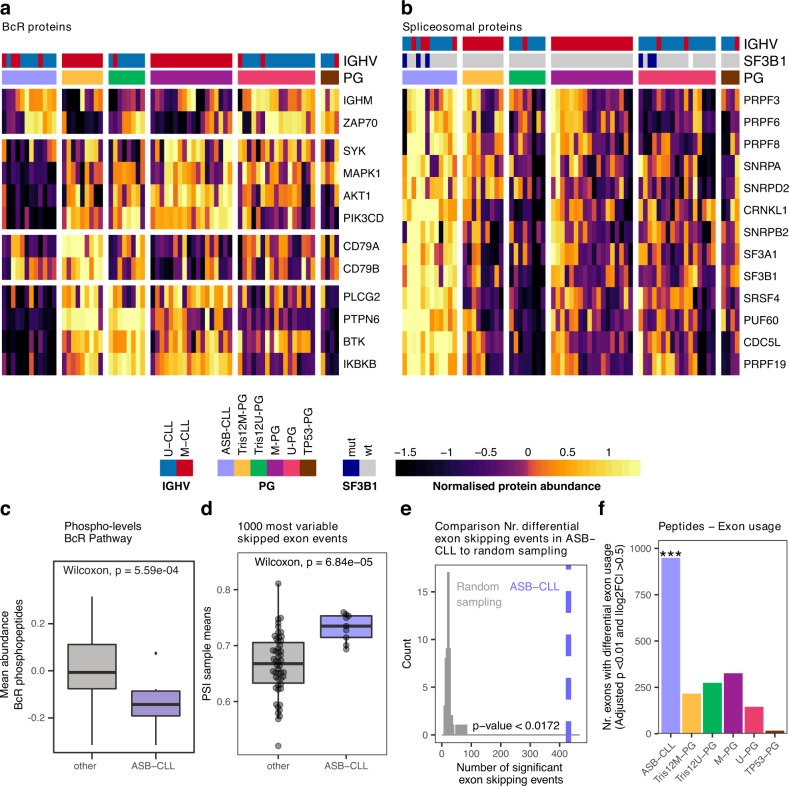
Characterization of the new proteomics group ASB-CLL. **a** Heatmaps of scaled log2 protein abundances for selected BcR proteins and **b** spliceosome components. Patients were grouped according to PG and the proteins were clustered hierarchically. Based on these profiles the group “New-PG” was renamed to “ASB-CLL” (**A**ltered **S**pliceosome, low **B**cR signaling proteins **CLL**) **c** The mean relative intensity of phosphorylated peptides uniquely mapping to proteins whose corresponding genes belong to the gene set: KEGG_B_CELL_RECEPTOR_SIGNALING and which could be quantified in at least 50% of TMT-channels were calculated per sample. 12 biologically independent ASB-CLL patient samples were compared to 56 other biologically independent patient samples. **d** Mean percent spliced-in (PSI) value per patient calculated from the 1000 most variable exon skipping events across all patients of the discovery cohort. 9 biologically independent ASB-CLL patient samples were compared to 50 other biologically independent patient samples. Boxplots are represented as first and third quartiles with a median in the center. Whiskers are defined as 1.5 times the interquartile range (**c** and **d**). **e** Number of significant differential exon skipping events between ASB-CLL and all other groups for the actual PG assignment (blue line) and random PG permutations (gray distribution)**. f** Peptide-based differential exon usage per PG (FDR 1%, |logFC| > 0.5). One-sided fisher exact test corrected for multiple testing using the FDR method was used to assess differences between the groups (each group vs all others). For ASB-CLL the adjusted *p* value = 1.89E−239. Source data are provided as a Source Data file.

The new poor outcome subgroup comprised approximately 20% of our discovery cohort and could only be identified at the protein level. To describe which pathways and processes were altered in this group, we analyzed differential protein abundances between the new and other PGs. Enrichment analysis identified BcR signaling proteins such as BTK, PLCG2, and PIK3CD among the most downregulated proteins in the New-PG (Fig. 5a, Supplementary Fig. 5a, b, Supplementary Data 2). Surprisingly, downregulation of these central BcR signaling components in the New-PG was independent of the IGHV mutation status, despite the latter being an important surrogate for BcR activity in CLL^34^. We further measured the ex-vivo response of the samples to ibrutinib in a co-culture model of stromal cells and CLL cells, which suggested that the New-PG responded less to the BcR inhibitor ibrutinib (Supplementary Fig. 5c, Ibrutinib: 40 nM, Wilcoxon signed-rank test *p* = 0.01). Additionally, phosphorylation levels of BcR signaling proteins exhibited a similar downregulation in the New-PG (Fig. 5c).

The New-PG was further characterized by upregulation of enzymes involved in the degradation of branched chain amino acids (BCAA; Supplementary Fig. 5d, e) and downregulation of proteasomal proteins (Supplementary Fig. 5f, g). Most importantly, proteins associated with the spliceosome were most significantly upregulated in the New-PG (Fig. 5b, Supplementary Fig. 5h, i, Supplementary Data 2). We therefore named this group ASB-CLL (**A**ltered **S**pliceosome, low **B**cR signaling proteins **CLL**). We and others observed very low correlation between protein- and corresponding mRNA levels of components of the spliceosome^14,17^ (median *ρ* = −0.015; Supplementary Fig. 6a), which might explain why ASB-CLL was not detectable at the transcriptome level.

Splice factor mutations (e.g. *SF3B1* mutations) are recurrent in CLL^10^; however, mutations of *SF3B1* were neither enriched nor depleted in ASB-CLL (BH adjusted *p* = 0.39). SF3B1 protein levels were not different between *SF3B1*-mutated and wild-type CLL cases, but were significantly higher in ASB-CLL than in other subgroups (Supplementary Fig. 6b, c). It has recently been reported that the main tumorigenic effect of *SF3B1* mutations is mediated by inclusion of a poison-exon in the tumor suppressor gene *BRD9* followed by downregulation of BRD9^35^. We could confirm mis-splicing (Supplementary Fig. 6d) and downregulation (Supplementary Fig. 6e) of BRD9 in *SF3B1*-mutated cancers, but neither mis-splicing nor downregulation of BRD9 was detected for ASB-CLL (Supplementary Fig. 6f, g). For ten out of the twelve ASB-CLL patients we further performed whole-exome sequencing of tumor and matched normal controls, to explore whether any additional somatic mutations in spliceosomal proteins could be found for this subgroup. Except for a *DDX5* mutation in a *SF3B1*-mutated patient we could not detect any other splice factor mutations (Supplementary Fig. 6h). Together these results suggest that the increased spliceosomal protein abundance detected in ASB-CLL is independent of mutations in the spliceosome.

To further characterize if upregulation of the spliceosome in ASB-CLL had any functional consequences, we analyzed alternative splicing on mRNA and peptide level for this group. We started with an unbiased approach and annotated all possible alternative splicing events across all patients using the transcriptome data and rMATS^36^. Within each category (skipped exons, 3′ and 5′ alternative splice site usage, retained introns, mutually exclusive exons) we calculated the per patient mean percent spliced-in (PSI) values from the 1000 alternative splicing events that showed the highest variability across all patients (Supplementary Fig. 6i). Comparisons between ASB-CLL and all other subgroups revealed a distinct exon usage profile for ASB-CLL, with fewer skipped exons, and more 3′ and 5′ alternative splice site usage (Fig. 5d, Supplementary Fig. 6i). Moreover, we observed a statistically significant correlation between the average spliceosomal protein abundance and the aforementioned mean PSI values (SE: *ρ* = 0.34, *p* = 0.008; 3′ASS: *ρ* = 0.44, *p* = 0.0005; 5′ASS: *ρ* = 0.42, *p* = 0.0009; also note the similar patterns in Supplementary Fig. 5i and Supplementary Fig. 6i). This observed statistical association may be the consequence of an underlying causal relation, which needs to be explored in future studies. In total, we detected 427 exon skipping events with a significantly distinct alternative splicing pattern of ASB-CLL versus all other proteomics subgroups (FDR < 1%, absolute difference of groupwise mean PSI values >0.1, Supplementary Data 3). These exon skipping events were significantly more frequent in ASB-CLL than expected by chance (permutation test, *p* < 0.0172, Fig. 5e). No preference of the differential exon skipping events for coding or untranslated regions was observed (Fisher’s exact test; *p* value = 0.45). Significantly different exon usage by ASB-CLL was also detected on peptide level (Fig. 5f, Supplementary Data 3).

Thus, ASB-CLL was characterized by low abundance of BcR pathway components, lower phosphorylation levels of BcR components, and altered spliceosome function.

### Validation of ASB-CLL in independent cohorts

**Fig. 6:**
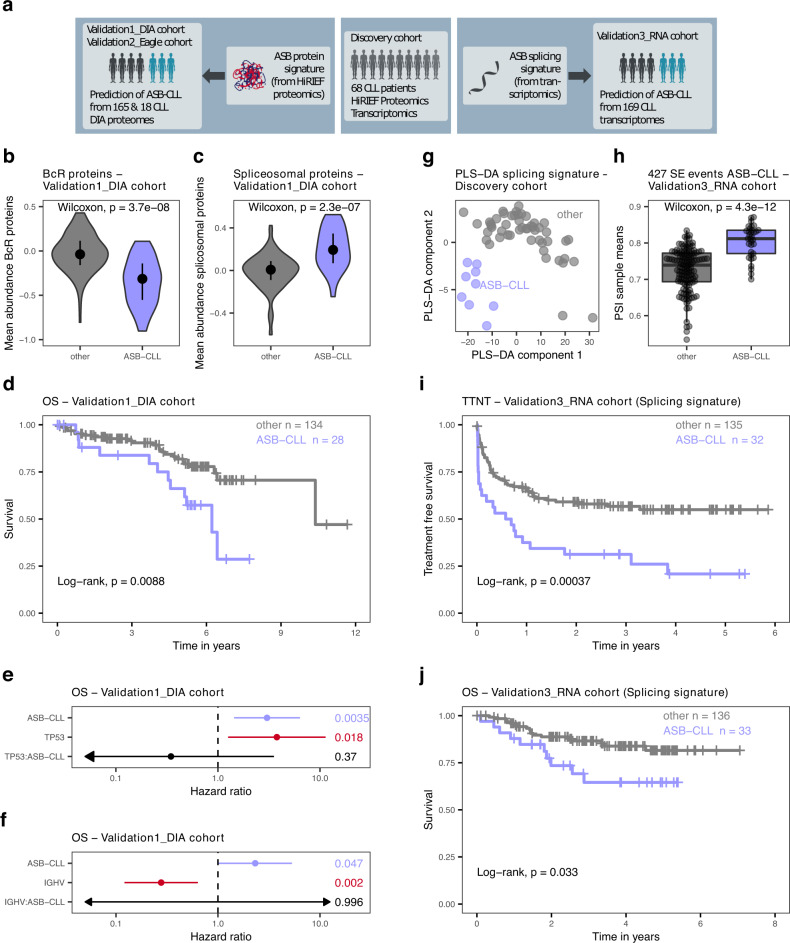
Validation of the ASB-CLL group. **a** A classifier was trained from the proteomics dataset of the discovery cohort and applied to a DIA proteomics validation dataset (Validation1_DIA cohort, 165 patients; left part of figure) and a published cohort (Validation2_Eagle, 18 patients)^24^. An additional classifier was trained on the splicing signature of ASB-CLL using the transcriptomics dataset from the discovery cohort and applied to a transcriptomic validation dataset (Validation3_RNA cohort, 169 patients). Violin plots of BcR protein abundances (**b**) and spliceosomal proteins (**c**) comparing the subgroup identified as ASB-CLL in the Validation1_DIA dataset (n = 28) to all other patients (*n* = 134). Two-sided Wilcoxon signed-rank test, central bar denotes first and third quartile, central dot denotes median. **d** Overall survival (OS) of Validation1_DIA cohort, divided into ASB-CLL and all other patients. Log-rank test, *p* = 0.0088. Hazard ratios from Cox regression of OS in a model including either TP53 aberrations (del(17)(p13) or TP53 mutations), ASB-CLL, and their interaction (TP53:ASB-CLL) (*n* = 158) (**e**) or IGHV mutation status, ASB-CLL, and their interaction (IGHV:ASB-CLL) (*n* = 154) (**f**). *P* values (Wald-test) are shown on the right. Mean and 95% confidence intervals are shown. Arrows indicate that the confidence interval extends beyond the shown length. **g** Patient samples of the discovery cohort plotted in the plane of the first two components computed by the partial least squares discriminant analysis (PLS-DA) of the discovery cohort. The predictive performance of the classifier was assessed using leave-one-out cross validation with the area under the ROC curve being estimated as 0.97. **h** Mean PSI value per patient of the Validation3_RNA cohort based on the 427 significant differential exon skipping events identified in the discovery cohort. Boxplots are represented as first and third quartiles with a median in the center. Whiskers are defined as 1.5 times the interquartile range. Time to next treatment (TTNT) (**i**) and OS (**j**) of patients from the Validation3_RNA cohort, classified as ASB-CLL or other based on the characteristic splicing signature of ASB-CLL. Log-rank test, *p* = 0.00037 (**i**), *p* = 0.033 (**j**). Source data are provided as a Source Data file.

We confirmed the existence and prognostic relevance of the ASB-CLL PG in additional cohorts of CLL patients (Fig. 6a): We validated the proteomics signature associated with ASB-CLL by performing proteomics using data-independent acquisition (DIA) based mass spectrometry analysis of 165 patients (Validation1_DIA). We also confirmed the proteomics signature in an independent, previously published cohort (Validation2_Eagle)^24^. We further validated the splicing signature characteristic for ASB-CLL in an independent cohort of 169 CLL patients (Validation3_RNA cohort).

To robustly detect ASB-CLL in DIA proteomics, we trained a k-top scoring pairs (k-TSP) classifier on all BcR and spliceosomal proteins in the HiRIEF dataset. We applied the classifier to the Validation1_DIA cohort and identified 28 ASB-CLL patients (10 untreated, 15 pretreated, 3 unknown), corresponding to 17% of patients. This proportion of ASB-CLL was similar to the training dataset. As expected, BcR proteins were down- and spliceosomal proteins were upregulated in ASB-CLL (Fig. 6b, c; Supplementary Fig. 7, Wilcoxon signed-rank test *p* = *p* < 3.7 × 10^−8^, *p* < 2.3 × 10^−7^). Additionally, ASB-CLL was characterized by upregulation of BCAA proteins and downregulation of proteasomal proteins, which is in accordance with the findings observed in the discovery cohort (Supplementary Fig. 8a, b; Wilcoxon signed-rank test *p* = 0.002 and *p* < 2.2 × 10^−6^). Most importantly, ASB-CLL patients had significantly worse overall survival (log-rank test, *p* < 0.0088; Fig. 6d) and this was independent of the risk associated with *TP53* aberrations (del(17)(p13) or *TP53* mutations) and unmutated IGHV genes (Fig. 6e, f). The poor overall survival of ASB-CLL was confirmed in the subgroup of samples obtained from treatment-naive patients (log-rank test, *p* = 0.041; Supplementary Fig. 8c). Application of the k-TSP classifier to an independently published cohort by Eagle and colleagues (Validation2_Eagle)^24^ comprising nine U-CLL and nine M-CLL patients identified a subgroup of five patients with a genetic and proteomics profile similar to ASB-CLL (Supplementary Fig. 8d–g). Further, we applied the ktsp classifier to a proteomics dataset comprising patients overlapping with our study (30 out of 91 CLL patients), but which was independently generated in a different laboratory using DIA proteomics^27^. In total 9 of the 91 CLL samples (10%) were classified as ASB-CLL and we could confirm the poor outcome of patients characterized by an ASB-CLL proteome profile (overall survival, log-rank test, *p* < 0.001).

Additionally, we used an independent cohort of patients (Validation3_RNA) for which paired-end RNA-sequencing was performed to validate the splicing signature detected for ASB-CLL. A partial least squares discriminant analysis (PLS-DA) classifier was trained on the discovery cohort samples using the previously identified 427 significantly differential exon skipping events as predictors (Fig. 6g). Predictive performance of the PLS-DA model was assessed by leave-one-out cross validation, which gave an estimate of 0.97 for the area under the classifier’s ROC curve. The PLS-DA model was then applied to the Validation3_RNA cohort to classify each of its 169 patients as either belonging to or not belonging to ASB-CLL. As observed in the training cohort, significantly larger mean PSI values of exon skipping events were detected for patients identified as belonging to ASB-CLL than for other patient groups (two-sided Wilcoxon rank-sum test, *p* = 4.3e−12, Fig. 6h). The ASB-CLL samples in the Validation3_RNA cohort exhibited the same splicing pattern as in the discovery cohort and also demonstrated a significantly shorter TTNT (log-rank test, *p* < 0.00037, Fig. 6i) and overall survival (log-rank test, *p* < 0.033, Fig. 6j).

### Cell of origin influences the effect of trisomy 12 on the proteome and clinical outcome

**Fig. 7:**
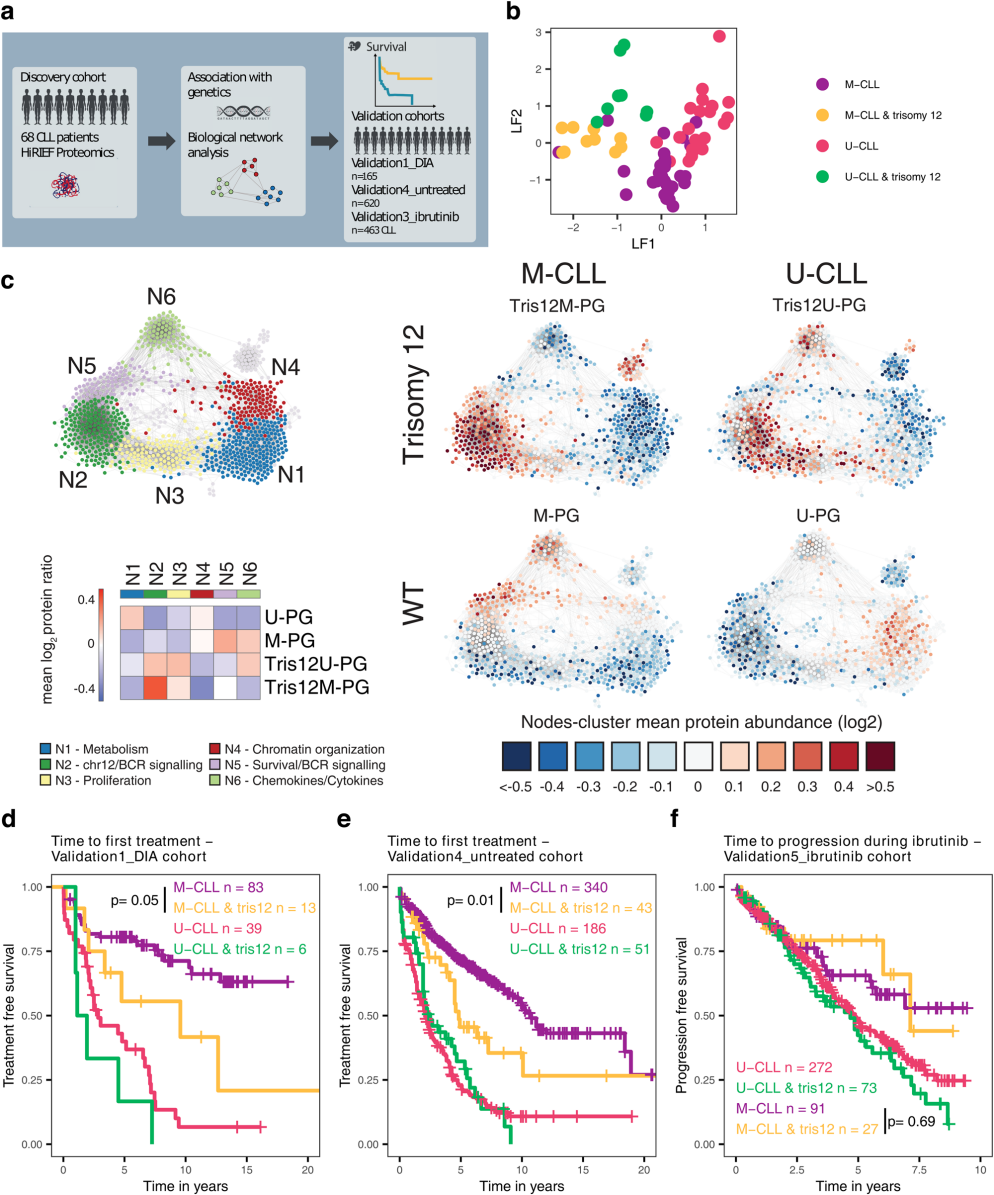
Relevance of the cell of origin for the influence of trisomy 12 on the CLL proteome and clinical outcome. **a** Validation strategy for Tris12M-PG, Tris12U-PG, M-PG, and U-PG. **b** Plot of loadings on LF1 and LF2 of individual patients. LF1 and LF2 could classify patients into 4 groups according to IGHV status and trisomy 12. **c** CLL protein correlation network analysis of HiRIEF dataset based on 1047 high-variance proteins. Protein groups were defined and color-coded based on modularity clustering (N1-N6) and enrichments detailed in Supplementary Fig. 9. A Heatmap of log2 mean relative protein abundances for all PGs for each modularity cluster (N1-N6) and node-cluster mean protein abundances for Tris12M-PG, Tris12U-PG, M-PG, and U-PG are shown. **d** Time to first treatment of patients from Validation1_DIA cohort, stratified into groups by IGHV mutation status and trisomy 12 (tris12). Trisomy 12 M-CLL patients had significantly faster disease progression than M-CLL patients without trisomy 12 (log-rank test, *p* = 0.05). **e** Time to first treatment of untreated patients from Validation4_untreated cohort, stratified into groups by IGHV mutation status and trisomy 12 (tris12). Trisomy 12 M-CLL patients had significantly faster disease progression than M-CLL patients without trisomy 12 (log-rank test, *p* = 0.01). **f** Time to progression of patients uniformly treated with ibrutinib (Validation5_ibrutinib), stratified into groups by IGHV mutation status and trisomy 12 (tris12). Trisomy 12 M-CLL patients did not have significantly faster disease progression than M-CLL patients without trisomy 12 (log-rank test, *p* = 0.69). Source data are provided as a Source Data file.

Plotting of the latent factors 1 and 2 from our MOFA resulted in the separation of four distinct groups (Tris12M-PG, Tris12U-PG, M-PG, U-PG), which were strongly associated with IGHV mutation status and trisomy 12 (Fig. 7a, b). Initially, we aimed to understand the functional consequences of this interaction for the proteome and subsequently used the association of these groups with trisomy 12 and IGHV mutation status to investigate survival differences between these subgroups in three independent datasets (Fig. 7a).

It is noteworthy that few cases fell into Tris12M-PG and Tris12U-PG although they were tested negative for trisomy 12 with a clone size of more than 20%. Interestingly one of these cases harbored a subclonal trisomy 12 lesion. These misclassifications could introduce a bias if the subgroups are identified based on the genetic features only.

To understand the global influence of IGHV status and trisomy 12 on the proteome, we performed a correlation network analysis^15^ of all samples in the discovery cohort independently of the subgroups to identify the most important biological modules of the CLL proteome (Fig. 7c). This revealed six biological modules (N1-N6) that were affected in all screened CLL patient samples. Next, we performed enrichment analysis for these modules and quantified the importance of each module in each PG (Fig. 7c, Supplementary Fig. 9a). All trends were confirmed by the networks with relative protein abundances from the DIA proteomics dataset of the Validation1_DIA cohort (*n* = 165 CLL patients, Supplementary Fig. 9b). As expected, the two trisomy 12 subgroups Tris12M-PG and Tris12U-PG displayed elevated levels of proteins located on chromosome 12 and proteins involved in BcR signaling (N2). We further assessed functional consequences of this upregulation for ex-vivo drug response and found that Tris12M-PG and Tris12U-PG were significantly more sensitive to BcR pathway inhibitors (e.g. ibrutinib, idelalisib) measured in an ex-vivo co-culture model of stroma- and CLL cells (Supplementary Fig. 9c).

Despite these similarities, there were also biologically relevant differences between Tris12M-PG and Tris12U-PG depending on the IGHV mutation background. The module containing proteins involved in chemokine and cytokine signaling (N6), was significantly more abundant in Tris12U-PG. By adjusting for U-CLL/M-CLL differences in cases without trisomy 12 we noted that U-CLL cases, in the context of trisomy 12, exhibited increased levels of MAPK signaling proteins while M-CLL cases had increased levels of cell adhesion molecules (Supplementary Fig. 9d). We could also identify differences in the BcR signaling pathway between M-CLL and U-CLL, specific to the context of trisomy 12; with U-CLL, trisomy 12 cases exhibiting a further increase of the intracellular components of the pathway (e.g. NFKB1, MAPK13), and M-CLL, trisomy 12 exhibiting an increase of membrane-bound components (e.g. CD21, CD79A/B, Supplementary Fig. 9e). This was also evident on the phosphorylation level where membrane-bound and membrane proximal proteins exhibited increased phosphorylation in the Tris12M-PG (e.g. CD19, LYN), while phosphorylation of intracellular components (e.g. MAP2K2, NFATC2, AKT2) was elevated in Tris12U-PG (Supplementary Fig. 9f).

Occurrence of trisomy 12 in M-CLL was associated with low abundance of proteins in N5, which have been shown to be related to cell survival^37^ (Supplementary Fig. 9a). In contrast, occurrence of trisomy 12 in U-CLL did not change protein levels in this module.

We further sought to investigate the impact of trisomy 12 on the natural course of M-CLL and U-CLL independently of any treatment context using time to first treatment (TTFT) as the endpoint. Trisomy 12 did not alter TTFT in U-CLL patients, but significantly decreased TTFT within the M-CLL patients (Validation1_DIA; Fig. 7d; log-rank test, *p* = 0.05). This could be confirmed in a cohort of 620 untreated CLL patients (Validation4_untreated; Fig. 7e; log-rank test, *p* = 0.01).

Interestingly, a faster progression of Tris12M-PG patients was not observed in a cohort of 463 CLL patients uniformly treated with ibrutinib (Validation5_ibrutinib; Fig. 7f; log-rank test, *p* = 0.69). This was true for patients who received ibrutinib as firstline treatment as well as relapse treatment (Supplementary Fig. 9g, h). However, patients with trisomy 12 had a better response to ibrutinib in vivo^38^ and ex-vivo (Supplementary Fig. 9c). Therefore, we hypothesize that the better response rate of trisomy 12 patients to BcR inhibitors balances out the faster progression observed in untreated Tris12M-PG patients.

These proteogenomics findings exemplify how the influence of a genetic alteration on the proteome varies depending on the cellular background in which it occurs, and how this interaction could translate into different clinical outcomes.

## Discussion

Cancer proteogenomic studies integrate proteomic and genomic data to gain understanding of the impact of genetic alterations on the proteome, both on particular proteins and proteome-wide effects. As proteins are considered the main effectors of many cellular processes this approach has recently been used to improve the understanding of the drivers of selected cancer entities^14–20^.

We applied in-depth mass spectrometry-based proteomics, using HiRIEF fractionation and data-dependent acquisition, to thoroughly characterize the disease biology of CLL, followed by data-independent acquisition proteomics to validate previously unknown cancer subgroups. Our approach demonstrates how these proteomics methods can complement each other to combine the benefits of deep and high-throughput proteomic profiling. This is especially relevant for association studies with clinical outcome, which require large cohort sizes to provide sufficient statistical power.

This study was conducted using cryopreserved cells that may vary from fresh, never frozen cells. For future development of clinical proteomics workflows, the impact of sample handling will need to be addressed further in order to ensure robust implementation. However, in this study, we demonstrated that both data-independent acquisition and data-dependent acquisition LC-MS/MS can be used to stratify patients based on proteome subtypes using cryopreserved samples.

Our work comprehensively illustrates that proteomic profiling of cancer cells has the ability to improve the understanding of clinically relevant disease biology beyond transcriptomic and genomic profiling. We linked proteomic and transcriptomic profiles to important recurrent genetic aberrations in CLL. This revealed, for instance, that RNA and protein levels were disconnected for important CLL drivers like *TP53* and *XPO1* mutations, while the biologically meaningful information was primarily contained in the proteome.

We utilized a method to simultaneously detect covariances across multiple omics layers and discovered a proteomic profile of CLL without a corresponding genetic or transcriptomic profile, which was strongly associated with clinical outcome. Proteomics was able to provide a better prognostic split even in the untreated subset of patients. So far, few integrative multi-omics studies have managed to find new associations of proteomic profiles with clinical outcome^21,26,39,40^. Our results demonstrate that this poor prognostic signature is stable across patient cohorts and can be determined by different techniques. These findings highlight that proteomic profiling of cancer can improve molecular stratification of cancer patients.

This biological axis of CLL discovered by proteomics could be identified in approximately 20% of CLL patients. This subgroup was named ASB-CLL and was characterized by a high proliferative capacity and poor overall survival. We found it to be independent of conventional risk factors such as *TP53* aberrations^41^ and IGHV mutation status^5^. These findings illustrate the added value of proteomics for a better understanding of the clinically relevant disease biology of CLL. Our example using CLL as a model disease entity shows that clinical implementation of mass spectrometry-based screening, using the data from this study as a template, can aid not only in identifying patients at particular risk for aggressive disease but also in tailoring treatment and clinical monitoring accordingly. Our cohort was not enriched for high-risk genomic aberrations. Further studies are therefore necessary to investigate how ASB-CLL relates to high-risk genomic features such as *TP53* and how it could be integrated with genomic features to guide clinical treatment.

ASB-CLL was characterized by low abundances of major BcR signaling proteins and high abundances of components of the spliceosome. This directly translated into an altered splicing pattern with increased exon-inclusion and was independent of mutations in *SF3B1*^35^. These results describe that deregulated splicing in CLL occurs as a result of splicing factor upregulation rather than splicing factor mutation. Phosphoproteomic analysis of our data also supported that the decreased levels of BcR signaling proteins was followed by a concurrent decrease in phosphorylation levels of the pathway. Future mechanistic studies are needed to clarify how spliceosomal protein abundance is regulated in this subgroup of CLL and how intracellular signaling pathways are affected by this.

Trisomy 12 and IGHV status explained four of six subgroups with distinct proteomic profiles, suggesting that the interaction of these genetic factors drives the biological differences between these subgroups. While the IGHV mutation status is known to influence the strength of BcR signaling and response to BcR inhibitors^42^, the biology of trisomy 12 is still insufficiently understood. Our results improve the current understanding of trisomy 12 and demonstrate that trisomy 12 significantly increases the abundance of BcR signaling proteins and BcR activity. In accordance with a previous study we showed that treatment-naive M-CLL patients without trisomy 12 had an indolent disease course while M-CLL patients with trisomy 12 had a faster disease progression^43^. The accelerated progression dynamics of trisomy 12 positive M-CLL was not observed in ibrutinib-treated CLL patients, which implies that inhibition of trisomy 12 mediated BcR activation compensated for this disadvantage. Future clinical studies should explore how to best exploit this new insight and whether trisomy 12 M-CLL patients benefit from a BcR inhibitor treatment algorithm designed for U-CLL. These results exemplify the importance of genetic marker combinatorics for gene expression, protein abundance, drug response and disease progression.

Our integrative multi-omics analysis of CLL provides a comprehensive overview of the interplay between genetic alterations, the transcriptome, and the proteome, along with functional consequences for drug response and clinical outcome. The detailed analysis of our dataset improves our understanding of the biological heterogeneity of CLL and provides molecular phenotype-based subtypes that will improve patient stratification and personalized treatments. Through our web application, we provide this comprehensive dataset as a valuable and easily accessible resource to the research community (https://www.dietrichlab.de/CLL_Proteomics/).

## Methods

Our research complies with all relevant ethical regulations. Written consent was obtained from patients according to the declaration of Helsinki. The collection of samples and clinical data was approved by the ethics commission of the medical faculty of the University of Cologne (13-091), the department of hematology Heidelberg (Ethics vote S-686/2018), and the Stockholm Regional Ethics Board (2006/964-31/2 and 99-154).

### Experimental procedures

#### Study design

The purpose of this study was to investigate the relationships between the proteome, transcriptome, and genetic aberrations to clinical parameters (notably TTNT) and drug sensitivity in CLL. The proteome of CLL cells from 68 patient samples was analyzed by HiRIEF-LC–MS/MS, the transcriptome of 59 patients by RNA-sequencing, and mutations of 68 samples by DNA panel sequencing. To minimize the introduction of biases all of these datasets were acquired from the same aliquot of cells. Drug sensitivity was assessed by microscopy-based ex-vivo drug screening and clinical parameters (TTNT) were determined from patient records.

The results obtained from this discovery cohort were validated in five independent CLL cohorts.

#### Discovery cohort: patient samples

Written consent was obtained from patients according to the declaration of Helsinki. Leukemia cells were isolated from blood using Ficoll density gradient centrifugation. Cells were viably frozen and kept on liquid nitrogen until use. 45 CLL patients were untreated at sample collection and 23 had received prior treatment with immuno-chemotherapy (*n* = 17) or with novel drugs (*n* = 6).

#### Discovery cohort: sample preparation for proteomics, RNA, and panel sequencing

Cells were thawed, allowed to recover in RPMI medium (Thermo Fisher Scientific) containing 10% human serum (Sigma Aldrich) for 3 h, and filtered through a 40 µm cell strainer for removal of dead cells from the thawing process. Viability was assessed with Trypan Blue. Only samples with a viability above 90% were included. Tumor cells were collected by Magnetic-activated cell sorting (MACS) using CD19 beads (Miltenyi Biotec). Samples were split into aliquots for proteomics analysis (1 × 10^7^ cells), RNA sequencing (5 × 10^6^–1 × 10^7^ cells), and panel sequencing (5 × 10^6^ cells). Pellets for proteomics analysis were washed twice with PBS and snap frozen in liquid nitrogen. DNA for panel sequencing was extracted using the DNeasy Blood & Tissue Kit (Qiagen). For RNA sequencing RNA was isolated using QIAzol Lysis Reagent (Qiagen), QIAshredder (Qiagen), and the RNeasy Mini Kit (Qiagen).

#### IGHV mutation status analysis

RNA was isolated and cDNA synthesized using high-capacity cDNA Reverse Transcription Kit (Thermo Fisher Scientific). PCR reactions and analyses were performed with minor modifications^44^ (see supplementary methods for additional details).

#### DNA copy-number variants

DNA copy numbers were assessed using Illumina CytoSNP-12 and HumanOmni2.5-8 microarrays and read out using the iScan array scanner. Fluorescence in situ hybridization (FISH) analysis was performed for del(11)(q22.3), del(17)(p13), del(13)(q14), trisomy 12, gain(8)(q24), and gain(14)(q32). Only alterations present and absent in at least three patients were considered for analyses.

#### Discovery cohort: HiRIEF proteomics

Cell pellets were lysed by 4% SDS lysis buffer and prepared for mass spectrometry analysis using a modified version of the SP3 protein clean-up and digestion protocol^45^. Peptides were labeled with TMT10-plex reagent according to the manufacturer’s protocol (Thermo Fisher Scientific) and separated by immobilized pH gradient - isoelectric focusing (IPG-IEF) on 3–10 strips^28^ Extracted peptide fractions from the IPG-IEF were separated using an online 3000 RSLCnano system coupled to a Thermo Fisher Scientific Q Exactive-HF. MSGF+ and Percolator in the Galaxy platform were used to match MS spectra to the Ensembl92 human protein database^46^. For phosphorylation an additional search was performed where phosphorylation was allowed as a variable modification on serine, threonine, and tyrosine. The mass spectrometry proteomics data have been deposited to the ProteomeXchange Consortium via the PRIDE partner repository with the dataset identifier PXD028936.

#### Discovery cohort: panel sequencing

For gene mutation analysis we designed a customized Illumina™ TruSeq Custom Amplicon (TSCA) panel with two independent primer sets for redundant coverage^29^. Mutations in the genes A*TM, BIRC3, EGR2, FBXW7, MYD88, NFKBIE, POT1, TP53, BRAF, NOTCH1, RPS15, SF3B1*, and *XPO1* were covered. Library preparation was performed using TruSeq Custom Amplicon Assay Kit v1.5 and sequenced on an Illumina MiSeq flowcell. For analysis a custom bioinformatics pipeline was used. See supplementary methods for additional details.

#### Discovery cohort: mRNA Sequencing

Stranded mRNA sequencing, using a TruSeq Stranded Total RNA Library Preparation Kit, was performed on a Illumina NextSeq 500. Reads were aligned to GRCh37.75/hg19 using STAR (v2.6.0c;^47^) and counted with htseq-count^48^. Library size normalization, variance stabilizing transformation and differential expression calling were performed using DESeq2 (version 1.28.1)^49^. The data are available through the European Genome-Phenome Archive (EGA) under accession number EGAS00001005746.

#### Discovery cohort: Ex-vivo drug sensitivity screen

Drug response profiles were obtained for 68 leukemia samples and 43 drugs (Selleckchem) in 3 concentrations (Supplementary Data 4). Cells were thawed and seeded in DMEM medium (Thermo Fisher Scientific) containing 10% human serum (Sigma Aldrich), 1% penicillin/streptomycin (Thermo Fisher Scientific) and 1% glutamine (Thermo Fisher Scientific) at 2 × 10^4^ cells/well into a CellCarrier-384 Ultra Microplate (Perkin Elmer). Cells were incubated at 37 °C in a humidified atmosphere and 10% CO_2_ for 3 days.

Cells were stained with 4 µg/ml Hoechst 33342 (Thermo Fisher Scientific). Images were taken using an Opera Phenix High Content Screening System (Perkin Elmer) and processed with Harmony (Perkin Elmer). Cells were segmented and the nuclear area was calculated. Based on a threshold of 23.8 µM^2^ nuclear area cells were classified into alive and dead. The percentage of alive cells was calculated and normalized by dividing through the mean percentage of alive cells across all solvent (DMSO) controls.

#### Validation1_DIA cohort: DIA proteomics

For DIA based proteomics cell pellets were digested and cleaned as described above. Unlabeled peptides from individual samples were separated using an online 3000 RSLCnano system coupled to a Thermo Fisher Scientific Q Exactive-HF. Data-independent acquisition (DIA) was employed using a variable window strategy. Spectronaut was used to analyze the spectral files using the Direct-DIA option and files were searched against the ENSEMBL database (see supplementary methods for additional details). In total 203 samples were analyzed by DIA, 36 from the original discovery cohort and 167 validation samples, 2 validation samples (CLL_DIA_209 and CLL_DIA_165) were excluded from further analysis due to low proteome coverage (<3000 proteins quantitated). The mass spectrometry proteomics data have been deposited to the ProteomeXchange Consortium via the PRIDE partner repository with the dataset identifier PXD024544.

#### Validation2_Eagle cohort

For an additional validation of the existence of ASB-CLL in an external cohort, we took advantage of the cohort of 18 CLL patient samples published by Eagle et al.^24^. Presence of trisomy 12 was estimated by calculating the mean abundance of proteins located on chromosome 12 and defining the 20% of patients with highest abundances as harboring trisomy 12.

#### Validation3_RNA cohort: RNA Sequencing

To validate the splicing signature of ASB-CLL we took advantage of a cohort of 169 CLL patients for who paired-end sequencing, using an Illumina TruSeq RNA sample preparation kit v2, was performed on an Illumina HiSeq2000 with 300 bp insert size^50^. No overlap between the samples of the discovery cohort, the Validation1_DIA cohort or the Validation3_RNA cohort existed. The data is available through the European Genome-Phenome Archive (EGA) under accession number EGAS00001001746.

#### Validation4_untreated cohort

For the evaluation of the effect of the interaction between trisomy 12 and IGHV mutation status in an untreated context, we took advantage of the CLL cohort published by Tissino et al.^51^. All the cases were from a single institution, i.e. the Hematology Unit of the University of Tor Vergata in Rome, and analyzed at the CRO in Aviano for IGHV gene status, FISH categories (del(17p), del(11q), trisomy 12, and del(13q)) and *TP53* mutations. This comprised in total 620 patients. Time to first treatment was used as the clinical endpoint.

#### Validation5_ibrutinib cohort

For the evaluation of the effect of the interaction between trisomy 12 and IGHV mutation status in an ibrutinib-treated context we took advantage of a retrospective study cohort of 463 CLL patients homogeneously treated with ibrutinib at the Ohio State University.

### Statistical analyses

All statistical analyses were performed in R (version 4.0.2). Statistical tests were performed as indicated in the text and figures. Wilcoxon signed-rank tests were always two-sided. Boxplots are defined as follows: center line, median; box limits, upper and lower quartiles; whiskers, 1.5x interquartile range. The code used is available at github^52^.

#### Analysis of differential proteins and mRNA

Differential protein abundance between samples with different genetic alterations was assessed with limma (version 3.44.3)^53^ and DEqMS (version 1.6.0)^54^ (differential proteins defined as adjusted *p* < 0.001 and |log2FC| > 0.5) (github code: “limmaProteomics”^52^). Differential gene expression was performed on the raw count values using DESeq2^49^ (differential genes defined as adjusted *p* < 0.001 and |log2FC|>1.5; github code: “RNASeq”^52^).

For samples with known trisomy 12 and IGHV mutation status (*n*_samples_ = 59), we estimated the proteome-wide (*n*_proteins_ = 7311) relative effect of trisomy 12 in the context of IGHV status by using the following design matrix in DeqMS: ~ IGHV + Trisomy 12+IGHV:Trisomy, and extracted results with respect to the interaction coefficient. Enrichment analysis was performed in the sorted by fold-change gene vector using fgsea^55^ R package and MSigDB KEGG canonical pathways^56^.

For overlapping gene symbols and samples in transcriptomics and proteomics data (*n* = 6310, *m* = 50), we estimated mRNA—protein correlation for each gene i using the first coefficient in the following model:

1 $${{{{{\rm{lm}}}}}}\left({\left[{{{{{{\rm{scaled}}}}}}}_{{{{{{\rm{mRNA}}}}}}}\right]}_{{{{{{\rm{i}}}}}}} \sim \left(\right.{[{{{{{{\rm{scaled}}}}}}}_{{{{{{\rm{protein}}}}}}}]}_{{{{{{\rm{i}}}}}}}+{{{{{\rm{IGHV}}}}}}+{{{{{\rm{Trisomy}}}}}}12+{{{{{\rm{IGHV}}}}}}:{{{{{\rm{Trisomy}}}}}}12\right)$$

Significant correlated pathways were identified by two-sided *t*-tests between the mRNA–protein coefficients of leading edge genes (≥5) versus all genes at a Benjamini–Hochberg FDR level of 0.01.

#### Protein-mRNA correlation

For each protein-mRNA pair in the discovery cohort the Spearman correlation was calculated. Cumulative distribution functions of the correlation coefficients were compared using a two-sided Kolmogorov–Smirnov test (github code: “RNASeq”^52^).

#### Multi-omics factor analysis

The multi-omics factor analysis was performed on genetics, transcriptomics, and proteomics datasets using the MOFA R package (version 1.0.0)^33^. Only the 2000 proteins with the highest variance were used. The MOFA model was calculated 10 times and the model with the highest evidence lower bound (ELBO) was chosen. The convergence threshold was set to 0.01. Only factors that explained at least 1.5% of variance were kept for further analysis (github code: “perfom_MOFA_analysis”^52^).

#### In vivo lymphocyte growth rate

Patients who had lymphocyte counts available for less than four time points between the sample collection date and the time of the next treatment and patients currently in treatment were excluded, leaving 35 patients. Lymphocyte growth rates were calculated by fitting a linear model to the log10 transformed lymphocyte counts versus the period of time.

#### Analysis of outcome

##### Time to next treatment

Patients for which no clinical follow-up data was available were excluded. Time to next treatment (TTNT) was calculated from the date of sample collection to subsequent treatment initiation. Patients without treatment initiation during observation time and patients who died before treatment initiation were censored at the latest follow-up contact. Proportional hazards regression (Cox regression) was used to explore the potential impact of protein abundances on TTNT using the R package survival (version 3.2-3). To draw Kaplan–Meier curves the survminer package (version 0.4.8) was used (github code: “CoxRegr”^52^).

##### Time to first treatment

Patients for which no clinical follow-up data was available were excluded. Time to first treatment was calculated from the date of diagnosis to subsequent treatment initiation. Patients without treatment initiation during observation time and patients who died before treatment initiation were censored at the latest follow-up contact. To draw Kaplan–Meier curves the survminer package (version 0.4.8) was used.

##### Overall survival

Patients for which no clinical follow-up data was available were excluded. Overall survival (OS) was calculated from the date of sample collection to the date of death. Patients who did not die within the observation period were censored at the latest follow-up contact. Proportional hazards regression (Cox regression) was used to explore the potential impact of protein abundances on OS using the R package survival(version 3.2-3). To draw Kaplan–Meier curves the survminer package (version 0.4.8) was used.

TTNT or OS were used as the primary metrics for outcome analysis for groups identified by proteomic or transcriptomic data. For groups stratified from genetic alterations (e.g. IGHV status and Trisomy 12) TTFT or progression free survival (PFS) was used.

#### Dimensionality reduction and consensus clustering

Consensus clustering on proteins or transcripts was performed using the ConsensusClusterPlus package (version 1.52.0)^57^ (github code: “ConsensusClustering”^52^). The optimal number of clusters was determined based on cluster stabilities (Supplementary Data 5). This supported clustering of proteomic data into 4-6 clusters. As the increase from 5 to 6 clusters led to the subdivision of trisomy 12 patients into U-CLL and M-CLL, indicating biological meaningfulness of the clusters, 6 clusters were chosen. mRNA level clustering into less than 5 groups was not justifiable from the relative change in area under the CDF curve (Supplementary Data 6). Clustering into more than 5 subgroups led to splitting off of individual patients. Therefore, 5 clusters were chosen as optimal.

Dimensionality reduction was done by T-distributed stochastic neighbor embedding (t-SNE; Rtsne package, version 0.15), principal component analysis (stats package), and hierarchical clustering (pheatmap package, version 1.0.12) (github code: “DimensionReduction”^52^).

#### Analysis of differential splicing

##### RNA level

For the alternative splicing analysis of the discovery cohort, 59 single-read stranded total RNA-seq samples were processed. First, RNA-seq reads were aligned to the human reference genome (build hs37d5, based on NCBI GRCh37, hg19) using STAR (v2.5.2a) with standard parameters. Aligned RNA-seq data were then subjected to quality control by RNASeQC (v1.1.8) to ensure the integrity of the transcriptome dataset. The same procedure was applied to the Validation3_RNA cohort of 169 paired-end RNA-seq samples.

Our alternative splicing analyses largely rely on rMATS, which we employed to identify exon skipping events, mutually exclusive exons, alternative 5′ and 3′ splice sites, and intron retention in individual CLL patient RNA-seq samples. The rMATS tool was also used to quantify alternative splicing activity by calculating estimates for the percent spliced-in (PSI) values associated with each event in a given sample. The entirety of PSI values for a specific sample will be referred to as the sample’s splicing profile in the following and can be seen as characterizing alternative splicing activity in the corresponding CLL patient.

In the case of the discovery cohort, where samples were independently assigned a proteomics group label, rMATS was additionally used to perform a statistical analysis of differences in alternative splicing patterns between ASB-CLL patients and the remaining samples. Based on the results of such a differential alternative splicing analysis within the skipped exon category, we selected the most interesting events in terms of statistical significance (BH-adjusted *p* value smaller than 1%) and effect size (absolute difference of groupwise mean PSI values larger than 0.1) for further consideration. Additionally, we excluded events with too low a coverage in read counts (average number of raw inclusion counts across all samples not larger than 20). A total of 430 exon skipping events satisfied all three constraints, 427 of which were also detected in the Validation3_RNA cohort. Reading rMATS output into R and selecting events was done with the help of the maser package.

In order to assess the probability that the observed number of significant exon skipping events was purely due to chance, we performed a permutation test. Specifically, we uniformly drew nine samples from the total list of 59 CLL samples in the discovery cohort without replacement and labeled them as “ASB-CLL”. The remaining 50 samples were labeled as “other”. We then ran rMATS on these randomly labeled samples using the same parameters as in the original analysis and again determined the number *N*_e_ of significant exon skipping events. This process was repeated for multiple such label permutations, thus generating an estimate for the null distribution of *N*_e_. The tested null hypothesis is that there is no differential splicing between ASB-CLL and the other proteomics groups.

Apart from the supervised selection of exon skipping events described further above, we performed unsupervised exploratory analysis for each alternative splicing event category in the discovery cohort. We did so by using the 1000 most variable events across all patients in each category. For each patient sample, we then calculated the mean PSI value based on those 1000 events. In order to quantify whether the mean PSI values from ASB-CLL samples tend to be different from those in all other PGs, we performed a two-sided Wilcoxon rank-sum test. The outcome of that test gave us an idea about overall alternative splicing differences in the given category.

In order to capture the characteristic splicing profile of ASB-CLL samples, we used the caret package to train a partial least squares discriminant analysis (PLS-DA) model on the discovery cohort using the PSI values from the 427 selected exon skipping events as predictors. All predictors were centered and scaled prior to training. The PLS-DA classifier has one tuning parameter, namely the number *N*_c_ of components in the dimensionally reduced space. We determined the optimal value for *N*_c_ via leave-one-out cross validation using the area under the ROC curve as the measure of predictive performance to be optimized.

##### Peptide level

Peptide sequences were stripped of modifications and merged by the median followed by mapping to genomic coordinates using the *Splicevista.py* function^58^ and assigned to Ensembl exon IDs (GRCh38, v92). Each peptide was assigned to exon(s)—in case of splice-junction—based on the initial master protein assignment, but later distributed to multiple proteins supported by the exon(s). Splice-junction-covering peptides were treated as single instances to accommodate the unique quantitative profile deriving from both putative assignments. As such, exons here denote unique coding units, but we follow the exon term for simplification.

Differential splicing was investigated using the limma diffsplice *R* function^53^. Specifically, for exons quantified in more than two TMT sets (18 samples), we estimated log fold changes of ‘this-cluster-vs-the-rest’ contrasts via moderated t-tests of ‘this-exon-vs-the-rest’ comparisons. Exon-level significance was determined by *t*-test (FDR corrected *p* value < 0.01 and |log2FC| > 0.5). Enriched groups for significant hits were identified using one-sided Fisher’s exact tests as described above (github code: “CLL_exon_centric_diffSpiced_diffQuant”^52^).

#### Gene ontology and KEGG gene set enrichment analyses

Enrichment analysis was performed against KEGG gene sets^59^ using GSEA (version 4.0.3)^56^. For GO term enrichment^60^ of proteins loaded onto latent factor 9 the MOFA^33^ internal function *runEnrichmentAnalysis* was used. The required GO terms were downloaded from MSigDB^61^ (v7.0) and the FDR cut-off was set to >5%.

#### Protein–protein correlation

For the protein core complex analysis protein–protein Pearson correlations were calculated for CORUM complex members and converted into a pairwise interaction matrix^15^.

For the generation of the protein correlation network proteins with a high standard deviation were selected and pairwise Pearson correlations were calculated (github code: “ppi_network”^52^). The resulting network was visualized in Gephi 0.9.2 and nodes were filtered using a kcore setting of 3. Modularity clustering of the nodes was carried out with a resolution of 0.8. Annotation of the modularity clusters was done by first extracting all proteins belonging to a cluster. Next, any protein in the full overlap dataset (*n* = 7313) with a Pearson correlation above 0.7 to any of the cluster members was included in the target gene set for that cluster. Enrichment was carried out against the MSigDB and the R packages msigdbr and ClusterProfiler were used to calculate enrichments (github code: “enrichment_of_network-msigdb”^52^). See supplementary methods for additional details.

#### Validation of ASB-CLL using DIA based proteomics

To validate the existence of ASB-CLL we analyzed 167 new patient samples from Sweden and Germany using DIA based proteomics (Validation1_DIA cohort) as well as 36 samples from the original discovery cohort. Four samples were excluded after quality control assessment due to either poor correlation to the in-depth data (2 samples, discovery cohort) or a low number of identifications (2 samples, Validation1_DIA). We trained a *k*-TSP based classifier on the relative MS2-level quantifications of all identified proteins from the KEGG “Spliceosome” and “B-cell receptor signaling” pathways which were identified by at least 3 spectral matches in the training set. After optimization 8 *k*-TSP pairs were chosen. 36 of the original 68 samples analyzed by HiRIEF were run on DIA and samples with a correlation between the DIA and HiRIEF data of >0.4 were used as a training set. Repeated Monte carlo cross validation was used to optimize both the number of pairs and to identify the pairs which best separated ASB-CLL.

For additional validation of ASB-CLL the published proteomics dataset by Eagle et al.^24^ was used (Validation2_Eagle cohort). The *k*-TSP classifier trained above was used on the Eagle et al. dataset. One TSP-pair had to be excluded due to lack of overlap and the classification threshold was lowered by 1 as a consequence. Mean protein abundances for the genes in the KEGG gene sets “Spliceosome”, “B-cell receptor signaling”, “proteasome”, and “Valine, leucine and isoleucine degradation” were calculated and compared between the ASB-CLL cluster and all other patients.

#### Validation of Tris12M-PG, Tris12U-PG, M-PG, and U-PG

For the confirmation of the clinical relevance of Tris12M-PG, Tris12U-PG, M-PG, and U-PG multiple validation cohorts were used. Patients from the Validation1_DIA cohort (*n* = 165), the Validation4_untreated cohort (*n* = 620), only comprising untreated CLL samples, and the Validation5_ibrutinib cohort (*n* = 463), only comprising ibrutinib-treated samples, were split into four groups based on IGHV status and trisomy 12. This was possible because Tris12M-PG, Tris12U-PG, M-PG, and U-PG strongly associated with these two genetic alterations. To assess the native disease progression differences in time to first treatment between the four groups were assessed in the Validation1_DIA and the Validation4_untreated cohorts using Kaplan–Meier curves and log-rank test. To test differences between the groups in the context of treatment with ibrutinib time to progression was evaluated in the Validation5_ibrutinib cohort using Kaplan–Meier curves and log-rank test.

### Reporting summary

Further information on research design is available in the Nature Research Reporting Summary linked to this article.

## Supplementary information

Supplementary Info

Reporting Summary

Description of Additional Supplementary Files

Supplementary data 1

Supplementary data 2

Supplementary data 3

Supplementary data 4

Supplementary data 5

Supplementary data 6

## Data Availability

The mass spectrometry proteomics data have been deposited to the ProteomeXchange Consortium via the PRIDE partner repository with the dataset identifiers PXD028936 (Discovery set) and PXD024544 (DIA Validation set). The Eagle et al. dataset is available as supplementary information alongside the original article^24^. The RNAseq data for the discovery cohort is available through the European Genome-Phenome Archive through accession number EGAS00001005746 and for the Validation3_RNA cohort through accession number EGAS00001001746. As the RNA Sequencing data has the potential to reveal patient-sensitive information the data is under restricted access. Access will be granted within one week by Sascha Dietrich (sascha.dietrich@embl.de) to non-profit studies which remove patient-sensitive information. The data can be easily explored through our web application: https://www.dietrichlab.de/CLL_Proteomics/. Source data are provided with this paper.

## Source data

Source data file

## Code availability

The code not accessing patient-sensitive information has been deposited on github: https://github.com/DietrichLab/Proteogenomics_and_drug_response_CLL^52^.
