# Supplementary material for: Proteogenomics refines the molecular classification of chronic lymphocytic leukemia
Source: Nat Commun. 2022 Oct 20;13:6226. doi: 10.1038/s41467-022-33385-8 (PMC9584885; doi:10.1038/s41467-022-33385-8)
Supplement: Supplementary file 1 — Supplementary Info [file 41467_2022_33385_MOESM1_ESM.pdf]

Supplementary Information

Proteogenomics refines the molecular classification of chronic lymphocytic leukemia

Sophie A. Herbst*[1-5], Mattias Vesterlund*[6], Alexander J. Helmboldt[2], Rozbeh Jafari[6], Ioannis Siavelis[6], Matthias Stahl[6], Eva C. Schitter[1], Nora Liebers[1-4], Berit J. Brinkmann[1-3,5,7], Felix Czernilofsky[1], Tobias Roider[1-3], Peter-Martin Bruch[1-3], Murat Iskar[8], Adam Kittai[9], Ying Huang[9], Junyan Lu[2,3], Sarah Richter[1], Georgios Mermelekas[6], Husen Muhammad Umer[6], Mareike Knoll[1], Carolin Kolb[1], Angela Lenze[1], Xiaofang Cao[6], Cecilia Österholm[10], Linus Wahnschaffe[11], Carmen Herling[11], Sebastian Scheinost[4], Matthias Ganzinger[12], Larry Mansouri[10], Katharina Kriegsmann[1], Mark Kriegsmann[13], Simon Anders[14], Marc Zapatka[8], Giovanni Del Poeta[15], Antonella Zucchetto[16], Riccardo Bomben[16], Valter Gattei[16], Peter Dreger[1], Jennifer Woyach[9], Marco Herling[11], Carsten Müller-Tidow[1-3], Richard Rosenquist[10,17], Stephan Stilgenbauer[18], Thorsten Zenz[4,19], Wolfgang Huber[2,3], Eugen Tausch[18], Janne Lehtiö[§6] and Sascha Dietrich[§1-4]

* These authors contributed equally

§ These authors jointly supervised this work

Affiliations:

1 Department of Medicine V, Hematology, Oncology and Rheumatology, University of Heidelberg, Heidelberg, Germany

2 European Molecular Biology Laboratory (EMBL), Heidelberg, Germany

3 Molecular Medicine Partnership Unit (MMPU), Heidelberg, Germany

4 Department of Translational Medical Oncology, National Center for Tumor Diseases (NCT) Heidelberg and German Cancer Research Center (DKFZ), Heidelberg, Germany

5 Faculty of Biosciences, University of Heidelberg, Heidelberg, Germany

6 Department of Oncology-Pathology, Karolinska Institute and Science for Life Laboratory, Stockholm, Sweden.

7 Clinical Cooperation Unit Molecular Hematology/Oncology, German Cancer Research Center (DKFZ)

8 Division of Molecular Genetics, German Cancer Research Center (DKFZ), Heidelberg, Germany

9 Department of Internal Medicine, Division of Hematology, The Ohio State University, USA

10 Department of Molecular Medicine and Surgery, Karolinska Institutet, Stockholm, Sweden

11 Department I of Internal Medicine, Center for Integrated Oncology Aachen-Bonn-Cologne-Duesseldorf (CIO ABCD), Excellence Cluster for Cellular Stress Response and Aging-Associated Diseases (CECAD), Center for Molecular Medicine Cologne (CMMC), University of Cologne, Cologne, Germany

12 Institute of Medical Biometry and Informatics, Heidelberg University, Heidelberg, Germany

13 Institute of Pathology, University of Heidelberg, Heidelberg, Germany

14 Center for Molecular Biology of the University of Heidelberg (ZMBH), Heidelberg, Germany

15 Division of Hematology, University of Tor Vergata, Rome, Italy

16 Clinical and Experimental Onco-Hematology Unit, Centro di Riferimento Oncologico di Aviano (CRO) IRCCS, Italy

17 Clinical Genetics, Karolinska University Laboratory, Karolinska University Hospital, Stockholm, Sweden

18 Department of Internal Medicine III, University of Ulm, Ulm, Germany

19 Department of Medical Oncology and Hematology, University Hospital Zürich, Zürich, Switzerland

Supplementary tables

	Discovery	Validation1_ DIA	Validation2_ Eagle	Validation3_ RNA	Validation4_ untreated	Validation5_ ibrutinib
n	68 (*100*)	165 *(100)*	18 *(100)*	169 *(100)*	620 *(100)*	463 *(100)*
median follow-up in months	OS: 19, TTNT: 19	OS: 64, TTNT: 27	-	OS: 46, TTNT: 37	TTFT: 83	PFS: 71
IGHV mutated	33 *(52)*	106 (*66)*	9 *(50)*	93 *(56)*	383 *(62)*	118 *(25)*
Trisomy 12	15 *(22)*	20 *(13)*	-	18 *(11)*	94 *(15)*	100 *(22)*
del17p	11 *(16)*	5 *(3)*	-	13 *(8)*	56 *(9)*	-
TP53 mutated	11 *(16)*	14 *(9)*	-	23 *(14)*	80 *(14)*	-
Female	29 *(43)*	59 *(36)*	9 *(50)*	61 *(36)*	258 *(42)*	-
Treatment at sample collection						
untreated	45 *(66)*	111 *(70)*	13 *(72)*	85 *(54)*	620 *(100)*	0 *(0)*
treated	23 (34*)*	47 *(30)*	5 *(28)*	72 *(46)*	0 *(0)*	463 *(100)*
Treatment after sample collection & within observation period						
untreated	30 *(44)*	37 *(22)*	-	99 *(59)*	-	-
treated	38 *(56)*	40 *(24)*	-	70 *(41)*	-	-
not available	0 *(0)*	88 *(53)*	18 *(100)*	0 *(0)*	620 *(100)*	463 *(100)*

Supplementary table 1: Characteristics of the patients in the different cohorts used in this study. Number of patients with a specific characteristic are shown. The percentages in brackets represent the number of patients with this specific feature out of all patients for which this information was available. Validation2_Eagle is the dataset published by Eagle et al. in 2015 [1]**.** The type of treatment regiments the patients had received at any time between diagnosis and sample collection was available to us for the discovery cohort for which 17 patients had been treated with chemo-immunotherapy, while 6 patients had received treatment with novel agents (rituximab + idelalisib or ibrutinib). Median time between last pretreatment and sample collection was 17 months, with 33 % of patients having received prior treatment within the last year and 79 % of patients within the last three years. Specific treatment information was not available for Validation1_DIA, Validation2_Eagle and Validation3_RNA. Validation4_untreated had not received prior treatment. The Validation5_ibrutinib cohort had uniformly been treated with ibrutinib.

**Diagnosis to sample collection**	**< 1 year**	**1-2 years**	**2-5 years**	**5 year**
Discovery cohort				
untreated	3 *(7)*	0 *(0)*	0 *(0)*	42 *(93)*
prior therapy	1 *(4)*	1 *(4)*	6 *(26)*	15 *(65)*
Validation1_DIA				
untreated	4 *(4)*	0 *(0)*	0 *(0)*	106 *(96)*
prior therapy	0 *(0)*	1 *(2)*	3 *(6)*	43 *(91)*
Validation3_RNA				
untreated	1 *(1)*	0 *(0)*	0 *(0)*	82 *(99)*
prior therapy	0 *(0)*	0 *(0)*	0 *(0)*	72 *(100)*

Supplementary table 2: Time from diagnosis to sample collection for the different cohorts used in this study. Number of patients are shown. Percentages are indicated in brackets and are in relation to all patients for which this information was available. Sampling was performed at the time of diagnosis of all patients in Validation4_untreated cohort. For Validation5_Ibrutinib this information was not available.

Supplementary figures

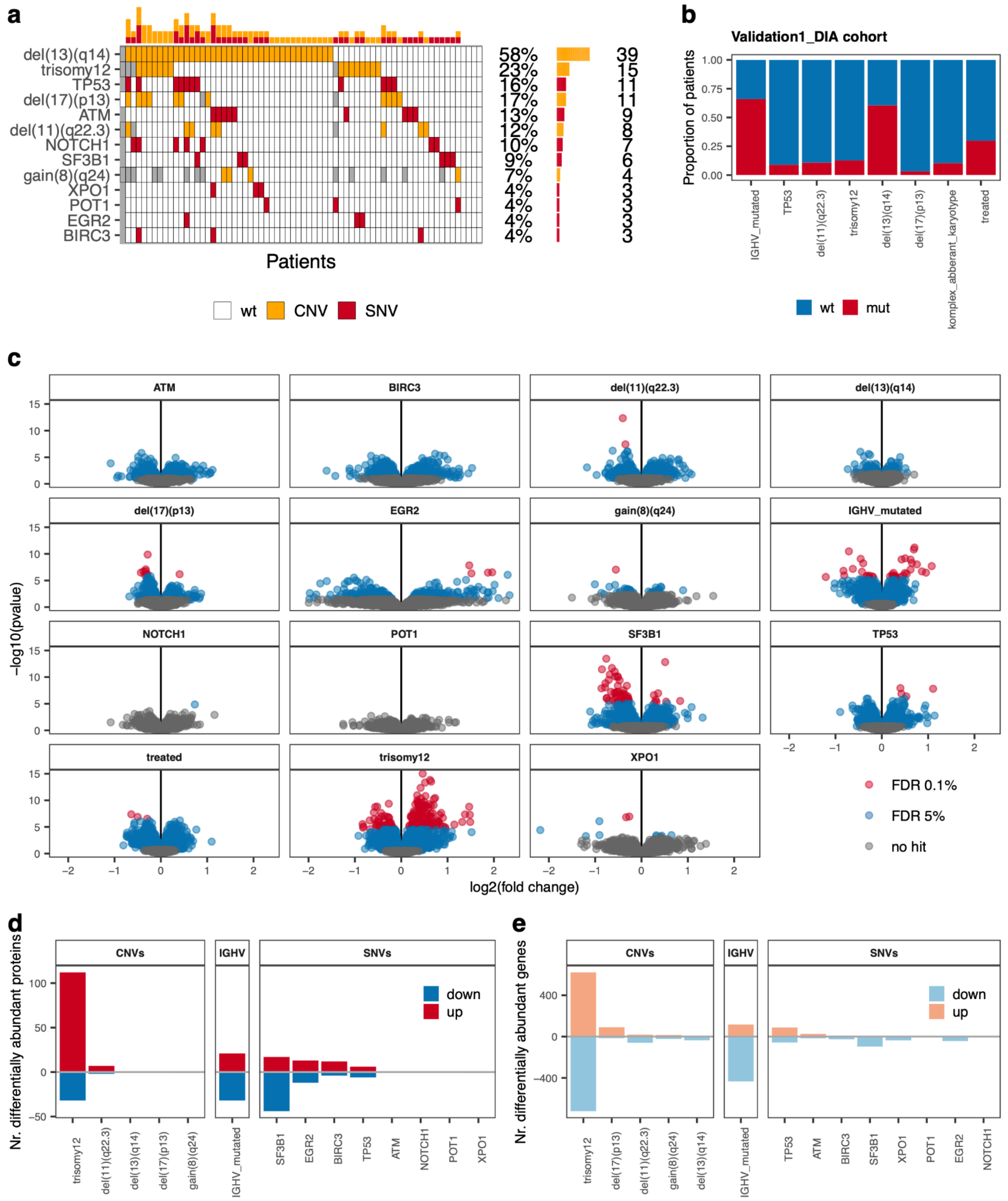

Supplementary figure 1: a, Overview of the single nucleotide (red, SNV) and copy number variants (yellow, CNV) in the discovery cohort of 68 CLL patients. Grey areas indicate missing values. **b,** Distributions of IGHV, treatment status, the copy number alterations trisomy 12, del(11)(q22.3), del(13)(q14) and del(17)(p13), and the presence of *TP53* mutations in the 165 CLL patients of the Validation1_DIA cohort. **c,** Analysis of differential protein abundances for recurrent SNVs, CNVs, IGHV and treatment status using limma. FDR rates of 0.1 % and 5 % are color-coded. **d,** Number of significantly differentially

abundant proteins (FDR 5%; |log2FC| >0.5) and **e,** differentially expressed genes (FDR 5%; |log2FC| >1.5) in relation to recurrent genetic alterations; red/positive numbers =upregulated, blue/negative numbers =downregulated. Source data are provided as a Source Data file.

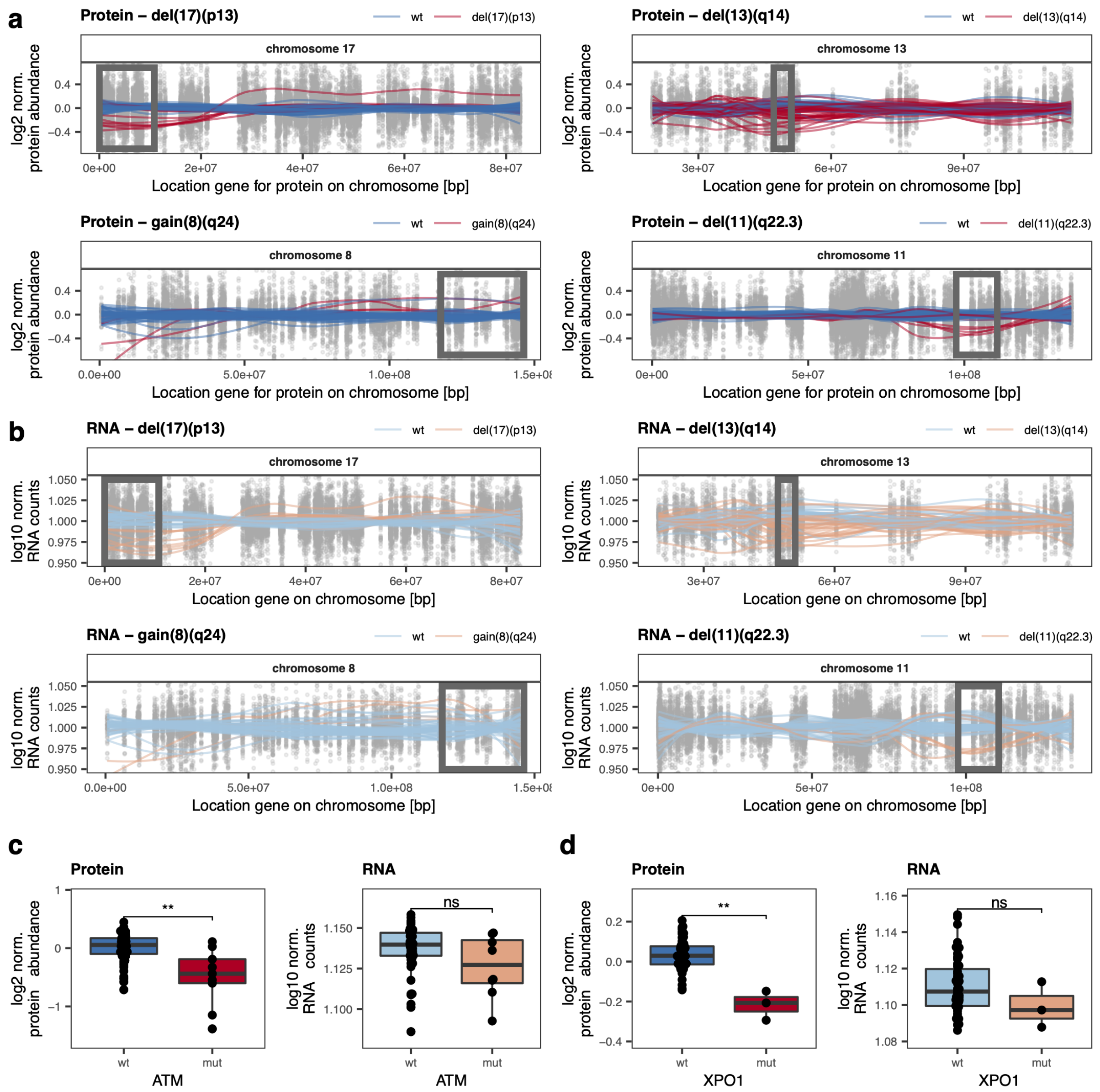

Supplementary figure 2: a, Effect of different copy-number variations on protein abundances. Normalized protein abundance for the chromosomes affected by the alterations are shown. Points represent individual values for protein - patient pairs. Lines are locally weighted scatterplot smoothed values for individual patients with (red) or without (blue) the alteration. The box is the region affected by the alteration. Regions with altered copy number tended to have altered protein abundances. **b,** Effect of different copy-number variations on gene expression. Normalized gene expression levels for the chromosomes affected by the alterations are shown. Points represent individual values for gene - patient pairs. Lines are locally weighted scatterplot smoothed values for individual patients with (red) or without (blue) the alteration. The box is the region affected by the alteration. Regions with altered copy number tended to have altered transcript abundances. **c,** ATM protein (** p =0.001) and transcript levels (not significant p =0.17) in *ATM* mutated (mut, n = 9 Protein or n = 8 RNA biologically independent patient samples) and wild-type (wt, n = 58 Protein or n = 50 RNA biologically independent patient samples) CLL samples; two-sided Wilcoxon signed-rank test. **d,** XPO1 protein (** p =0.004) and transcript levels (not significant p =0.18) in *XPO1* mutated (mut, n = 3 Protein or n = 3 RNA biologically independent patient samples) and wild-type (wt, n = 64 Protein or n = 55 RNA biologically independent patient samples) CLL

samples; two-sided Wilcoxon signed-rank test. All boxplots are represented as first and third quartiles with a median in the center. Whiskers are defined as 1.5 times the interquartile range. Source data are provided as a Source Data file.

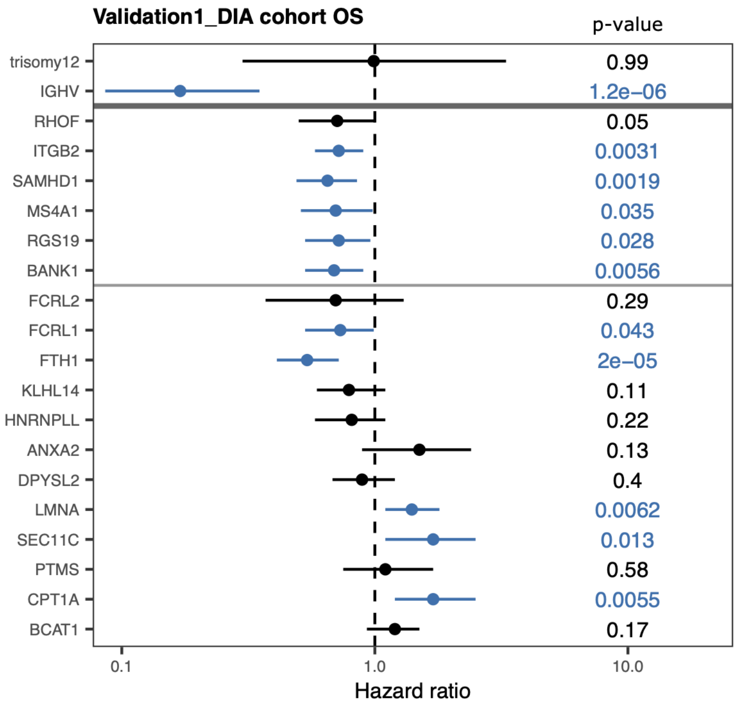

Supplementary figure 3: Hazard ratios from Cox regression for overall survival (OS) with genes and proteins in Validation1_DIA dataset with strong weights for LF1 and LF2 in the discovery cohort. P-values (Wald test) are shown on the right. Significant associations ($p < 0.05$) are colored in blue. Mean and 95 % confidence intervals are shown. Out of the proteins with strongest weights on LF9, none were detected in the DIA dataset. n = 158 biologically independent patient samples. Univariate cox proportional hazards regression model. Wald test was used to calculate p-values. Source data are provided as a Source Data file.

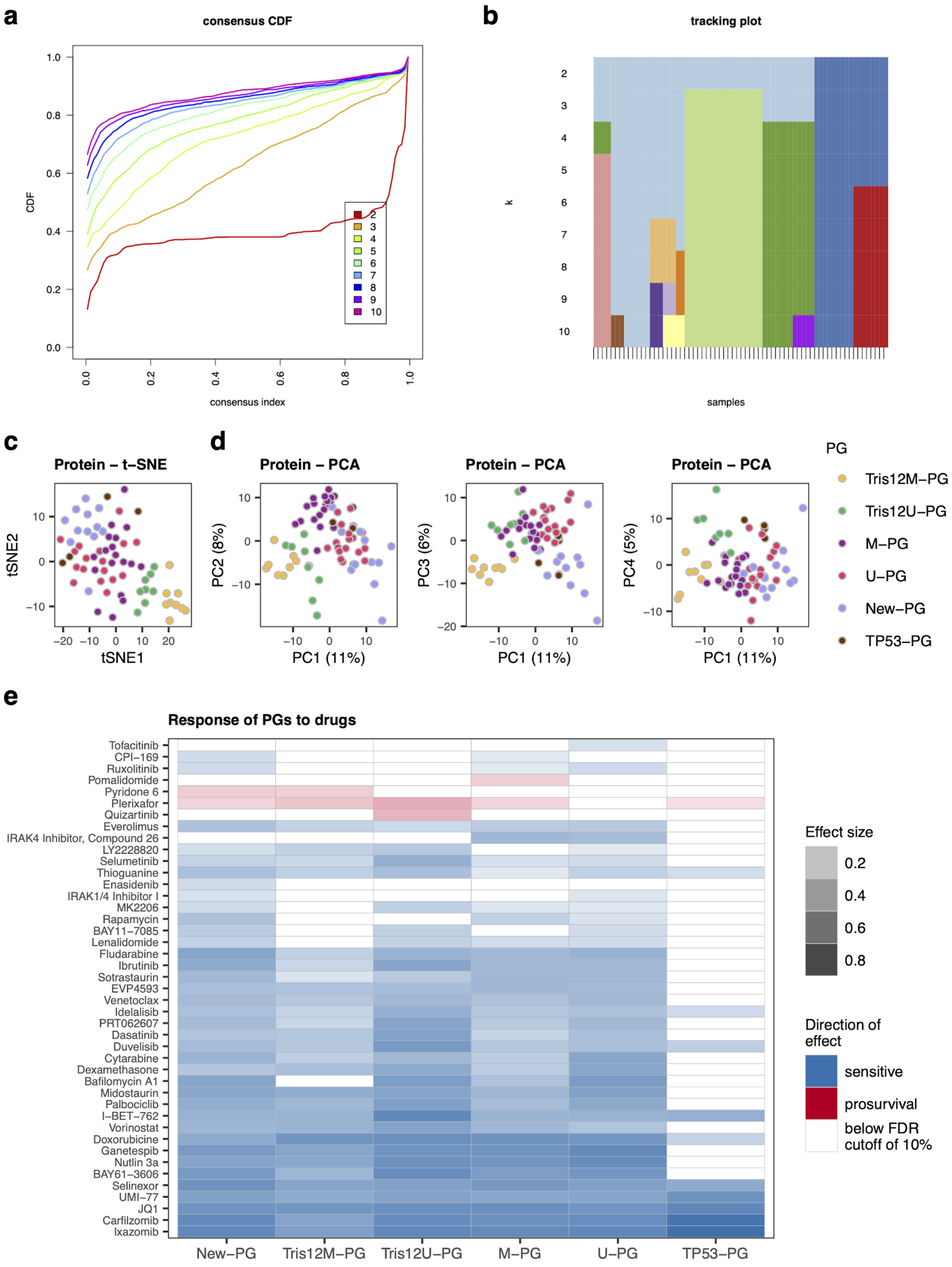

Supplementary figure 4: **a,** Cumulative distribution function as produced by the ConsensusClusterPlus package on the proteomics dataset for a number of up to ten clusters. **b,** Tracking plot of clusters as produced by the ConsensusClusterPlus package on the proteomics dataset for a number of up to ten clusters. **c,** t-SNE of proteomics data color coded by Proteomics Groups (PG). **d,** Principal component analysis of proteomics data color

coded by PG. **e,** Responses of Proteomics Groups (PG) to the individual drugs tested in the *ex-vivo* drug sensitivity screen. Significance of effects was tested by two sided t-tests against the viability of cells in the control condition. Source data are provided as a Source Data file.

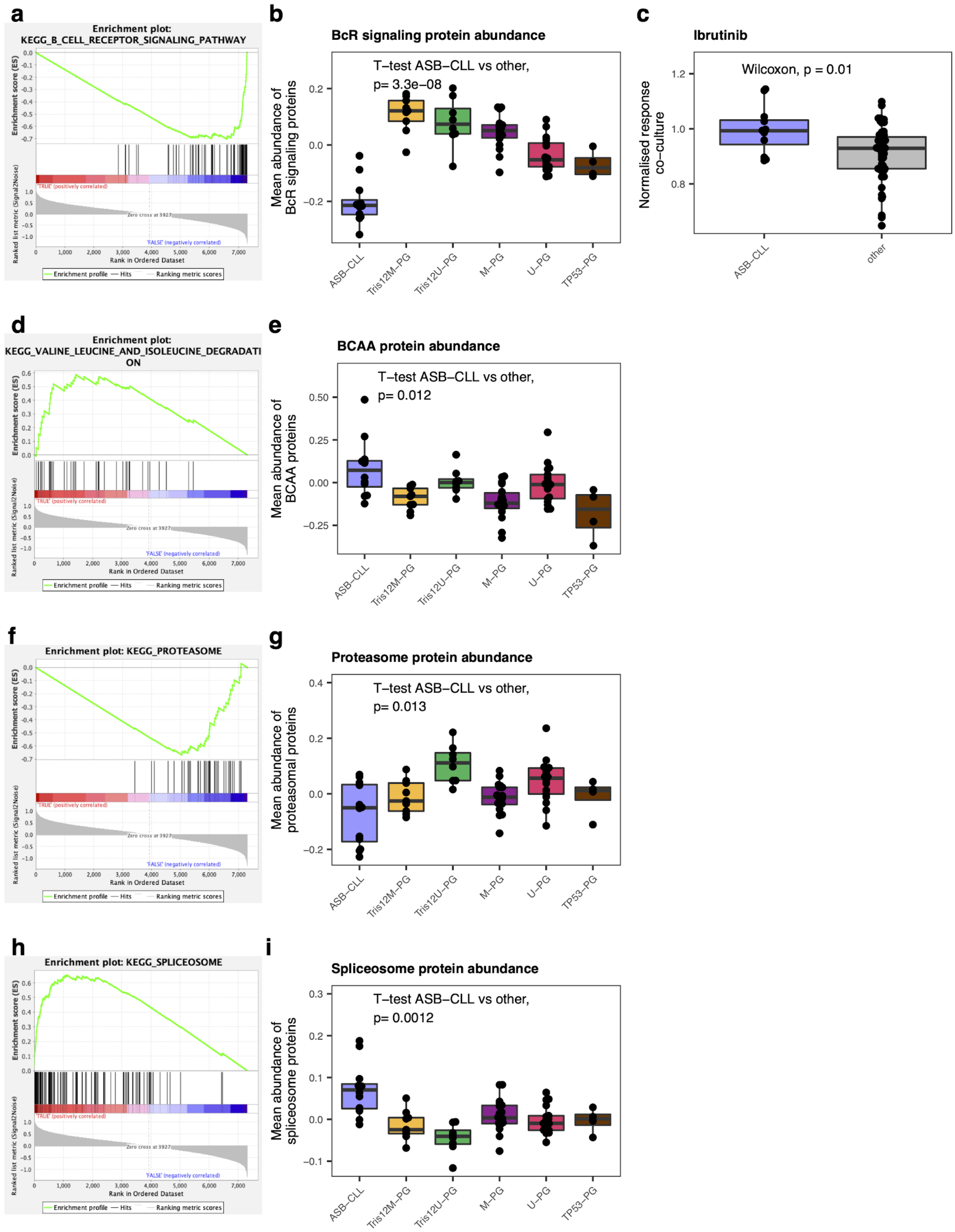

Supplementary figure 5: a, Enrichment plot for the KEGG pathway "B-cell receptor signaling" for differentially abundant proteins in ASB-CLL. **b,** Mean protein abundance of proteins in KEGG pathway "B-cell receptor signaling" across PGs. The comparison of 12 biologically independent ASB-CLL samples with the other 56 biologically independent PG samples is shown. **c,** Percentages, normalized to solvent control, of alive cells CLL samples of the discovery cohort in co-culture with the human bone marrow stroma cell line HS-5,

treated *ex-vivo* with ibrutinib (40 nM). The comparison of 12 biologically independent ASB-CLL samples with the other 56 biologically independent PGs samples is shown; two-sided Wilcoxon signed-rank test. **d,** Enrichment plot for the KEGG pathway “Valine, leucine and isoleucine degradation” for differentially abundant proteins in ASB-CLL. **e,** Mean protein abundance of proteins in KEGG pathway “Valine, leucine and isoleucine degradation” (here termed branched chain amino acid (BCAA) degradation) across PGs. The comparison of 12 biologically independent ASB-CLL samples with the other 56 biologically independent PG samples is shown. **f,** Enrichment plot for the KEGG pathway “Proteasome” for differentially abundant proteins in ASB-CLL. **g,** Mean protein abundance of proteins in KEGG pathway “Proteasome” across PGs. The comparison of 12 biologically independent ASB-CLL samples with the other 56 biologically independent PG samples is shown. **h,** Enrichment plot for the KEGG pathway “Spliceosome” for differentially abundant proteins in ASB-CLL. **i,** Mean protein abundance of proteins in KEGG pathway “Spliceosome” across PGs. The comparison of 12 biologically independent ASB-CLL samples with the other 56 biologically independent PG samples is shown. All significance tests are two-sided and all boxplots are represented as first and third quartiles with a median in the center. Whiskers are defined as 1.5 times the interquartile range (applies to **b, c, e, g** and **i).** Source data are provided as a Source Data file.

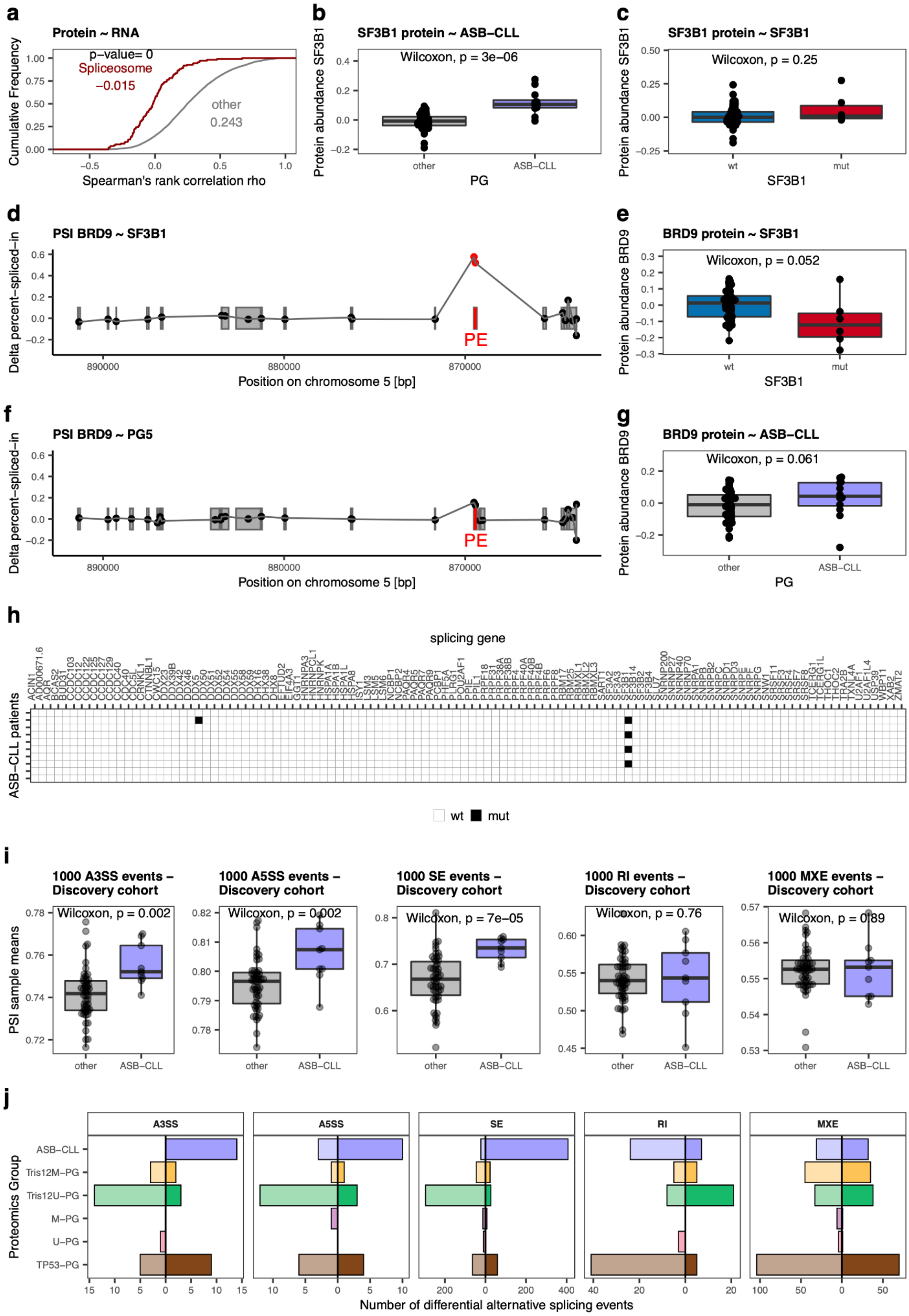

Supplementary figure 6: a, Cumulative density distribution of protein-mRNA Spearman's rank correlations for KEGG components of the spliceosome (red) in comparison to all other

proteins (gray). A two-sided Kolmogorov-Smirnov test was used to determine the p-value. **b,** SF3B1 log2 protein abundances in ASB-CLL (n = 12) vs. all other groups (n = 56). **c,** SF3B1 log2 protein abundances in *SF3B1* mutated (mut, n = 6) vs. wild-type (wt, n = 61) samples. SF3B1 protein levels were independent of *SF3B1* mutations. **d,** *SF3B1* mutated CLL showed an increase in the percent-spliced-in (PSI) value of the poison exon (PE) in BRD9[2]. **e,** BRD9 log2 protein abundances in *SF3B1* mutated (mut, n = 6) vs. wild-type (wt, n = 61) CLL. **f,** ASB-CLL did not show altered PSI value of the poison exon in BRD9. **g,** BRD9 log2 protein abundances in ASB-CLL (n = 12) vs. all other groups (n = 56). **h,** Mutations in genes relevant for splicing in ten ASB-CLL patients, as detected by whole exon sequencing. **i,** Mean PSI value per patient calculated from the 1000 most variable 3' alternative splice site (A3SS), 5' alternative splice site (A5SS), skipped exon (SE), retained introns (RI), and mutually exclusive exon (MXE) events across all patients of the discovery cohort. ASB-CLL patients (n = 8) are compared to non-ASB-CLL patients (n = 50). Comparisons using a two-sided Wilcoxon signed rank test. Boxplots are represented as first and third quartiles with a median in the center. Whiskers are defined as 1.5 times the interquartile range (applies to **b, c, e, g** and **i). j,** Number of differential alternative splicing events in a comparison between the specified proteomics group and all other groups for the same event types as mentioned in panel i (n = 58). Darker (lighter) shades correspond to events for which the mean PSI value in the named group is larger (smaller) than that of all other groups. Source data are provided as a Source Data file.

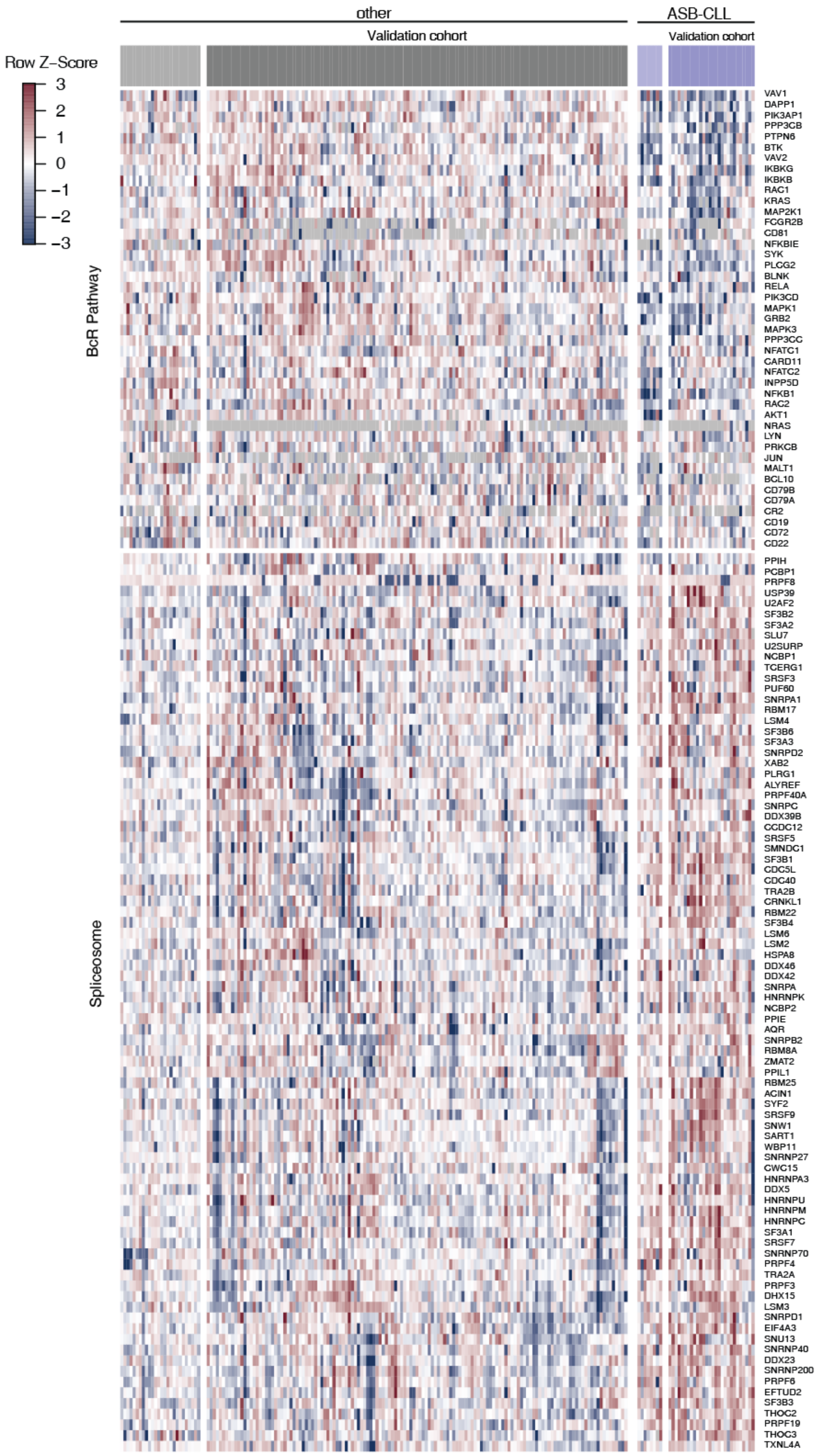

Supplementary figure 7: Heatmap of relative protein abundances for proteins detected by DIA in the BcR and spliceosome pathways (KEGG). Related to figure 6. Patients are

grouped according to predicted membership in the ASB-CLL group: light grey, left = discovery cohort, not ASB–CLL; dark grey = validation cohort, predicted not ASB-CLL; light purple = discovery cohort, ASB-CLL; dark purple = validation cohort, predicted ASB-CLL. Source data are provided as a Source Data file.

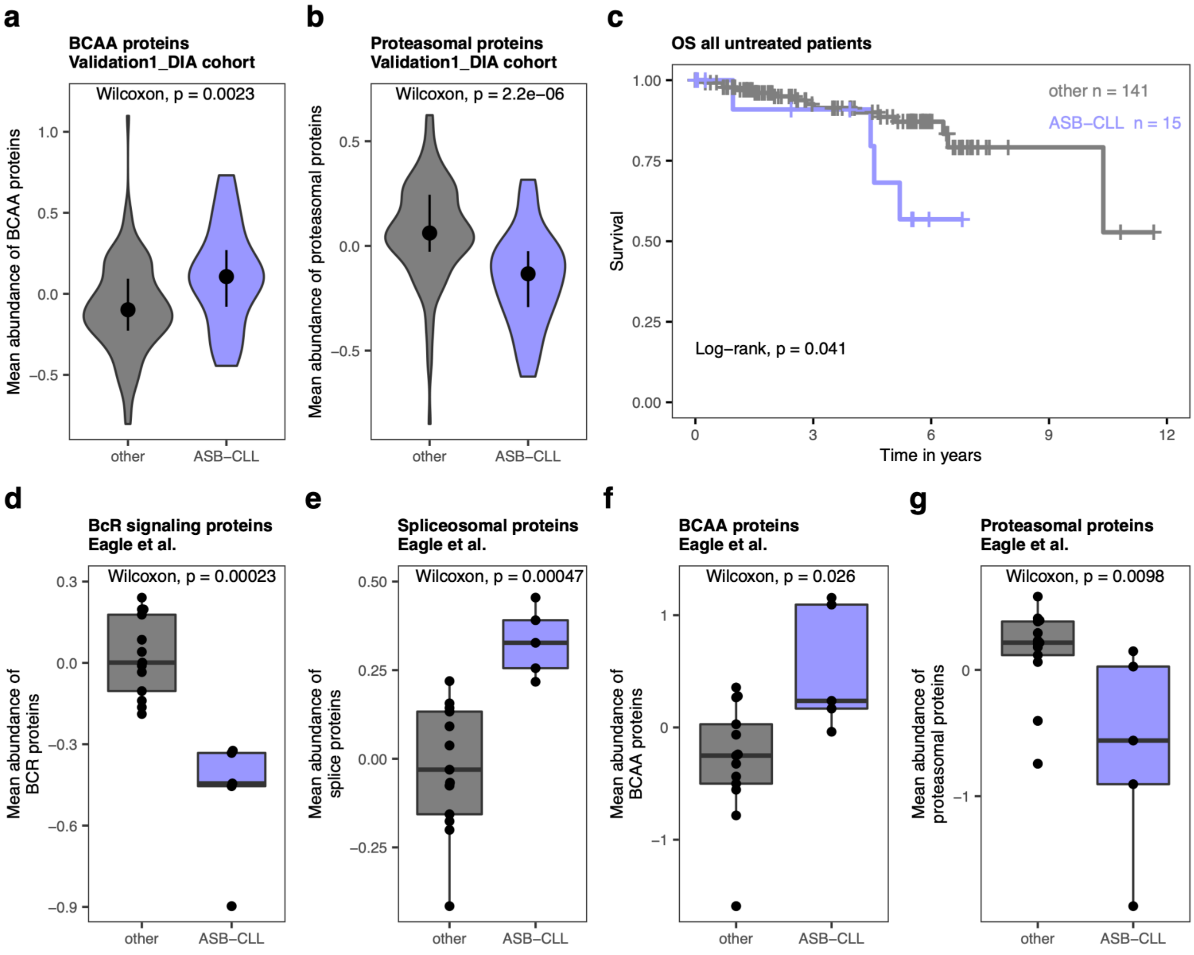

Supplementary figure 8: a and **b**, Violin plots of branched chain amino acid (BCAA) protein abundances (**a**) and proteasomal protein abundances (**b**) comparing the subgroup identified as ASB-CLL in the Validation1_DIA dataset (n=28) to all other patients (n=134). **c,** Overall survival (OS) of untreated patients in the discovery and Validation1_DIA cohorts, divided into ASB-CLL and all other patients. **d-g**, Boxplots of B cell receptor (BcR) signaling protein (**d**), spliceosomal protein (**e**), BCAA protein (**f**) and proteasomal protein (**g**) abundances in the subgroup identified as ASB-CLL (n = 5 patient samples) in the Validation2_Eagle cohort, in comparison to all other patients (n = 13 patient samples). Comparison using a two-sided Wilcoxon signed rank test. Boxplots are represented as first and third quartiles with a median in the center. Whiskers are defined as 1.5 times the interquartile range (**d-g**). Source data are provided as a Source Data file.

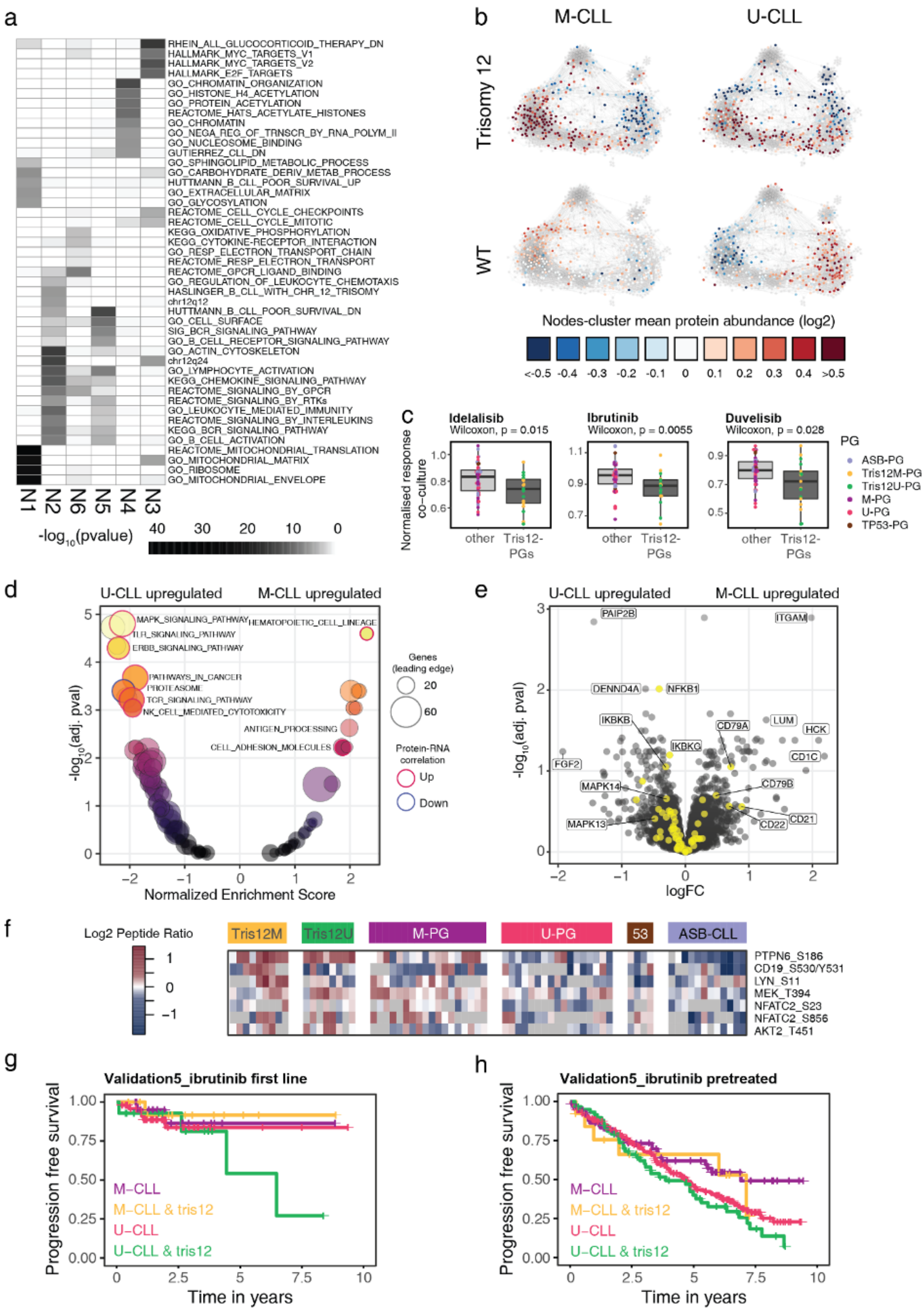

Supplementary figure 9: a, Heatmap of p-values for selected enrichment terms for the modularity defined clusters N1-N6 in Fig. 7c. **b**, Visualization of the log2 mean, relative protein levels from the Validation1_DIA dataset for patients grouped by IGHV status and

trisomy 12 status. Proteins not found in the DIA-dataset are colored grey. (M-CLL, tris12+ n= 14; M-CLL, WT, n= 89; U-CLL, tris12+, n= 6; U-CLL, WT, n= 46), **c,** Percentages, normalized to solvent control, of alive cells CLL samples of the discovery cohort in co-culture with the human bone marrow stroma cell line HS-5, treated *ex-vivo* with idelalisib (9 µM), ibrutinib (40 nM) or duvelisib (4.5 µM). 17 biologically independent trisomy12 patient samples (Tris12M-PG, n =9; Tris12U-PG, n=8) were compared to 51 non-trisomy12 biologically independent patient samples (M-PG, n=18; U-PG, n=17, ASB-CLL, n=12; TP53-PG, n=4) using a two-sided Wilcoxon signed rank test. Boxplots are represented as first and third quartiles with a median in the center. Whiskers are defined as 1.5 times the interquartile range. **d,** GSEA analysis results using the KEGG database for protein level differences between U-CLL and M-CLL in the context of trisomy 12. Data was adjusted for differences between U-CLL and M-CLL in cases with disomy 12 (WT). Leading edge genesets with significantly different protein-mRNA correlations are highlighted (red circle = significantly higher correlation, blue circle = Significantly lower correlation) **e,** Volcano plot of DeqMS analysis results of protein level differences between U-CLL and M-CLL in the context of trisomy 12. Data was adjusted for differences between U-CLL and M-CLL in cases with disomy 12 (WT). Genes from the B cell receptor signaling pathway are highlighted in yellow. **f**, Heatmap of log2 peptide abundances for selected phosphorylated BcR peptides. Patients were grouped according to PG. **g,** Time to progression of patients uniformly treated with ibrutinib as first line treatment (Validation5_ibrutinib), stratified into groups by IGHV mutation status and trisomy 12 (tris12). **h,** Time to progression of pretreated patients uniformly treated with ibrutinib (Validation5_ibrutinib), stratified into groups by IGHV mutation status and trisomy 12 (tris12). Source data are provided as a Source Data file.

Supplementary Methods

IGHV status analysis

RNA was isolated from 1x10^{7} PBMCs using TRIZOL reagent (Thermo Fisher Scientific) according to manufacturer's instructions. cDNA was synthesized from 2 µg RNA using High-capacity cDNA Reverse Transcription Kit (Thermo Fisher Scientific) according to manufacturer's instructions. PCR reactions as well as the analyses were performed with minor modifications[3]. For PCR reactions AmpliTaq Gold DNA polymerase (Thermo Fisher Scientific) with 0.2 µM of each primer and 0.2 mM of each dNTP was used. VH1-, VH3- and VH4- segments were amplified in single reactions whereas primers for VH2, VH3-21, VH5 as well as VH6-segments were run in a multiplex PCR reaction as described [3]. PCR program was as follows: initial denaturation at 94 °C for 2 minutes, followed by 40 cycles of denaturation (94 °C, 20 seconds), annealing (52 °C, 10 seconds) and elongation (72 °C, 30 seconds) and a final elongation step of 2 minutes at 72 °C. PCR products were sent for Sanger Sequencing (GATC Biotech) using the appropriate forward and the JH-1 reverse primer for the sequencing reaction. In the multiplex PCR reaction both JH-rev as well as JH-1 rev were used for sequencing. After sequencing forward and reverse sequencing results were aligned. To determine the closest matching germline VH-sequence as well as the mutation status, i.e. the percentage of sequence identity, of the VH-segment determined the IMGT/V-Quest-Database was used. The primers for individual PCRs were as follows: PCR1: VH1, JH, JH-1; PCR2: VH3, JH, JH-1; PCR3: VH4, JH, JH-1; PCR4: VH2, VH3-21, VH5, VH6, JH, JH-1 [3].

Supplementary table 3: Primers used for the determination of the IGHV status. Also see reference [3]

VH1	5′-CACCATGGACTGGACCTGGA-3′
VH2	5′-ATGGACACACTTTGCTCCAC-3′
VH3	5′-CCATGGAGTTTGGGCTGAGC-3′
VH3-21†	5′-CCATGGAacTgGGGCTccGC-3′
VH4	5′-ATGAAACACCTGTGGTTCTT-3′
VH5	5′-ATGGGGTCAACCGCCATCCT-3′
VH6	5′-ATGTCTGTCTCCTTCCTCAT-3′
JH	5′-ACCTGAGGAGACGGTGACCAGGGT-3′
JH-1	5′-ACCTGAGGAGACGGTGACC-3′

Panel sequencing of CLL samples

For gene mutation analysis of CLL candidate genes we designed a customized Illumina™ TruSeq Custom Amplicon (TSCA) panel with two independent primer sets for a redundant coverage of *NOTCH1, SF3B1, ATM, TP53, RPS15, BIRC3, MYD88, FBXW7, POT1, XPO1, NFKBIE, EGR2 and BRAF* [4]*. For ATM, BIRC3, EGR2, FBXW7, MYD88, NFKBIE, POT1* and *TP53* the full gene was covered. For *BRAF* (exons 11-18), *NOTCH1* (exon 34 +3'UTR), *RPS15* (exons 3-4), *SF3B1* (exons 14-16) and XPO1 (exons 14-17) the most commonly affected exons were covered. The selection of these targets comprises the 11 most frequently mutated genes in CLL identified via unbiased whole exome sequencing of 528 CLL patients [5]. Library preparation was performed using TruSeq Custom Amplicon Assay Kit v1.5 including extension and ligation steps between custom probes. Samples were indexed, pooled and loaded on an Illumina MiSeq flowcell in 32 sample batches.

The cumulative target size was 41,352 basepairs (bp) covered with 304 amplicons in each panel with an amplicon length up to 250 bp. Adjacent 5 intron bp were included to cover splice site mutations. Input of 250 ng DNA from peripheral blood mononuclear cells was sufficient for libraries according to the Illumina TSCA protocol.

We used a custom bioinformatics pipeline including BWA and Samtools (alignment; [6]), and Varscan (variant calling and annotation; [7]). Current databases (COSMIC [8], 1000G [9], dbSNP145 [10], ClinVar [11]) were taken into consideration to evaluate and report variants above a threshold of 5 % mean variant allele fraction (VAF) as pathogenic/non pathogenic. Only mutations which occurred in at least three patients and an allele frequency of more than 20 % were considered for further analyses.

In-depth data dependant acquisition mass spectrometry proteomics using HiRIEF

Cell pellets were dissolved in Lysis buffer (4 % SDS, 50 mM HEPES pH 7.6, 1 mM DTT), heated to 95° C and sonicated. The total protein amount was estimated (Bio-Rad DC). Samples were then prepared for mass spectrometry analysis using a modified version of the SP3 protein clean-up and a digestion protocol [12,13], where proteins were digested by LysC and trypsin (sequencing grade modified, Pierce). In brief, up to 250 µg protein from each sample was alkylated with 4 mM Chloroacetamide. Sera-Mag SP3 bead mix (20 µl) was transferred into the protein sample together with 100 % Acetonitrile to a final concentration of 70 %. The mix was incubated under rotation at room temperature for 18 min. The mix was placed on the magnetic rack and the supernatant was discarded, followed by two washes with 70 % ethanol and one with 100 % acetonitrile. The beads-protein mixture was reconstituted in 100 µl LysC buffer (0.5 M Urea, 50 mM HEPES pH: 7.6 and 1:50 enzyme (LysC) to protein ratio) and incubated overnight. Finally, trypsin was added in 1:50 enzyme to protein ratio in 100 µl 50 mM HEPES pH 7.6 and incubated overnight. The peptides were eluted from the mixture after placing the mixture on a magnetic rack, followed by peptide concentration measurement (Bio-Rad DC Assay). The samples were then pH adjusted using TEAB pH 8.5 (100 mM final conc.), 65 µg of peptides from each sample were labelled with isobaric TMT-tags (TMT10plex reagent) according to the manufacturer's protocol (Thermo

Scientific). Each set consisted of 9 individual patient samples and the tenth channel contained the same sample pool in each set, consisting of a mixture of patient samples. Sample pools were used as denominators when calculating TMT-ratios and thus served to link the 8 sets together.

Of note, the labelling efficiency was determined by LC-MS/MS before pooling of the samples. After pooling samples, a solid phase extraction (SPE strata-X-C, Phenomenex) was performed and purified samples were dried in a SpeedVac. An aliquot of approximately 10 µgwas suspended in LC mobile phase A and 1 µg was injected on the LC-MS/MS system.

The tryptic peptides for each pooled set (325 µg) were separated by immobilized pH gradient - isoelectric focusing (IPG-IEF) on 3–10 strips[14]. Briefly, dried peptides were solubilized in 250 µl of 8M Urea with 1% Pharmalyte pH 3-10 (GE Healthcare) and left to soak into a 3-10 isoelectric focusing gel strip overnight. Gel strips were then focused in an Ettan IPGphor for at least 48 hours (>150 000 Vh). During the first 5 hours the voltage was slowly ramped up to 8000 V and then kept there for the duration of the focusing.

After focusing the peptides were eluted from the gel strips into distinct fractions by placing a 72 well comb over the gel strip and eluting peptides with 3 rounds of elution: first water only, then 40% ACN and last 40% ACN, 0.1% FA. Eluted fractions were dried and stored at -20 degrees until MS-analysis.

For a detailed description and step-by-step guidelines see [15]

Online LC-MS was performed[14,16] using a Dionex UltiMate™ 3000 RSLCnano System coupled to a Q-Exactive-HF mass spectrometer (Thermo Scientific).

Each of the 72 plate wells was dissolved in 20 µl solvent A and 10 µl were injected. Samples were trapped on a C18 guard-desalting column (Acclaim PepMap 100, 75 µm x 2 cm, nanoViper, C18, 5 µm, 100 Å), and separated on a 50 cm long C18 column (Easy spray PepMap RSLC, C18, 2 µm, 100 Å, 75 µm x 50 cm). The nano capillary solvent A was 95 % water, 5 % DMSO, 0.1 % formic acid; and solvent B was 5 % water, 5 % DMSO, 95 % acetonitrile, 0.1 % formic acid. At a constant flow of 0.25 µl min^{-1}, the curved gradient went from 6-8 % B up to 40 % B in each fraction in a dynamic range of gradient length (see supplementary table 4), followed by a steep increase to 100 % B in 5 min. FTMS master scans with 60,000 resolution (and mass range 300-1500 m/z) were followed by data-dependent MS/MS (30 000 resolution) on the top 5 ions using higher energy collision dissociation (HCD) at 30 % normalized collision energy. Precursors were isolated with a 2 m/z window. Automatic gain control (AGC) targets were $1e^6$ for MS1 and $1e^5$ for MS2. Maximum injection times were 100 ms for MS1 and 100 ms for MS2. The entire duty cycle lasted ~2.5 s. Dynamic exclusion was used with 30 s duration. Precursors with unassigned charge state or charge state 1 were excluded. An underfill ratio of 1 % was used.

Protein and peptide identification and quantification was carried out as previously described [14,16]. Briefly, Orbitrap raw MS/MS files were converted to mzML format using msConvert from

the ProteoWizard tool suite . Spectra were then searched using MSGF+ (v10072) and Percolator (v2.08) , where search results from 8 subsequent fractions were grouped for Percolator target/decoy analysis. All searches were done against the human protein subset of Ensembl 75 in the Galaxy platform. MSGF+ settings included precursor mass tolerance of 10 ppm, fully-tryptic peptides, maximum peptide length of 50 amino acids and a maximum charge of 6. Fixed modifications were TMT-10plex on lysines and peptide N-termini, and carbamidomethylation on cysteine residues; a variable modification was used for oxidation on methionine residues. Quantification of TMT-10plex reporter ions was done using OpenMS project's IsobaricAnalyzer (v2.0). PSMs found at 1 % FDR (false discovery rate) were used to infer gene identities.

Protein quantification by TMT10plex reporter ions was calculated using TMT PSM ratios to the entire sample set (all 10 TMT-channels) and normalized to the sample median. The median PSM TMT reporter ratio from peptides unique to a gene symbol was used for quantification. Protein false discovery rates were calculated using the picked-FDR method using gene symbols as protein groups and limited to 1% FDR.

Supplementary table 4: Table describing dynamic LC gradient lengths used for analysis of individual HiRIEF fractions by LC-MS/MS.

Sample	gradient length	Sample	gradient length
fraction_01	50 min	fraction_37	50 min
fraction_02	50 min	fraction_38	70 min
fraction_03	70 min	fraction_39	70 min
fraction_04	70 min	fraction_40	50 min
fraction_05	90 min	fraction_41	50 min
fraction_06	90 min	fraction_42	50 min
fraction_07	110 min	fraction_43	accumulate in trap column
fraction_08	110 min	fraction_44	accumulate in trap column
fraction_09	110 min	fraction_45	accumulate in trap column
fraction_10	110 min	fraction_46	accumulate in trap column
fraction_11	110 min	fraction_47	accumulate in trap column
fraction_12	90 min	fraction_48	accumulate in trap column
fraction_13	90 min	fraction_49	accumulate in trap column
fraction_14	90 min	fraction_50	50 min
fraction_15	90 min	fraction_51	70 min
fraction_16	70 min	fraction_52	70 min
fraction_17	50 min	fraction_53	accumulate in trap column
fraction_18	50 min	fraction_54	accumulate in trap column
fraction_19	50 min	fraction_55	accumulate in trap column
fraction_20	accumulate in trap column	fraction_56	accumulate in trap column
fraction_21	accumulate in trap column	fraction_57	accumulate in trap column
fraction_22	accumulate in trap column	fraction_58	accumulate in trap column
fraction_23	accumulate in trap column	fraction_59	accumulate in trap column
fraction_24	accumulate in trap column	fraction_60	accumulate in trap column
fraction_25	accumulate in trap column	fraction_61	accumulate in trap column
fraction_26	accumulate in trap column	fraction_62	accumulate in trap column
fraction_27	50 min	fraction_63	accumulate in trap column
fraction_28	50 min	fraction_64	50 min
fraction_29	70 min	fraction_65	50 min
fraction_30	50 min	fraction_66	70 min
fraction_31	50 min	fraction_67	accumulate in trap column
fraction_32	accumulate in trap column	fraction_68	accumulate in trap column
fraction_33	accumulate in trap column	fraction_69	accumulate in trap column
fraction_34	accumulate in trap column	fraction_70	accumulate in trap column
fraction_35	accumulate in trap column	fraction_71	50 min
fraction_36	50 min	fraction_72	50 min

Mass Spectrometry - DIA-based proteomics

Each sample was dissolved in 200 µl lysis buffer (25 mM HEPES pH 7.6, 4 % SDS, 1 mM DTT), heated at 90° C for 5 min and sonicated for 1 min. The total protein amount was estimated (Bio-Rad DC). Samples were then prepared for mass spectrometry analysis using a modified version of the SP3 protein clean-up and a digestion protocol [12,13], where proteins were digested by LycC and trypsin (sequencing grade modified, Pierce). In brief, 200 µg (or the entire amount if <200 µg protein was available) from each sample was alkylated with 4 mM Chloroacetamide. Sera-Mag SP3 (GE Healthcare products 45152105050250 and 65152105050250, distributed by Thermo Fisher) bead mix (20 µl) was transferred into the protein sample together with 100% Acetonitrile to a final concentration of 70 %. The mix was incubated under rotation at room temperature for 20 min. The mix was placed on the magnetic rack and the supernatant was discarded, followed by two washes with 70 % ethanol and one with 100 % acetonitrile. The beads-protein mixture was reconstituted in 100 µl LycC buffer (0.5 M Urea, 50 mM HEPES pH: 7.6 and 1:50 enzyme (LycC) to protein ratio) and incubated overnight. Finally, trypsin was added in 1:50 enzyme to protein ratio in 100 µl 50 mM HEPES pH 7.6 and incubated overnight. Peptide concentration was measured using Bio-Rad DCC.

50 µg of peptides from each sample were cleaned by SP3 beads. For that, peptides were dried by SpeedVac, and dissolved in 20 µl water. 10 µl beads were added to each tube and mixed by short vortex. 570 µl acetonitrile was added to each sample to reach 95 % ACN. The mixture was incubated for 30 minutes at room temperature. To remove the buffer, the tube was placed on a magnetic rack and incubated for 2 minutes at room temperature. Supernatant was discarded. Magnetic beads were washed by addition of 250 µl of acetonitrile and incubated for 30 seconds on the magnetic stand. Supernatant was discarded and the beads air-dried. Tryptic peptides were detached from the beads by addition of 100 µl of 3 % ACN, 0.1 % FA and transferred to a new tube.

5 µg of peptides from each sample were injected and separated using an Ultimate 3000 RSLCnano system coupled to a Q Exactive HF (Thermo Fischer Scientific, San Jose, CA, USA). Samples were trapped on an Acclaim PepMap nanotrap column (C18, 3 mm, 100 Å, 75 µm x 20 mm, Thermo Scientific), and separated on an Acclaim PepMap RSLC column (C18, 2 µm, 100 Å, 75 µm x 50 cm, Thermo Scientific). Peptides were separated using a gradient of mobile phase A (5 % DMSO, 0.1 % FA) and B (90 % ACN, 5 % DMSO, 0.1 % FA), ranging from 6 % to 30 % B in 180 min with a flow of 0.25 ml/min.

For data independent acquisition (DIA), data was acquired using a variable window strategy. The survey scan was performed at 120,000 resolution from 400-1200 m/z, with a max injection time of 200 ms and target of 1e6 ions. For generation of HCD fragmentation spectra, max ion injection time was set as auto and AGC of 2e5 were used before fragmentation at 28 % normalized collision energy, 30,000 resolution. The sizes of the precursor ion selection windows were optimized to have similar density of precursor m/z. The median size of windows was 18.3 m/z with a range of 15-88 m/z covering the scan range of 400-1200 m/z. Neighbor windows had a 2 m/z overlap.

For protein identification and quantification, all raw files analyzed by Spectronaut using the Direct-DIA option without the use of a spectral library, files were searched against ENSEMBL protein database (GRCh38.98.pep.all.fasta). All parameters were kept as default for protein identification. Briefly, runs were recalibrated using iRT standard peptides in a local and non-linear regression. Precursors, peptides and proteins were filtered with FDR 1 %. The decoy database was created by mutation method. For quantification, only peptides unique to a protein group were used. Protein groups were defined based on gene symbols to obtain a gene symbol centric quantification. Stripped peptide quantification was defined as the top precursor quantity. Protein group quantification was calculated by the median value of the top 3 most abundant peptides. Quantification was performed at the MS2 level based on the peak area. The quantitative values were filtered using the qvalue for each sample. Imputation was not performed at any stage of the quantification data generation.

Protein-protein correlation

Protein complex analysis

Protein core complex information was retrieved from the CORUM website (http://mips.helmholtzmuenchen.de/corum/#download). All complex members were assumed to interact with each other. Protein complex information was converted into a pairwise interaction matrix as previously described [16]. The distribution of correlations between proteins/genes in known complexes in both the transcriptomics and proteomics data was compared to the distribution of correlations between randomly selected protein/gene pairs.

For the investigation of trisomy 12 related complexes, CORUM complexes which contained genes located on chromosome 12 were kept. The log2-value of the relative median abundance of individual proteins in the trisomy 12 cases compared to non-trisomy 12 cases was calculated and complexes with an overall upregulation and no down regulated proteins were kept.

Protein-protein interaction network

In order to identify a protein population with high standard deviation across the samples, we first calculated a modified quantile standard deviation for the log ratios of each full-overlap protein. Therefore, the lowest and highest value was discarded. The distribution of all log quantile standard deviations was then modelled with a mixture of two Gaussian distributions by applying an expectation maximization algorithm (R package mixtools). The converged model with the two underlying distributions was assumed to represent unmodulated and modulated proteins.

Next, we determined the log standard deviation cutoff that optimally separated the modulated from the unmodulated protein population. We probed each percentile as a cutoff for the mixed distributions, calculated the true and false positive rates and averaged the values over ten consecutive runs. The log standard deviation cutoff was selected by optimising the number of true positive minus false positive modulated proteins, eventually rounded to the lower .5 to ensure reproducibility.

Pairwise Pearson correlations between each modulated protein were calculated. In a QQ-plot, we compared our values to a hypothetical normal distribution and roughly set a cutoff where correlation coefficients started to deviate from the diagonal, which was at r = 0.5. All correlations equal or greater than this were translated into edges between protein nodes in an initial protein-protein-interaction network (github code: "ppi_network"). The network was visualized in Gephi 0.9.2. The initial number of nodes (n =1801) were filtered using a Kcore setting of 3 and above and the core network of 1047 nodes was used for further analysis. Modularity clustering of the nodes was carried out with a resolution of 0.8.

Annotation of the modularity clusters was done by first extracting all proteins belonging to a cluster. Next, any protein in the full overlap dataset (n =7313) that had a Pearson correlation above 0.7 to any of the cluster members was included in the target gene set for that cluster. Enrichment using a target-background approach was carried out against the MSigDB categories Hallmark, C1, C2, C5 and C6. The full overlap data set (n =7313) was used as background and the R packages msigdbr and ClusterProfiler were used to calculate enrichments (github code: "enrichment_of_network-msigdb").

Analysis of differential splicing

RNA level

Due to its short computational time the julia-based tool *whippet* [17] was used for visualisation of specific splicing events, as shown in Fig. S6, following the recommended workflow. In brief, an index was created from the human reference genome (GRCh37). Fastq files were quantified using the options for single-end reads. ASB-CLL was compared to all other samples, or *SF3B1* mutated samples were compared to wt samples, by using the *whippet-delta* functions and setting the parameters of *-r* to 20 and *-s* to 3. For visualisation delta percent-spliced-in values were plotted in R.

References

1. Eagle, G. L. *et al.* Total proteome analysis identifies migration defects as a major pathogenetic factor in immunoglobulin heavy chain variable region (IGHV)-unmutated chronic lymphocytic leukemia. *Mol. Cell. Proteomics* **14**, 933–945 (2015).
2. Inoue, D. *et al.* Spliceosomal disruption of the non-canonical BAF complex in cancer. *Nature* **574**, 432–436 (2019).
3. Szankasi, P. & Bahler, D. W. Clinical laboratory analysis of immunoglobulin heavy chain variable region genes for chronic lymphocytic leukemia prognosis. *J. Mol. Diagn.* **12**, 244–249 (2010).
4. Tausch, E. *et al.* Prognostic and predictive impact of genetic markers in patients with CLL treated with obinutuzumab and venetoclax. *Blood* **135**, 2402–2412 (2020).
5. Landau, D. A. *et al.* Mutations driving CLL and their evolution in progression and relapse. *Nature* **526**, 525–530 (2015).
6. Li, H. A statistical framework for SNP calling, mutation discovery, association mapping and population genetical parameter estimation from sequencing data. *Bioinformatics* **27**, 2987–2993 (2011).
7. Koboldt, D. C. *et al.* VarScan 2: somatic mutation and copy number alteration discovery in cancer by exome sequencing. *Genome Res.* **22**, 568–576 (2012).
8. Tate, J. G. *et al.* COSMIC: the Catalogue Of Somatic Mutations In Cancer. *Nucleic Acids Res.* **47**, D941–D947 (2019).
9. Voight, B. F. *et al.* A global reference for human genetic variation. *Nature* **526**, 68–74 (2015).
10. Kitts, A., Phan, L., Ward, M. & Holmes, J. B. The Database of Short Genetic Variation (dbSNP). in *The NCBI Handbook [Internet]. 2nd edition* (National Center for Biotechnology Information (US), 2014).
11. Landrum, M. J. *et al.* ClinVar: improving access to variant interpretations and supporting

evidence. *Nucleic Acids Res.* **46**, D1062–D1067 (2018).

12. Moggridge, S., Sorensen, P. H., Morin, G. B. & Hughes, C. S. Extending the Compatibility of the SP3 Paramagnetic Bead Processing Approach for Proteomics. *J. Proteome Res.* **17**, 1730–1740 (2018).
13. Hughes, C. S. *et al.* Ultrasensitive proteome analysis using paramagnetic bead technology. *Mol. Syst. Biol.* **10**, 757 (2014).
14. Branca, R. M. M. *et al.* HiRIEF LC-MS enables deep proteome coverage and unbiased proteogenomics. *Nat. Methods* **11**, 59–62 (2014).
15. Arslan, T. *et al.* SubCellBarCode: integrated workflow for robust spatial proteomics by mass spectrometry. *Nat. Protoc.* **17**, 1832–1867 (2022).
16. Johansson, H. J. *et al.* Breast cancer quantitative proteome and proteogenomic landscape. *Nat. Commun.* **10**, 1600 (2019).
17. Sterne-Weiler, T., Weatheritt, R. J., Best, A. J., Ha, K. C. H. & Blencowe, B. J. Efficient and Accurate Quantitative Profiling of Alternative Splicing Patterns of Any Complexity on a Laptop. *Mol. Cell* **72**, 187–200.e6 (2018).
