# Supplementary material for: Proteogenomics refines the molecular classification of chronic lymphocytic leukemia
Source: Nat Commun. 2022 Oct 20;13:6226. doi: 10.1038/s41467-022-33385-8 (PMC9584885; doi:10.1038/s41467-022-33385-8)
Supplement: Supplementary file 2 — Reporting Summary [file 41467_2022_33385_MOESM2_ESM.pdf]

nature portfolio

Corresponding author(s): Janne Lehtiö
Sascha Dietrich

Last updated by author(s): Aug 6, 2022

Reporting Summary

Nature Portfolio wishes to improve the reproducibility of the work that we publish. This form provides structure for consistency and transparency in reporting. For further information on Nature Portfolio policies, see our Editorial Policies and the Editorial Policy Checklist.

Statistics

For all statistical analyses, confirm that the following items are present in the figure legend, table legend, main text, or Methods section.

n/a	Confirmed	
☐	☒	The exact sample size (*n*) for each experimental group/condition, given as a discrete number and unit of measurement
☐	☒	A statement on whether measurements were taken from distinct samples or whether the same sample was measured repeatedly
☐	☒	The statistical test(s) used AND whether they are one- or two-sided<br>*Only common tests should be described solely by name; describe more complex techniques in the Methods section.*
☐	☒	A description of all covariates tested
☐	☒	A description of any assumptions or corrections, such as tests of normality and adjustment for multiple comparisons
☐	☒	A full description of the statistical parameters including central tendency (e.g. means) or other basic estimates (e.g. regression coefficient) AND variation (e.g. standard deviation) or associated estimates of uncertainty (e.g. confidence intervals)
☐	☒	For null hypothesis testing, the test statistic (e.g. *F*, *t*, *r*) with confidence intervals, effect sizes, degrees of freedom and *P* value noted<br>*Give P values as exact values whenever suitable.*
☒	☐	For Bayesian analysis, information on the choice of priors and Markov chain Monte Carlo settings
☒	☐	For hierarchical and complex designs, identification of the appropriate level for tests and full reporting of outcomes
☐	☒	Estimates of effect sizes (e.g. Cohen's *d*, Pearson's *r*), indicating how they were calculated

Our web collection on statistics for biologists contains articles on many of the points above.

Software and code

Policy information about availability of computer code

Data collection	Mass Spectras were collected with Thermo Xcalibur v4.0.27.19. MSGF+ and Percolator in the Galaxy platform were used to match MS spectra to the Ensembl92 human protein database.
Data analysis	All statistical analyses were performed in R (version 4.0.2). Library size normalization, variance stabilizing transformation and differential expression calling were performed using DESeq2 (version 1.28.1). Differential protein abundance between samples with different genetic alterations was assessed with limma (version 3.44.3) 52 and DEqMS (version 1.6.0). RNASeq reads were aligned to GRCh 37.75/hg 19 using STAR (v2.6.0c) and counted with htseq-count. Aligned RNA-seq data were then subjected to quality control by RNASeQC (v1.1.8) to ensure the integrity of the transcriptome dataset. Our alternative splicing analyses largely relied on the rMATS tool. Enrichment analysis was performed against KEGG genesets using GSEA (version 4.0.3). The multi-omics factor analysis was performed on genetics, transcriptomics, and proteomics datasets using the MOFA R package (version 1.0.0). Proportional hazards regression (Cox regression) was used to explore the potential impact of protein abundances on TTNT using the R package survival (version 3.2-3). To draw Kaplan-Meier curves the survminer package (version 0.4.8) was used. Consensus clustering on proteins or transcripts was performed using the ConsensusClusterPlus package (version 1.52.0). Dimensionality reduction was done by T-distributed stochastic neighbor embedding (t-SNE; Rtsne package, version 0.15), principal component analysis (stats package), and hierarchical clustering (pheatmap package, version 1.0.12). Statistical tests were performed as indicated in the text and figures. Wilcoxon signed-rank tests were always two-sided. Boxplots are defined as follows: center line, median; box limits, upper and lower quartiles; whiskers, 1.5x interquartile range. The code not accessing patient-sensitive information has been deposited on github: https://github.com/DietrichLab/Proteogenomics_and_drug_response_CLL

For manuscripts utilizing custom algorithms or software that are central to the research but not yet described in published literature, software must be made available to editors and reviewers. We strongly encourage code deposition in a community repository (e.g. GitHub). See the Nature Portfolio guidelines for submitting code & software for further information.

Data

Policy information about availability of data

All manuscripts must include a data availability statement. This statement should provide the following information, where applicable:

- Accession codes, unique identifiers, or web links for publicly available datasets
- A description of any restrictions on data availability
- For clinical datasets or third party data, please ensure that the statement adheres to our policy

The mass spectrometry proteomics data have been deposited to the ProteomeXchange Consortium via the PRIDE partner repository with the dataset identifiers PXD017453 (Discovery set) and PXD024544 (DIA Validation set). The RNAseq data for the discovery cohort is available through the European Genome-Phenome Archive through accession number EGAS00001005746 and for the Validation3_RNA cohort through accession number EGAS00001001746. The data can be easily explored through our web application: https://www.imbi.uni-heidelberg.de/dietrichlab/CLL_Proteomics/

Human research participants

Policy information about studies involving human research participants and Sex and Gender in Research.

Reporting on sex and gender	Sex of patients is reported in the manuscript. No sex specific analyses were performed.
Population characteristics	Patients with chronic lymphocytic leukemia who were able and willing to donate viable CLL cells for research purpose. A detailed description of the population charactersitics for the patient cohorts used in this study is available in supplementary figure 1 in the manuscript. Patients were included in the analysis, regardsless of treatment status. Males and females were included in the study. The patients harboured different genetic alterations, described in detail in the manuscript.
Recruitment	Through the biobank of the Department of Hematology and Oncology, University Hospital Heidelberg. The only inclusion criteria was the ability to obtain enough cancer cells from patient blood to perform proteomic analysis.
Ethics oversight	The collection of samples and clinical data was approved by the ethics commission of the medical faculty of the University of Cologne (13-091), the department of hematology Heidelberg (Ethics vote S-686/2018) and the Stockholm Regional Ethics Board (2006/964-31/2 and 99-154).

Note that full information on the approval of the study protocol must also be provided in the manuscript.

Field-specific reporting

Please select the one below that is the best fit for your research. If you are not sure, read the appropriate sections before making your selection.

[x] Life sciences   [ ] Behavioural & social sciences   [ ] Ecological, evolutionary & environmental sciences

For a reference copy of the document with all sections, see nature.com/documents/nr-reporting-summary-flat.pdf

Life sciences study design

All studies must disclose on these points even when the disclosure is negative.

Sample size	Samples sizes were based on availability of patient material.
Data exclusions	Whenever samples were excluded from the analysis because of insufficient material this is stated in the methods section. For the proteomics data four samples were excluded after quality control assessment due to either poor correlation to the in-depth data (2 samples, discovery cohort) or a low number of identifications (2 samples, Validation1_DIA). Patients were excluded from specific analyses if the respective data was not available, e.g. clinical follow up data.
Replication	A large patient cohort was used (biological replicates). Patient samples were not analysed in replicates. The findings from the discovery cohort were confirmed in 5 independant validation cohorts. All attempts of validation are reported in the manscript and were successful.
Randomization	Patient samples were not allocated into proteomics runs randomly. To be able to perform balanced proteomics runs some prior knowledge of the patient samples i.e. gender, IGHV status, trisomy 12 was required. This was done to prevent batch effects from confounding the analysis. For the drug screen data and sample preparation for proteomics, RNASeq and genetic profiling samples were allocated into groups randomly.
Blinding	During the experimental procedures not much prior knowledge about the patient samples was available (only gender, IGHV status and for some patients trisomy 12 status). Therefore, blinding was not crucial for the experiments. For proteomics runs blinding was not possible, as described above. For data analysis e.g. differential abundance analysis blinding was not possible. Some of the analyses run in our manscript were unsupervised and unbiased.

Reporting for specific materials, systems and methods

We require information from authors about some types of materials, experimental systems and methods used in many studies. Here, indicate whether each material, system or method listed is relevant to your study. If you are not sure if a list item applies to your research, read the appropriate section before selecting a response.

Materials & experimental systems

n/a	Involved in the study	
☒	☐	Antibodies
☒	☐	Eukaryotic cell lines
☒	☐	Palaeontology and archaeology
☒	☐	Animals and other organisms
☒	☐	Clinical data
☒	☐	Dual use research of concern

Methods

n/a	Involved in the study	
☒	☐	ChIP-seq
☒	☐	Flow cytometry
☒	☐	MRI-based neuroimaging
