# Supplementary material for: Proteogenomics refines the molecular classification of chronic lymphocytic leukemia
Source: Nat Commun. 2022 Oct 20;13:6226. doi: 10.1038/s41467-022-33385-8 (PMC9584885; doi:10.1038/s41467-022-33385-8)
Supplement: Supplementary file 3 — Description of Additional Supplementary Files [file 41467_2022_33385_MOESM3_ESM.docx]

**Description of additional supplementary files**

Supplementary data 1: Proteomics dataset discovery cohort

Supplementary data 2: Gene set enrichment analysis results

Supplementary data 3: Analysis of altered exon usage ASB-CLL

Supplementary data 4: Screened drugs

Supplementary data 5: Consensus clustering on protein level

Supplementary data 6: Consensus clustering on mRNA level
