# Supplementary material for: Proteogenomics refines the molecular classification of chronic lymphocytic leukemia
Source: Nat Commun. 2022 Oct 20;13:6226. doi: 10.1038/s41467-022-33385-8 (PMC9584885; doi:10.1038/s41467-022-33385-8)
Supplement: Supplementary file 8 — Supplementary data 5 [file 41467_2022_33385_MOESM8_ESM.pdf]

consensus matrix legend

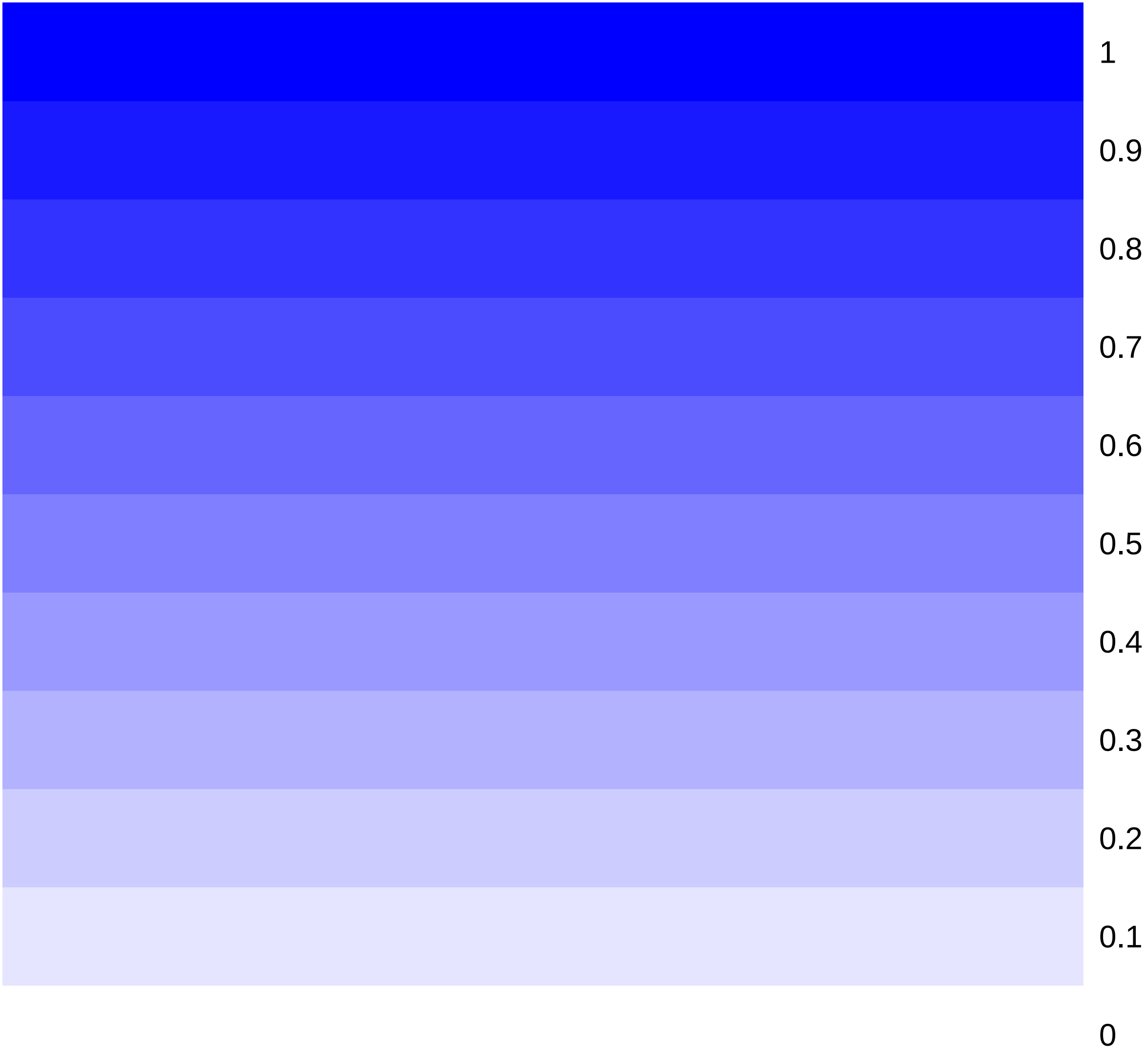

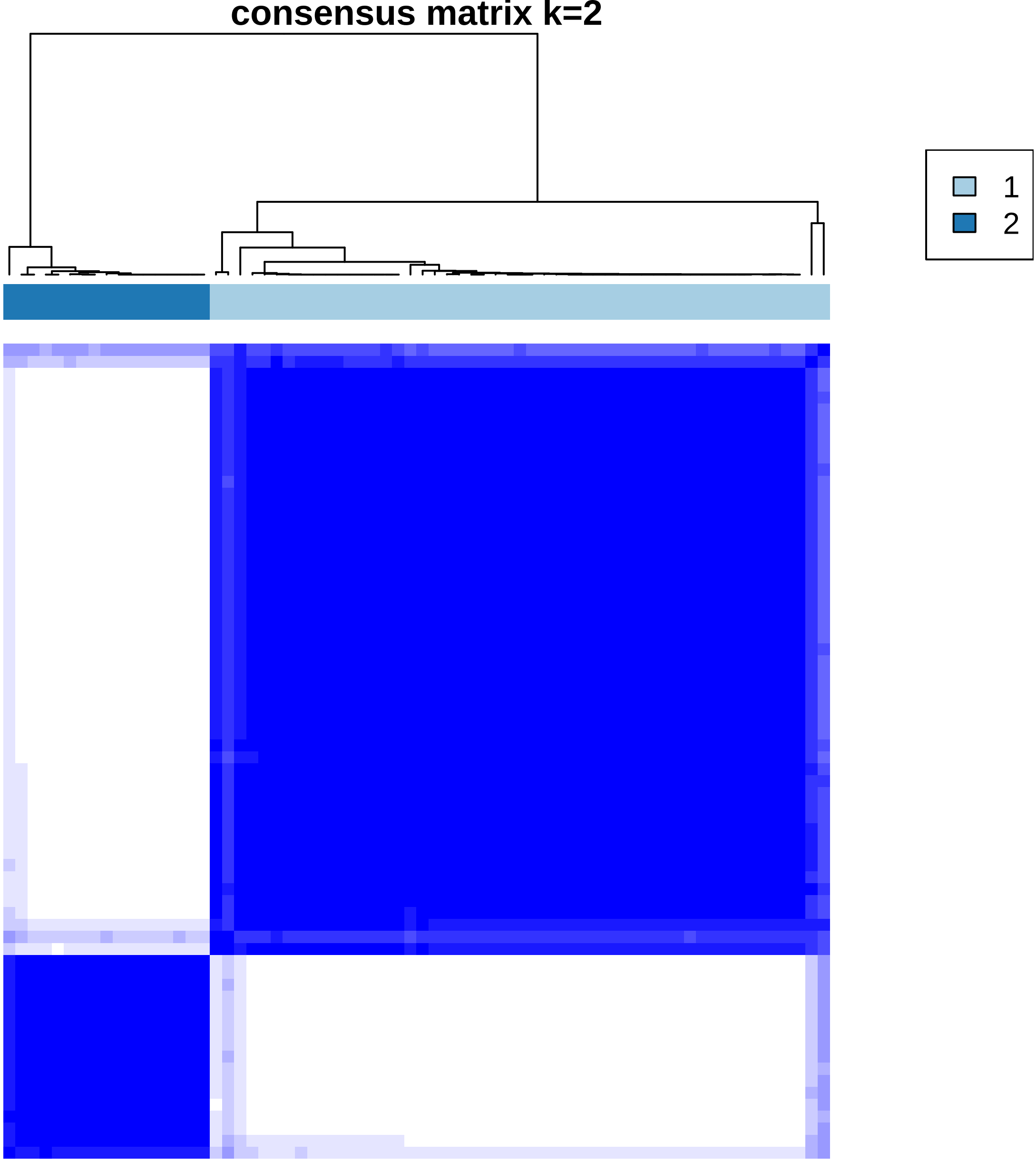
consensus matrix k=2
1
2

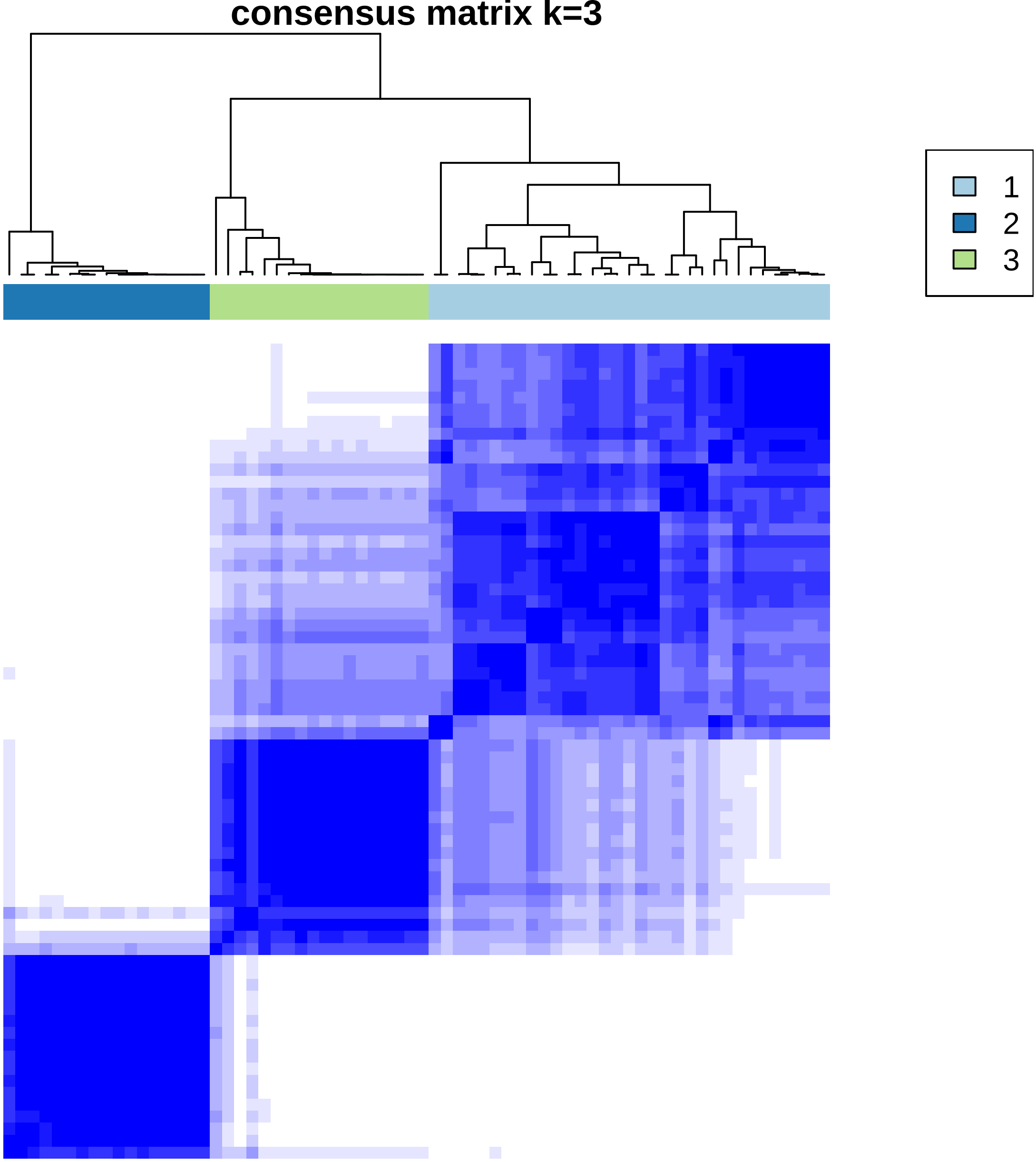
consensus matrix k=3
1
2
3

consensus matrix k=4

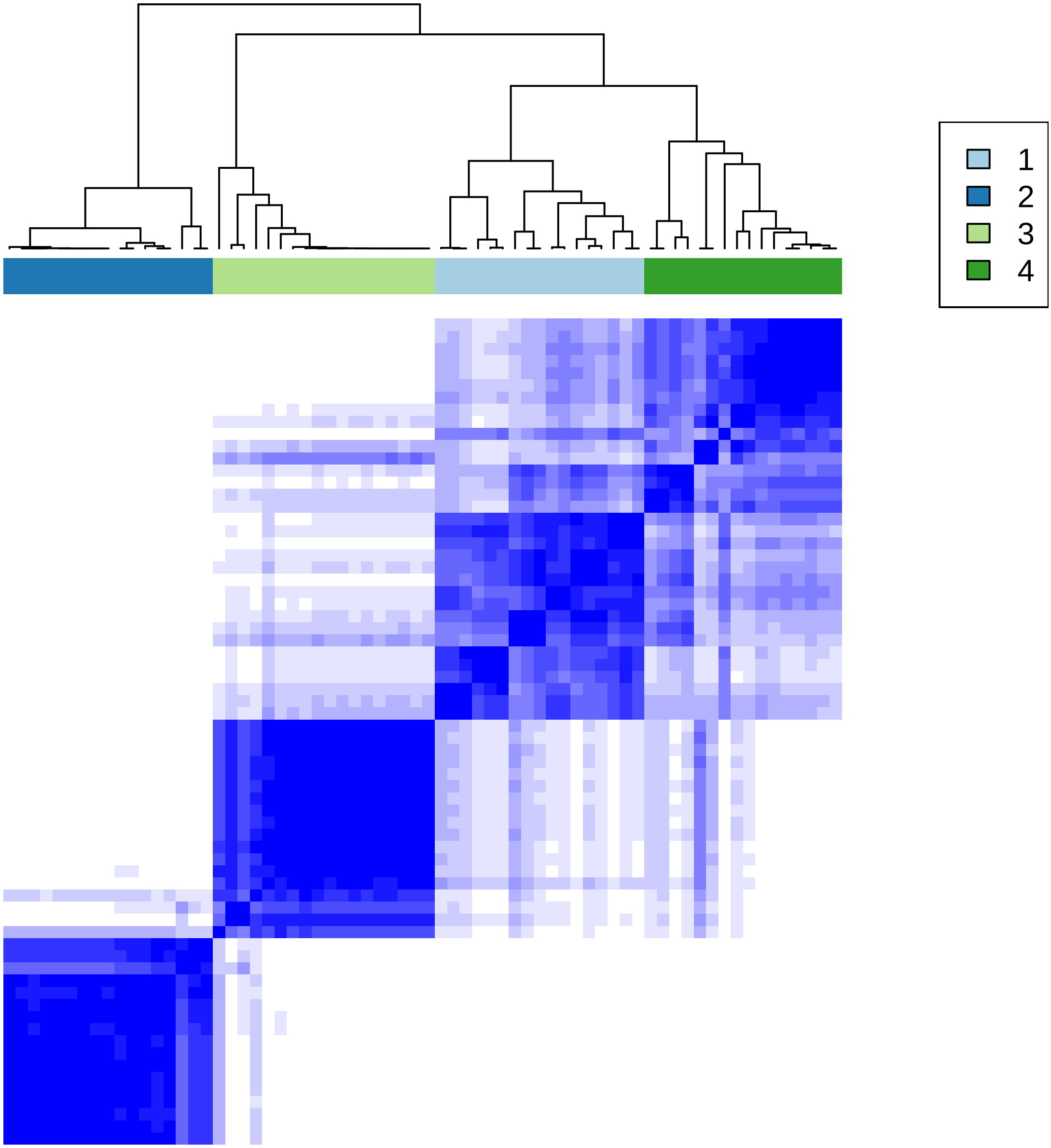

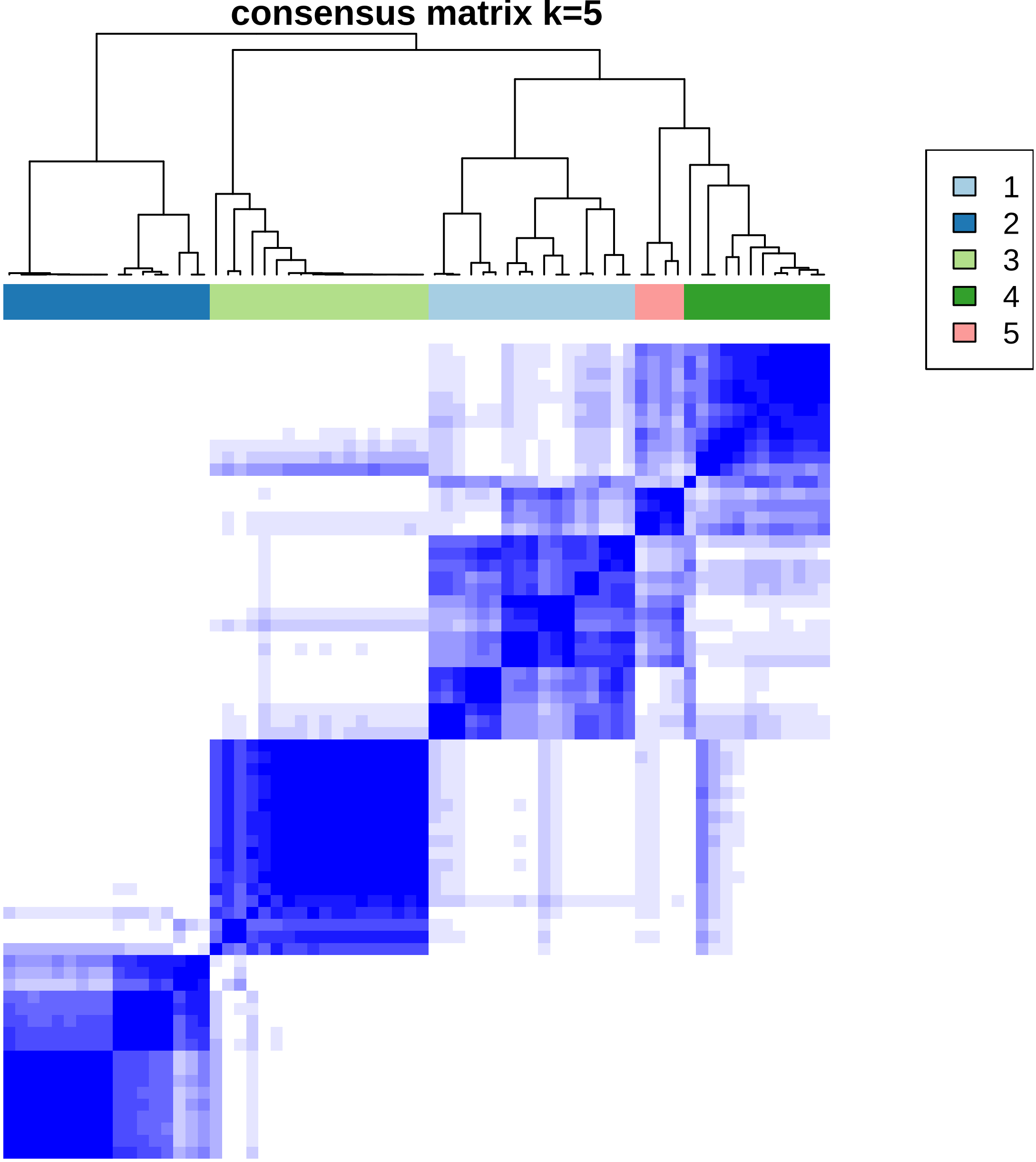
consensus matrix k=5
1
2
3
4
5

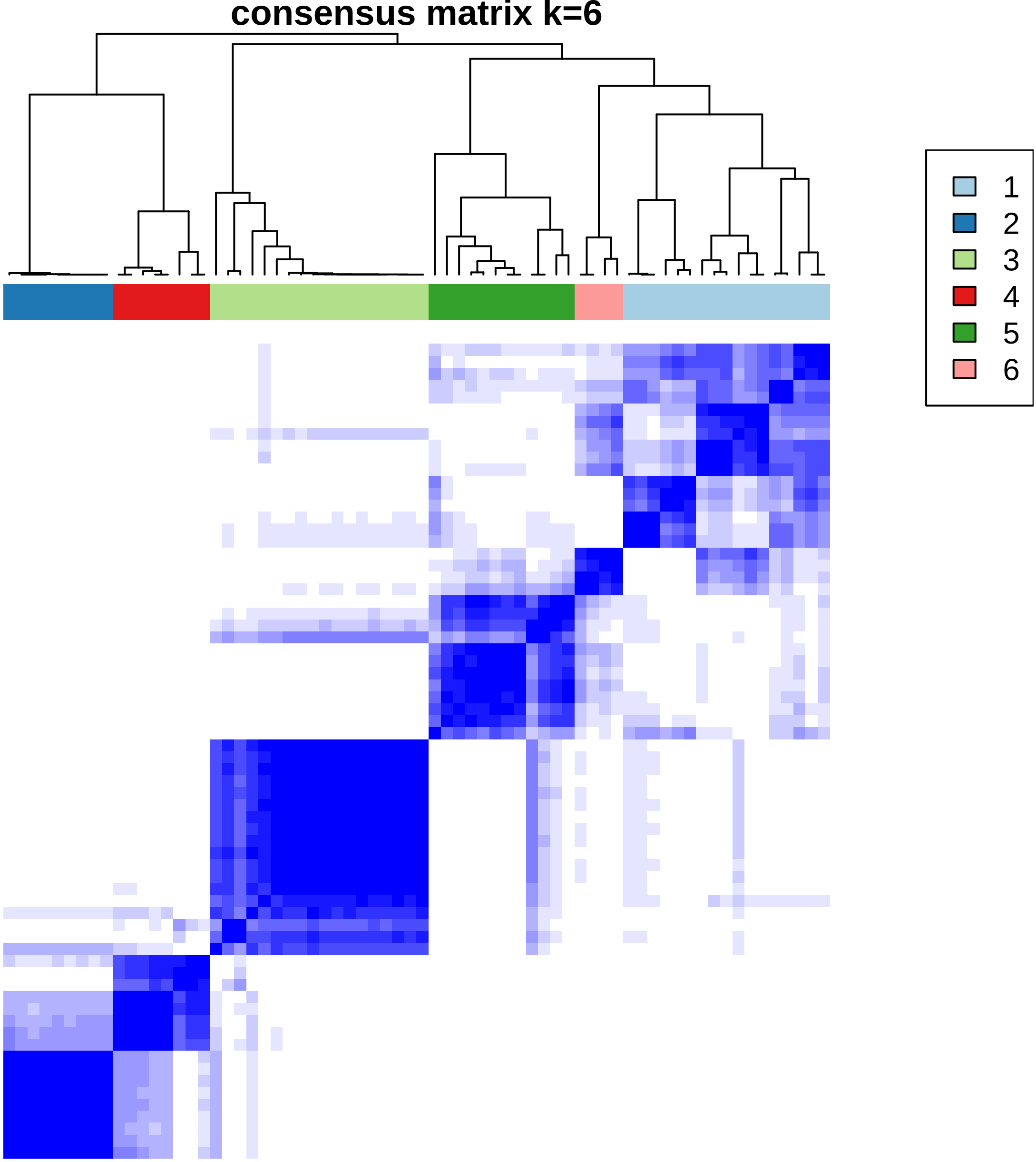
consensus matrix k=6
1
2
3
4
5
6

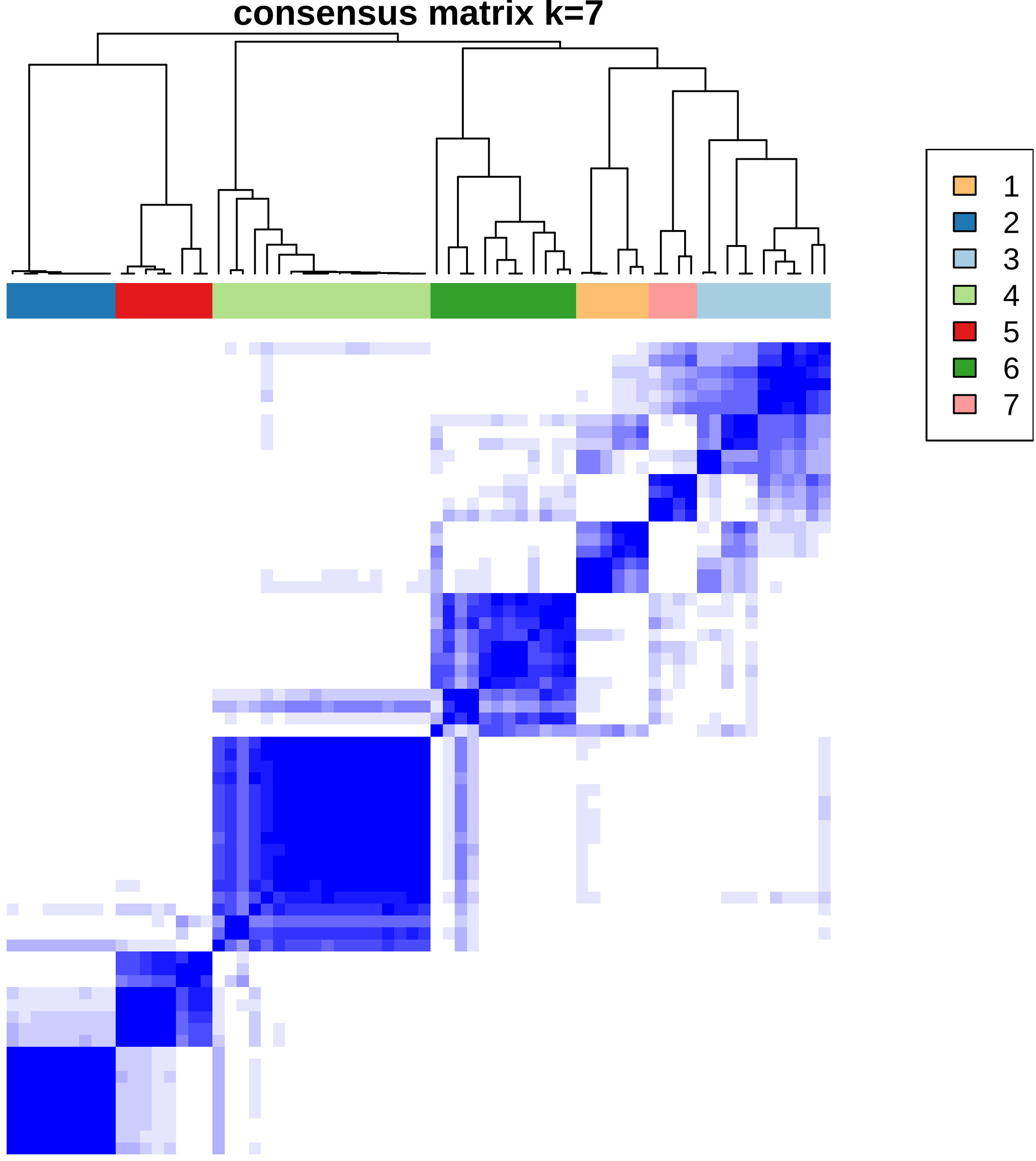
consensus matrix k=7
1
2
3
4
5
6
7

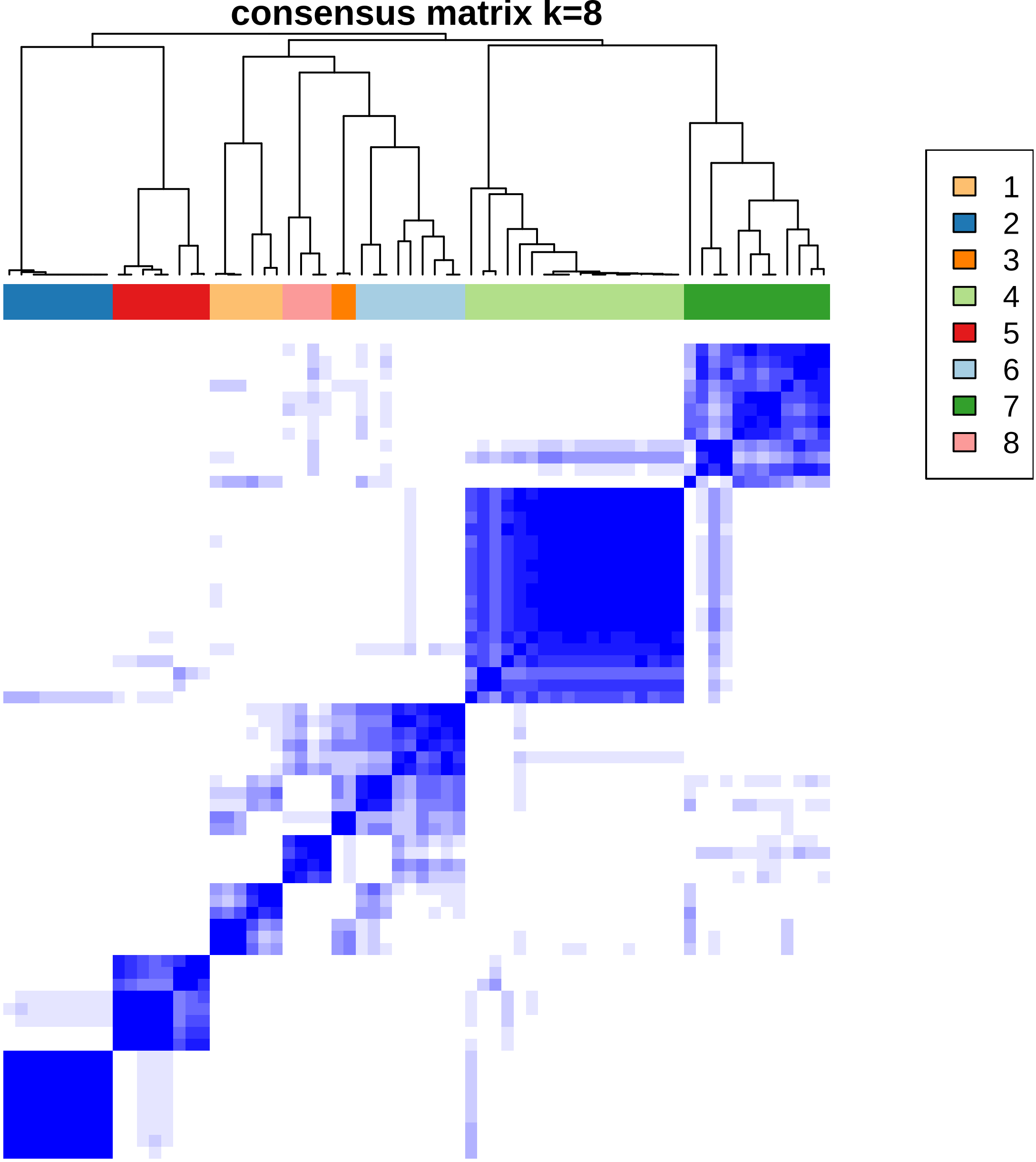
consensus matrix k=8
1
2
3
4
5
6
7
8

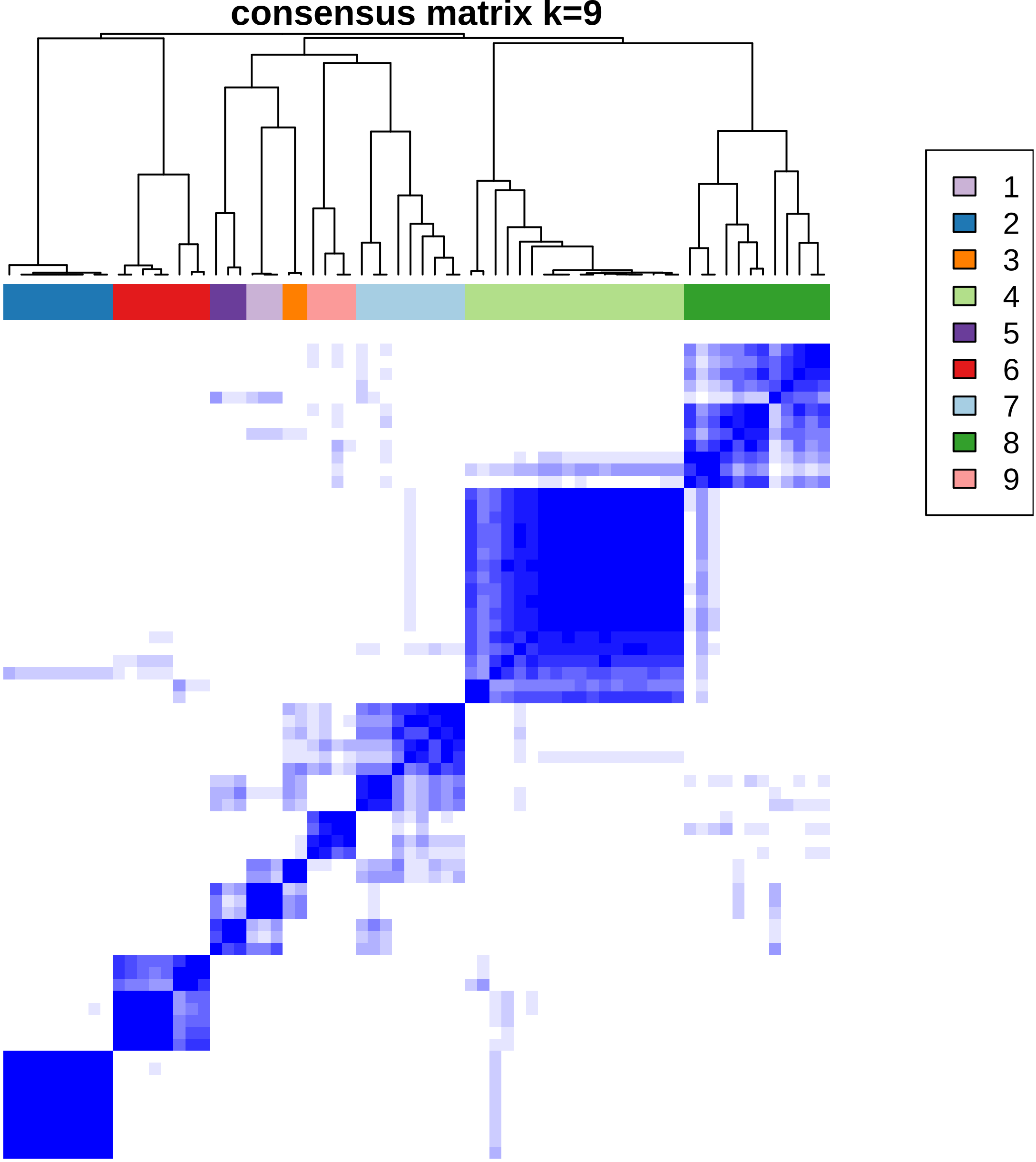
consensus matrix k=9
1
2
3
4
5
6
7
8
9

consensus matrix k=10

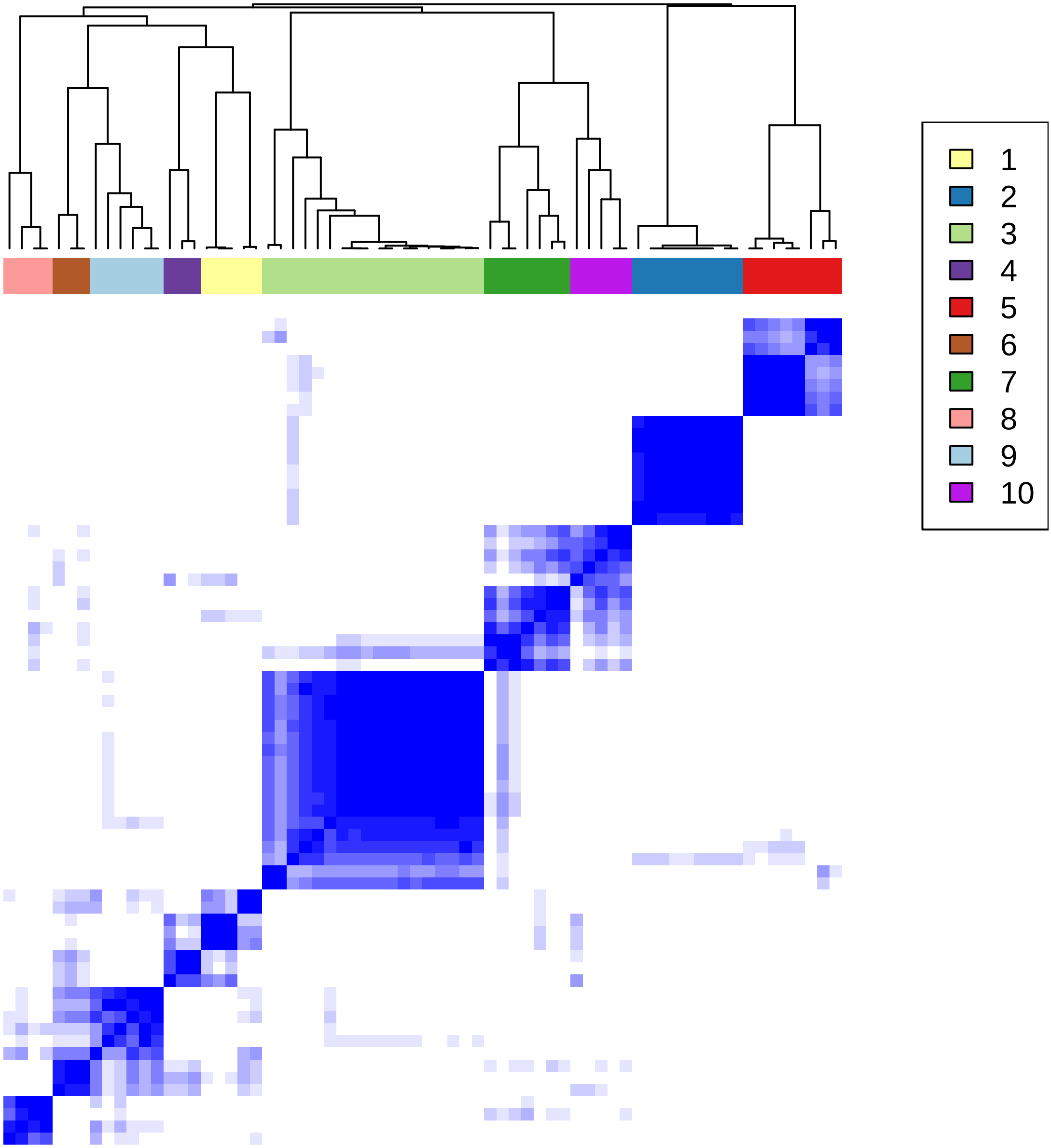

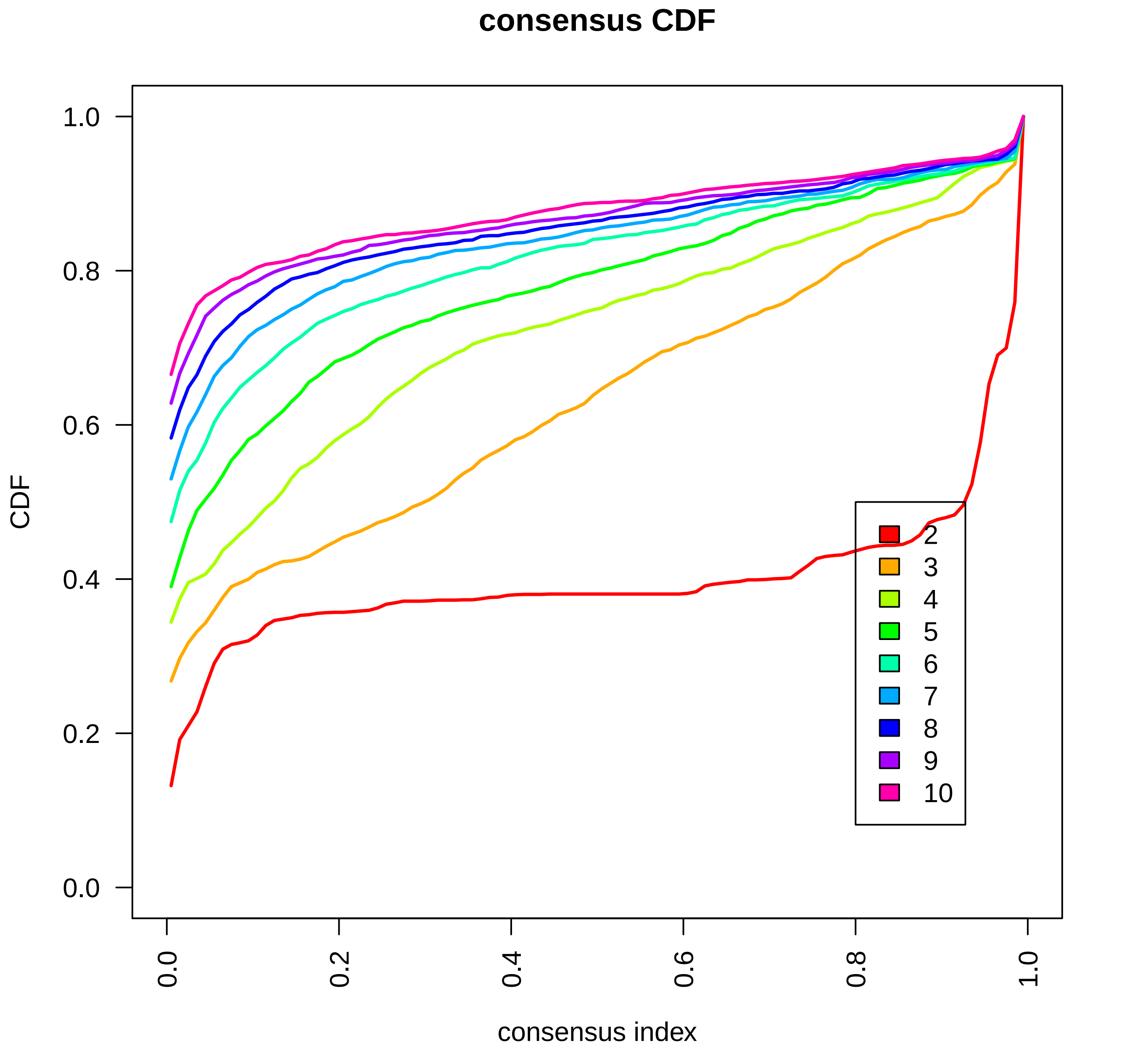

consensus CDF
CDF
consensus index
0.0
0.2
0.4
0.6
0.8
1.0
2
3
4
5
6
7
8
9
10

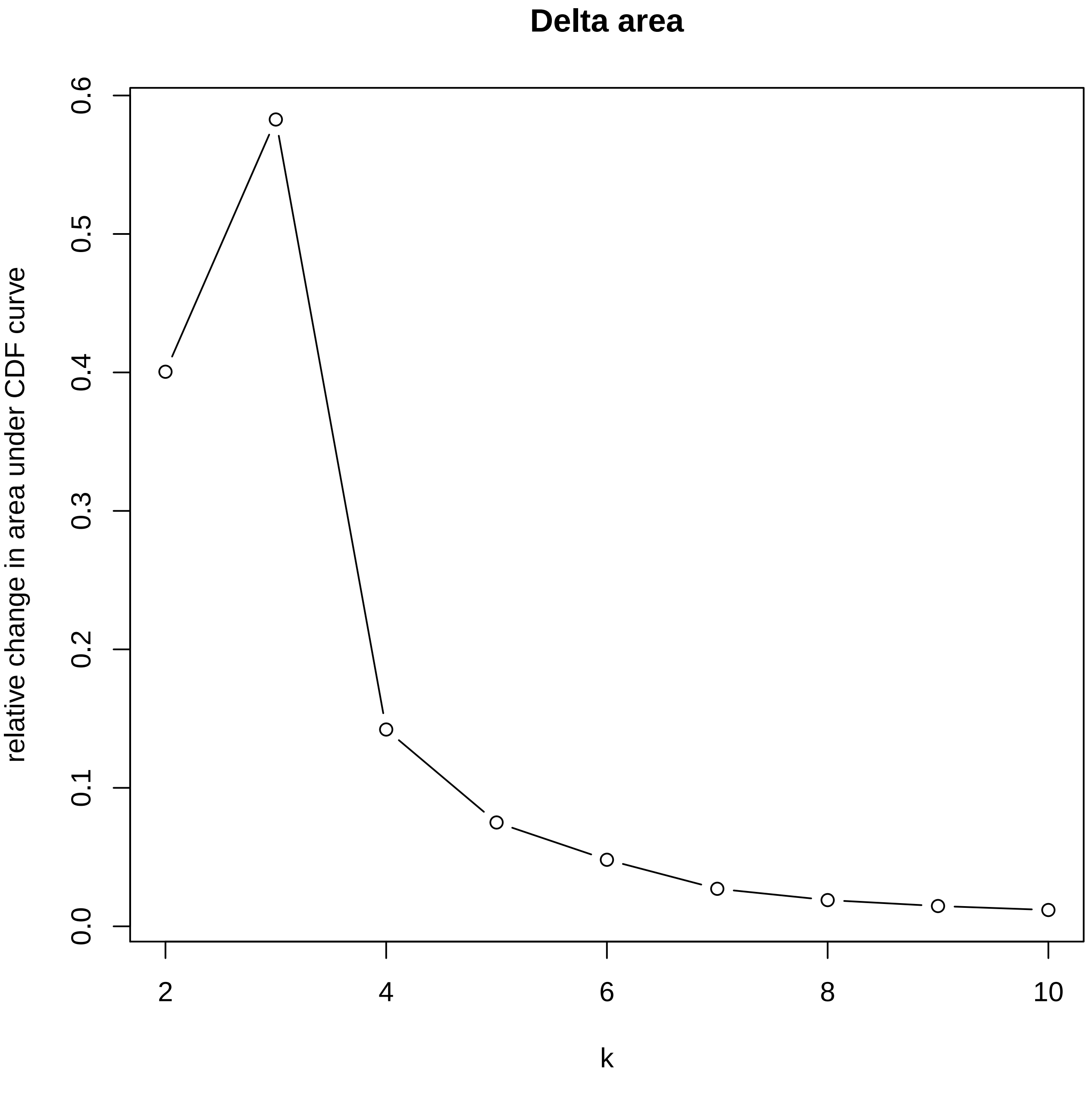
Delta area
relative change in area under CDF curve
k
0.0
0.1
0.2
0.3
0.4
0.5
0.6
2
4
6
8
10

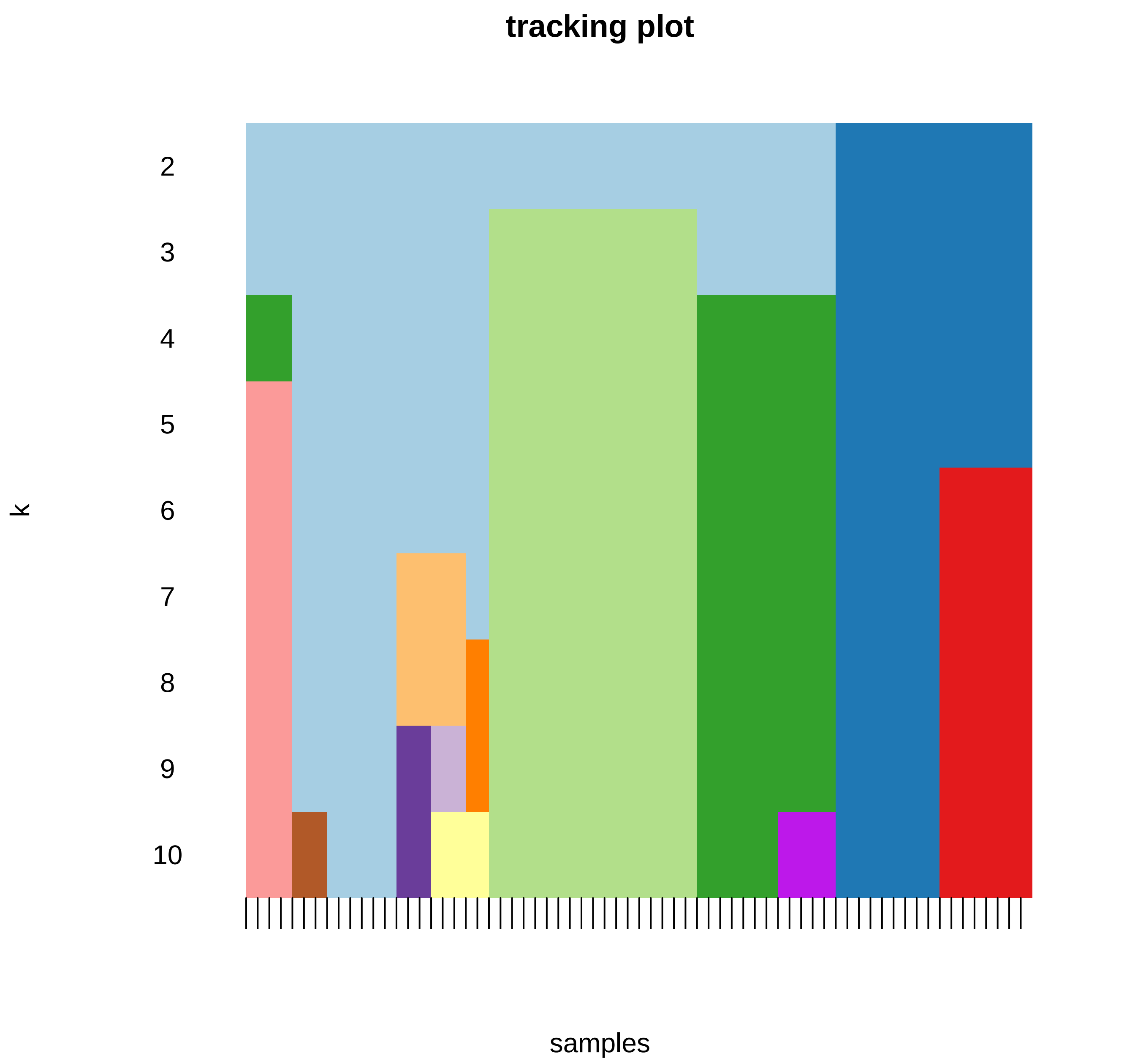
tracking plot
k
2
3
4
5
6
7
8
9
10
samples
